# Novel disease resistance gene paralogs created by CRISPR/Cas9 in soy

**DOI:** 10.1007/s00299-021-02678-5

**Published:** 2021-03-11

**Authors:** Ervin D. Nagy, Julia L. Stevens, Neil Yu, Chris S. Hubmeier, Nona LaFaver, Megan Gillespie, Brian Gardunia, Qianshun Cheng, Steven Johnson, Audrey L. Vaughn, Miguel E. Vega-Sanchez, Mingqui Deng, Linda Rymarquis, Richard J. Lawrence, Graeme S. Garvey, Robert T. Gaeta

**Affiliations:** Bayer Crop Science, 700 Chesterfield Parkway West, Chesterfield, MO 63017 USA

**Keywords:** CRISPR, Cas9, Soy, NBS-LRR, Disease resistance

## Abstract

**Key message:**

Novel disease resistance gene paralogues are generated by targeted chromosome cleavage of tandem duplicated NBS-LRR gene complexes and subsequent DNA repair in soybean. This study demonstrates accelerated diversification of innate immunity of plants using CRISPR.

**Abstract:**

Nucleotide-binding-site-leucine-rich-repeat (NBS-LRR) gene families are key components of effector-triggered immunity. They are often arranged in tandem duplicated arrays in the genome, a configuration that is conducive to recombinations that will lead to new, chimeric genes. These rearrangements have been recognized as major sources of novel disease resistance phenotypes. Targeted chromosome cleavage by CRISPR/Cas9 can conceivably induce rearrangements and thus emergence of new resistance gene paralogues. Two NBS-LRR families of soy have been selected to demonstrate this concept: a four-copy family in the Rpp1 region (Rpp1L) and a large, complex locus, Rps1 with 22 copies. Copy-number variations suggesting large-scale, CRISPR/Cas9-mediated chromosome rearrangements in the Rpp1L and Rps1 complexes were detected in up to 58.8% of progenies of primary transformants using droplet-digital PCR. Sequencing confirmed development of novel, chimeric paralogs with intact open reading frames. These novel paralogs may confer new disease resistance specificities. This method to diversify innate immunity of plants by genome editing is readily applicable to other disease resistance genes or other repetitive loci.

**Supplementary Information:**

The online version contains supplementary material available at 10.1007/s00299-021-02678-5.

## Introduction

Plant immunity against invading pathogens is governed by two tiers of receptors. The transmembrane pattern-recognition receptors (PRRs) recognize conserved pathogen-associated molecular patterns (PAMPs). Unlike in PAMPs-triggered immunity, effector-triggered immunity (ETI) is elicited by highly specific pathogenic effectors. Their cognate receptors are also specific and are under strong diversifying selection (Ellis et al. 2000; Meyers et al. 1999). Most ETI receptors belong to a structurally conserved, yet sequentially diverse superfamily including nucleotide binding site (NBS) and a leucine-rich repeat (LRR) domain (Dangl et al. 2013). Plants include a few dozen to several hundred NBS-LRR (NLR) genes (Ameline-Torregrosa et al. 2008; Cheng et al. 2010, 2012; Christie et al. 2016; Meyers et al. 2003; Monosi et al. 2004; Tan and Wu 2012; Yu et al. 2014). For example, soybean (*Glycine max* L. Merr.) carries 314 putative NLRs (Kang et al. 2012). The majority of NLRs are clustered into tandem duplicated gene islands. This repetitive genomic structure enables frequent generation of new paralogs by rearrangements among duplicates. Chromosomal double-strand breaks (DSBs), through various DNA repair mechanisms, such as non-homologous end joining (NHEJ), single-strand annealing (SSA), synthesis-dependent strand annealing (SDSA) and homologous recombination (HR) are resolved in insertions, deletions, gene conversions, homologous or unequal recombinations (Ceccaldi et al. 2016; Knoll et al. 2014). These rearrangements have been recognized as prime sources of new disease resistance genes (Michelmore and Meyers 1998; Ramakrishna et al. 2002; Ratnaparkhe et al. 2011; Richter et al. 1995; Smith et al. 2004). Specifically, chimeric paralogs, created through recombination of diverged duplicates are believed to be the major class of new molecular determinants of disease resistance.

The rate of these mutations in nature is apparently sufficient to evolve resistance in most wild populations. Whereas substantial number of disease resistance genes were preserved in crops, their diversity were eroded during domestication (Gu et al. 2015; Sakai and Itoh 2010; Zheng et al. 2016), which puts them in disadvantage against rapidly mutating pathogens. Crops with large global footprints are especially vulnerable as they are exposed to a broad diversity of pathogens around the world. This is manifested in daunting worldwide epidemics in several of our staple crops, such as wheat (Singh et al. 2015), soybean (Pivonia and Yang 2004) or banana (Dita et al. 2018), just to name a few.

This paper demonstrates a method to accelerate diversification of repetitive disease resistance loci by targeting them with DSBs using site-directed nucleases and relying on natural DNA repair mechanisms to create new variants. The bacterial CRISPR/Cas9 system (clustered regularly interspaced short palindromic repeat and CRISPR-associated protein 9) is a site-directed endonuclease (Cong et al. 2013; Jinek et al. 2012), which has been successfully used to create targeted mutations in plants (Li et al. 2013, 2015; Wang et al. 2014; Woo et al. 2015; Zsögön et al. 2018). The present study used CRISPR/Cas9 to create targeted rearrangements in two plant disease resistance clusters to demonstrate accelerated generation of novel NLRs.

We selected two tandem duplicated NLR clusters in soybean. One cluster is low-copy and thus is tractable for genetic studies, while the other one is highly duplicated and complex carrying multiple paralogs. Asian soy rust (ASR) caused by *Phakopsora pachyrhizi* Syd. & P. Syd. poses a growing threat to global soybean production (Pivonia and Yang 2004). A region on the long arm of soy chromosome 18 harbors multiple closely linked resistance genes (Hossain et al. 2015; Kim et al. 2012; Liu et al. 2016; Yamanaka et al. 2016), which corresponds to approximately 2.5 Mbp in physical distance (http://www.soybase.org). We selected a 4-copy tandem duplicated NLR complex in this region as one of our models and refer to it as Rpp1L (Rpp1-like) throughout this paper.

The Rps1 locus on chromosome 3, unlike Rpp1L, is a complex NLR family with 22 tandem duplicated paralogs. Two independent paralogs within this gene cluster have been implicated with resistance against the oomycete pathogen, *Phytophthora sojae* Kaufm. & Gerd. (Gao et al. 2005; Shan et al. 2004; Zhang et al. 2013).

The present work demonstrates CRISPR/Cas9-mediated rearrangements of two NLRs gene complexes of soybean, Rps1 and Rpp1L, which thus provides the first evidence for accelerated diversification of the genes underlying immunity in plants using site-specific endonucleases.

## Materials and methods

### Target genes

**Fig. 1:**
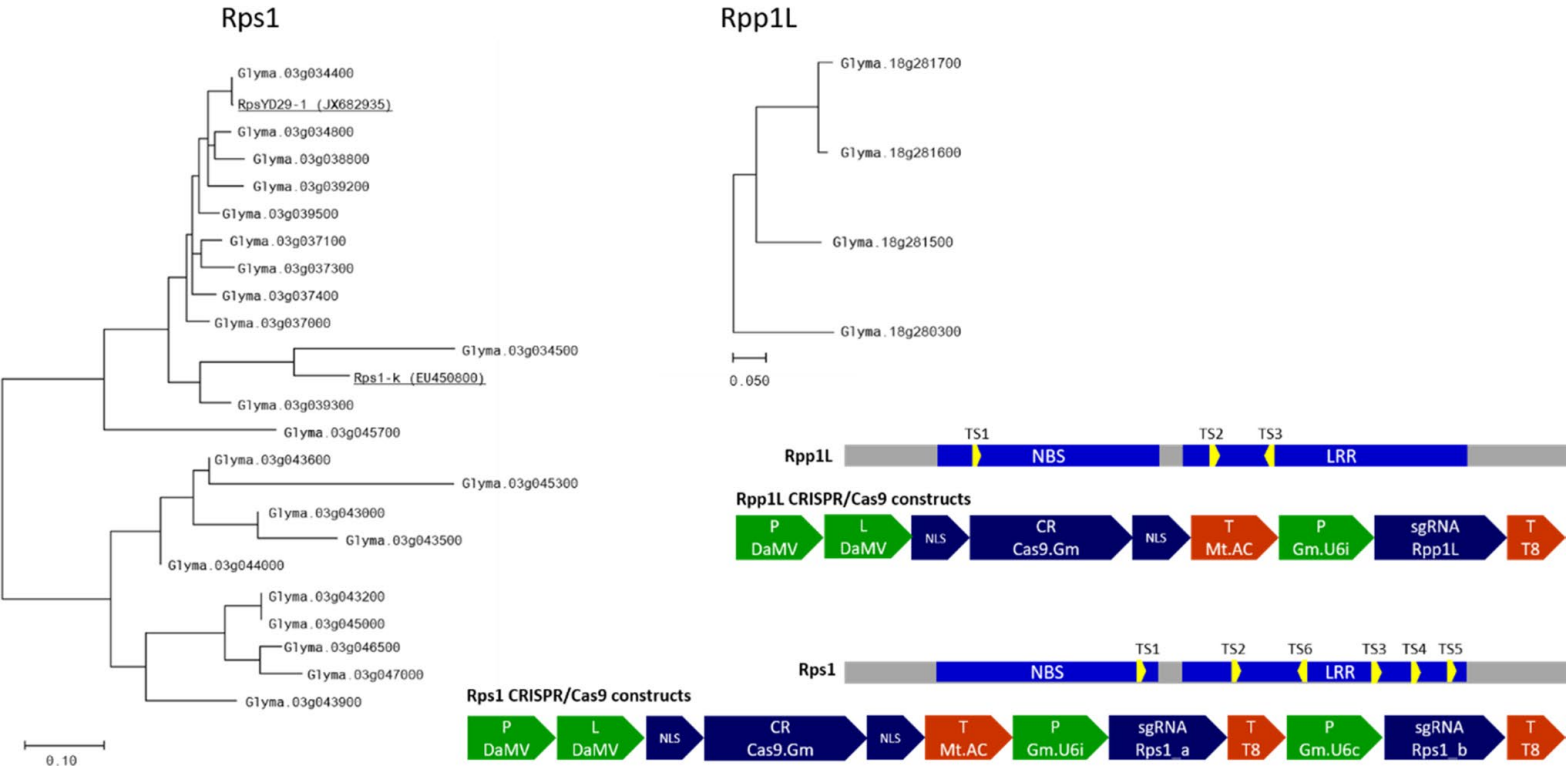
Maximum Likelihood (Tamura and Nei 1993) dendrograms of the Rps1 and Rpp1L (Rpp1-like) NLR clusters. Rps1 has 22, Rpp1L has 4 paralogs, as identified in Soybase (http://www.soybase.org) assembly Wm82.a2.v1. The two additional, underlined paralogs in Rps1, RpsYD29-1 and Rps1-k are published members of this gene cluster and have been associated with Phytophthora resistance (Gao et al. 2005; Zhang et al. 2013). The Rpp1L and Rps1 NBS-LRR domain structures of consensus sequences and the approximate locations of the CRISPR/Cas9 target sites (TS) are shown on two horizontal bars. The targeting constructs are also presented. The letter codes for the elements are as follows: promoter (P), nuclear localization signal (NLS), coding region (CR), terminator (T), non-coding single-guide RNA (sgRNA). The Rpp1L constructs carried one, while the Rps1 constructs carried two sgRNA cassettes targeting homologous sites

Multiple closely linked ASR resistance genes have been genetically mapped to a region between the markers Satt191 and Sat_372 on chromosome 18 (Hossain et al. 2015; Kim et al. 2012; Liu et al. 2016; Yamanaka et al. 2016), which corresponds to 2,577,642 bp in physical distance (http://www.soybase.org) in the reference genome Williams 82 (W82). A four-copy NLR family of this region has been selected (Fig. 1; Table S1a) as a model gene cluster and was named Rpp1-like (Rpp1L). Three of the paralogs (A, B and C) are closely linked and are in head-to-tail orientation, while the fourth one (D) is more distant and is in the opposite orientation.

The Rps1 cluster on chromosome 3 includes 22 paralogs (Fig. 1; Table S1b) and is homologous to the Rps1-k (GenBank accession, EU450800) (Gao et al. 2005) and RpsYD29-1 (GenBank accession, JX682935) (Zhang et al. 2013) genes associated with Phytophthora resistance.

All experiments were performed concomitantly in two soy germplasms, A3555 and AG3931, which were selected for their susceptibility to both ASR and Phytophthora. Since these genomes are not fully sequenced, all designs were based on the W82 reference genome.

Three CRISPR/Cas9 target sites were designed for the Rpp1L cluster (TS1, -2 and -3), which were conserved across at least the three closely linked paralogs (paralogs A, B and C in Tables S1a, S2a). Two sites (TS1 and TS2) were conserved across all four paralogs in the W82 reference genome. TS3 had a mismatch in the PAM-distal end in paralog D, which however was not expected to block cutting due to its position being distal to PAM. Target site 1 (TS1) was in the NBS domain, while the other two sites, TS2 and TS3 were in the more variable LRR domain (Fig. 1).

In the Rps1 cluster, six target sites were selected (TS1-TD6). TS1 was in the NBS domain, the other five sites were in the LRR domain (Fig. 1). None of the sites were conserved across all 22 paralogs. To maximize cutting frequencies, 2 homologous sgRNAs, each targeting multiple paralogs were inserted in each of the 6 Rps1 targeting constructs, which thus were potentially able to cut 12–18 out of the 22 paralogs each (Table S2b, Fig. S1).

### Molecular constructs

All molecular constructs included a Cas9 cassette, driven by the 35S promoter of Dahlia mosaic virus (DaMV) and at least one sgRNA cassette driven by the native U6i promoter of soy (Fig. 1). The six Rps1 constructs included a second sgRNA cassette too to target the secondary, homologous sites. These cassettes were driven by another native soy promoter, U6c. All relevant sequences of targeting constructs are listed in Supplemental Data S1.

### CRISPR/Cas9 activity in the R0 generation

A3555 and AG3931 embryos were transformed with CRISPR/Cas9 constructs using standard Agrobacterium-mediated transformation (Trick and Finer 1998). The primary transformed plants that were generated are called R0 transformants, their progenies constitute the R1 generation. Total genomic DNA was isolated from leaf punches of R0 transformants. The copy numbers of CRISPR/Cas9 constructs were determined by standard quantitative PCR (qPCR) using the Mt-AC140914v20 terminator as a template. PCR amplicons spanning the target sites were generated using primers (Table S3) labeled with FAM fluorophore. Twenty µl PCR mixture included 0.2 µl Phusion High-Fidelity Polymerase (M0530L, New Englad Biolabs, http://www.neb.com), 10 pmols of each of the primers, 100 µmol of each of the four nucleotides and 3 µl of R0 DNA solutions. For thermal cycling nine touch-down cycles were used with annealing temperatures gradually decreasing from 67 to 58 °C followed by 30 regular cycles at 58 °C annealing temperature. Amplicons were separated using the single-basepair resolution capillary electrophoresis platform of ABI3730 DNA Analyzer (http://www.thermofisher.com) according to the manufacturer’s instructions. Each plate included two samples from each of the parental genotypes to establish the wild-type peaks for comparison to the mutants. CRISPR/Cas9 mutants were called by the presence of non-parental alleles corresponding to targeted insertions or deletions (indels). To guard against the confounding effect of sporadic, low-intensity amplicons, we added a fluorescence amplitude criterion (> 100A) beyond the length variation criterion to the mutant call algorithm. Unlabeled amplicons from selected mutants were cloned into Zero Blunt TOPO PCR Cloning Kit (http://www.thermofisher.com). Randomly selected colonies were sequenced using Sanger method to verify targeted indels.

### Quantifying large-scale chromosome rearrangements in the R1 generation

Droplet-digital PCR (ddPCR) was used for high-throughput (HTP) analysis of copy number variants in the Rpp1L and Rps1 gene clusters. ddPCR was performed according to the manufacturer’s recommendation. 25 µl reaction mixture included ddPCR Supermix for Probes from Biorad (186-3025, http://www.bio-rad.com), 0.9 µM of each of the four primers, 0.25 µM of each of the test and reference probes, 10 μl genomic DNA and 20 U NdeI restriction enzyme. Cleavage by NdeI separated linked paralogs in either gene clusters thus assuring equal distribution of templates in droplet generation. The reactions were kept at room temperature for 15 min to allow restriction endonuclease activity prior to droplet generation. PCR included an initial denaturation step at 95 °C for 10 min, 40 cycles of denaturation at 94 °C for 30 s and annealing/extension at 59 °C for 1 min.

For assay validation, total genomic DNA was isolated from the two wild type germplasms, A3555 and AG3931 using DNeasy Plant Mini Kit (http://www.qiagen.com). Altogether seven TaqMan assays were designed for conserved exonic regions of the Rpp1L and Rps1 clusters (Table S4). Each assay was conserved across at least three paralogs in the W82 reference genome. First, all seven assays were tested across a genomic DNA concentration gradient ranging from 0.04 to 5 ng/μl in each transformation line. In the next validation step, the assays were tested for variation among four technical replicates. The DNA concentration and the TaqMan assays that performed the best were selected for large-scale screening of R1 transformants. The Rpp1L- and Rps1-specific assays were tested in combination with a reference assay from the aspartate aminotransferase gene of soy (AAT1; GenBank accession NM_001250612), which was shown to have a single-copy template in the soy genome in previous, unrelated studies. Gene copy numbers were calculated by normalizing the test concentrations (template copy/µl) by the control concentrations.

For each target site, 70–158 random R1 individuals were selected for copy-number variations (CNV) assay. Total genomic DNA was isolated from these plants using the high-throughput MagMax Technology (http://www.thermofisher.com). These R1 populations were tested side-by-side with 80–94 negative controls. The negative controls were randomly selected R1 transformants from the counterpart gene cluster. For example, for the populations targeted in the Rpp1L genes, Rps1-targeted R1 individuals were used as controls. The Rpp1L-specific CNV assay was used in both populations. Likewise, when the Rps1-targeted populations were tested for CNVs, a random subset of the Rpp1L-targeted populations was used as control. In this case, Rps1-specific assays were used for both tests and controls. Therefore, both tests and controls underwent the same procedure of transformation, tissue culture and plant regeneration and they carried very similar targeting constructs differing only in their sgRNAs cassettes.

All CNV data were analyzed using R Studio. First, outliers were removed using the inter-quantile range (IQR) method for each population. Data points that fell below Q1 minus 1.5 IQR or above Q3 plus 1.5 IQR, where Q1 is 25% quantile and Q3 is 75% quantile, were identified as outliers. The Kolmogorov–Smirnov (TS) test, which is a non-parametric test for pairwise comparison of continuous distributions was used to compare test and control populations. Differences in distributions were called significant below *p* = 0.05.

### DNA sequence analyses of R1 mutants

Primers were designed to amplify and sequence about 1 kb regions around each of the three target sites (Table S5). The three primer pairs were conserved across the A, B and C paralogs. Two of the six primers, the forward primers for the target regions 1 and 3 had an internal mismatch to paralog D, each, which however were not expected to block amplification completely. First, amplicons from the two parental genotypes were generated. PCR conditions were the same as shown above in the R0 population. The amplicons were separated in agarose gel, isolated and cloned using Zero Blunt TOPO PCR Cloning Kit (Thermo Fisher Scientific; http://www.thermofisher.com). Twenty-four random colonies per amplicon were sequenced in each genotype using Sanger method. GenBank accession numbers of parental sequences are as follows: MW178283-MW178294 (A3555) and MW178295-MW178306 (AG3931). Next, 24 Rpp1L mutants were selected from each of the A3555 and AG3931 R1 populations for re-sequencing. The ddPCR copy numbers of all selected mutants differed from the parental three copies by at least 0.5 copies. From each mutant at least 8, in a few cases up to 24 random colonies were sequenced to analyze their paralog configurations. One selected AG3931 R1 mutant failed to produce any high-quality sequence. Amplicon sequences were aligned and analyzed using ClustalW in the Mega-X software (Kumar et al. 2018).

Scarless, i.e., point mutation—free junctions among parental paralogs can theoretically emerge as amplification artifacts (Wang and Wang 1996). Inverse PCR was performed on three R1 mutants carrying scarless A/C chimeric junctions to confirm their genotypes. Low-concentration (~ 1 ng/μl) genomic DNA from the three mutants were concomitantly digested by the restriction endonucleases, AseI and NdeI (New Englad Biolabs; http://www.neb.com), both creating complementary 5′ TA overhangs. This generated approximately 3.1 kb-long fragments around the three Rpp1L target sites in either paralog A, C, or in their chimera. T4 DNA Ligase (New Englad Biolabs; http://www.neb.com) was used to circularize these fragments. Two primers conserved between paralogs A and C (CCATTGCTACCTCCGTTCAC and TTGCACTTCCCAATTTAACC), both facing outwards from the target sites were used to amplify the AseI/NdeI junctions. Amplicons were cloned using Zero Blunt TOPO PCR Cloning Kit and sequenced.

## Results

### Editing efficiency in the R0 generation

We selected Rpp1L, a relatively simple repetitive locus, and Rps1, a highly complex NLR cluster to study CRISPR/Cas9-mediated rearrangements (Fig. 1; Table S1). Three target sites in the Rpp1L cluster and six sites in the Rps1 clusters were targeted using CRISPR/Cas9 (Fig. 1; Table S2).

Forty-eight independent primary transformants (R0) were generated for each target site in each of the two transformation genotypes, A3555 and AG3931. Editing efficiencies were assessed by indel frequencies in the R0 plants (Fig. S2). The Rps1 target site (TS) 2 showed the lowest (0% in A3555), while Rpp1L TS2 the highest mutation rate (95.2% in A3555). Three target regions failed to produce scorable assays in either A3555 or AG3931. The average mutation rate for all Rps1 and Rpp1L sites was 39.1%. Amplicons were validated by sequencing at three randomly chosen target sites (Rpp1L-TS2, Rps1-TS4 and Rps1-TS5). In all cases, the indels responsible for the amplicon size variations fell within the Cas9 target sites (Fig. S2), thus confirming their CRISPR/Cas9-derived origin. R0 plants with one or two copies of stably integrated CRISPR/Cas9 constructs were grown to maturity and were advanced to R1 generation after self-pollination (Table S6).

### Quantifying gene copy number variation in the R1 generation

**Fig. 2:**
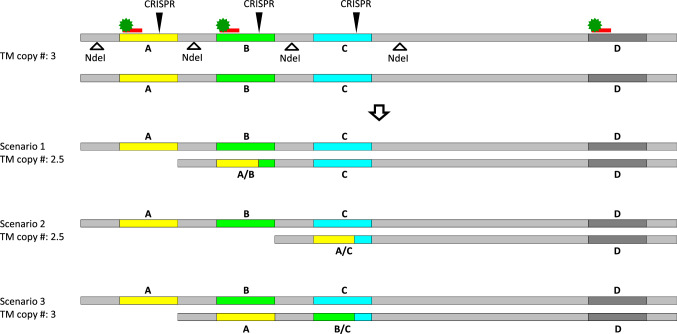
Schematic illustration of hypothetical, CRISPR/Cas9-mediated chromosomal rearrangements in the Rpp1L cluster. Chromosome cutting by CRISPR/Cas9 (black arrows) and subsequent DNA repair creates novel A/B, A/C or B/C chimeric paralogs in one of the parental chromosomes. The other chromosome remains intact. To detect these mutations by ddPCR, first, the genomic DNA is cleaved by the restriction endonuclease NdeI to physically separate the paralogs and then a TaqMan assay (red bar with a green star) conserved among three of the four paralogs of Rpp1L is deployed in ddPCR to detect copy number variations. ddPCR will detect three copies of Rpp1L in the parental genome. In the first and second mutant scenarios the detectable copy numbers will drop to 2.5, while it remains 3 in the third one

Copy number variation (CNV) detected by droplet digital PCR (ddPCR) was used to identify chromosomal rearrangements in the Rpp1L and Rps1 gene clusters. Figure 2 illustrates three hypothetical scenarios where NHEJ-mediated rearrangements generated novel paralogs in the Rpp1L cluster. In two of them, the rearrangements resulted in copy number variations detectable by ddPCR.

For the two gene clusters, altogether seven TaqMan assays were designed (Table S4). Their copy numbers were validated first in the two transformation lines, A3555 and AG3931. The detected copy numbers, as expected varied among the assays and genotypes, but were mostly consistent across a concentration gradient ranging from 5 to 0.04 ng/µl (Fig. S3a, b). The Rpp1L assay TM176/ TM177/TM32P was an exception with poor consistency across the concentration gradient and thus was omitted from the further validation steps. Some of the technical replicates of the high-copy number Rps1 assays were saturated at the highest, 5 ng/µl DNA concentrations, which thus yielded invalid results. 0.2 ng/µl concentration provided balanced distribution of positive and negative droplets across all assays, and thus was selected for high-throughput screening of the R1 populations. While all assays were highly consistent among technical replicates when measured at standard, 0.2 ng/µl DNA concentration (Fig. S3c), TM179/TM180/TM34P (Rpp1L) and TM181/TM182/TM35P (Rps1) showed the tightest droplet clustering based on visual judgement of the ddPCR output profiles. TM181/TM182/TM35P detected the highest copy numbers of Rps1 too. Therefore, these two assays were selected for HTP testing of the genome edited Rpp1L and Rps1 populations. TM179/TM180/TM34P was conserved in paralogs A, C and D of the W82 Rpp1L cluster, but mismatched in paralog B. TM181/TM182/TM35P was conserved across 6 of the 22 W82 Rps1 paralogs: A, D, E, F, H and J (Table S1).

**Fig. 3:**
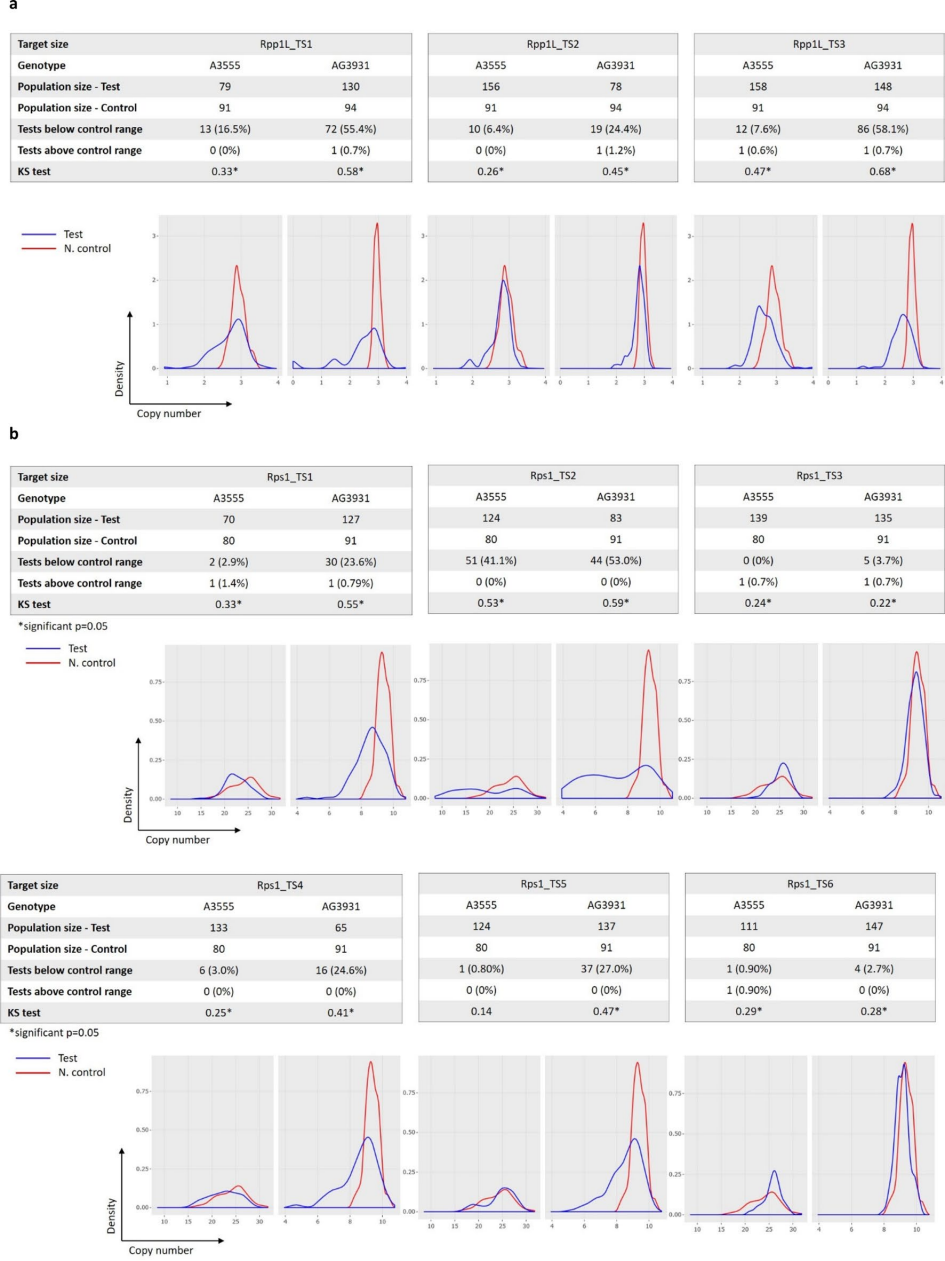
Copy number variations (CNVs) of the Rpp1L (**a**) and Rps1 (**b**) NLR clusters in R1 populations. CNV was used as a tool to screen for large-scale chromosomal rearrangements in the two gene clusters. The first approach to compare the CNV distributions between tests and controls, was to calculate the percentage of test transformants that fell below or above the control copy number distribution. Kolmogorov–Smirnov (KS) test was also used to compare the distributions statistically. Test populations labeled by asterisk were significantly different from their controls at *p* = 0.05. All test populations were significantly different from their controls except Rps1_TS5 in A3555

Distributions of CNVs in the R1 generation were plotted as density curves after removing outliers. The negative controls of the Rpp1L test populations showed a narrow distribution around three copies (Fig. 3a). CNV in most test populations significantly differed from those of negative controls. For the Rps1 gene, the detectable copy numbers were 26 and 9 in A3555 and AG3931, respectively, as judged by modes of the copy number density curves in the control populations. The distributions of CNVs in Rps1 were broader than for Rpp1L (Fig. 3b). Nonetheless, most Rpp1L and Rps1 test populations were significantly different from their negative controls at *p* = 0.05 as shown by the Kolmogorov–Smirnov (KS) test. The only exception was Rps1-TS5 (Fig. 3b), where the distribution of the test and control populations were statistically equivalent. Generally, CNV distributions in test populations shifted toward reduced copy numbers relative to controls suggesting that most NLR clusters lost paralogs during Cas9-mediated rearrangements. Even though the copy number of a few test samples was above their control ranges, the difference did not typically exceed one copy. Such small differences could have been caused by either true biological variations or technical limitations.

### Sequence-based confirmation of novel NLR paralogs

While the measurement of CNV is an efficient screening tool for large-scale chromosomal changes in NLR clusters, ultimate validation of novel paralogs requires sequencing. To avoid typical artifacts that can arise when amplifying in highly repetitive regions, special technologies are needed for targeted deep sequencing of the Rps1 cluster. Therefore, sequence analysis of the Rps1 mutants was out of scope for this study. On the other hand, Rpp1L represented a smaller and more tractable cluster amenable to sequence analysis.

**Fig. 4:**
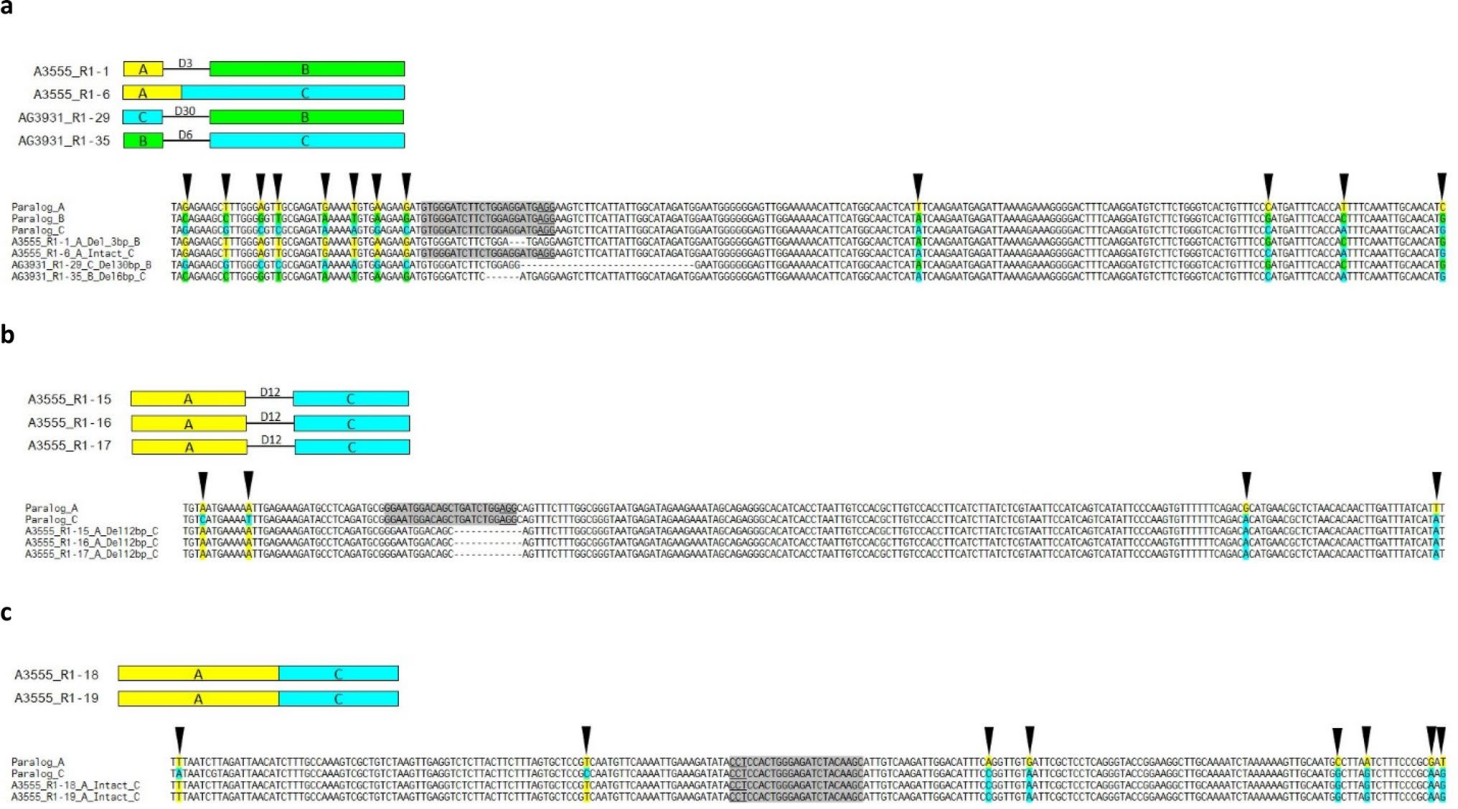
Annotated amplicon sequences of the novel, chimeric Rpp1L paralogs found in R1 CRISPR/Cas9 mutants at target site 1 (**a**), target site 2 (**b**) and target site 3 (**c**) and their alignment to the parental paralogs. Polymorphic nucleotides are color-coded according to their parental origins: paralog A, yellow; paralog B, green; paralog C, cyan. The target sites are shaded in gray, PAMs are underlined

For Rpp1L, 24 copy-number variant R1 progenies were selected from each of the A3555 and AG3931 populations for re-sequencing (Table S7). Primers conserved across paralogs were designed to amplify and sequence ~ 1 kb regions around the target sites. The parental sequences in both genotypes were identical with the corresponding regions of the W82 public reference genome. Targeted short indels were the most prevalent forms of mutations observed (Table S7; Supplemental Data S2). Except for one A3555 and another AG3931 genotype (R18 and R-25, respectively), all mutants had at least one paralog with indels in the CRISPR/Cas9 cut site. In 8 of the 24 A3555 mutants (R1-1, -4, -6, -15, -16, -17, -18 and -19) and 2 of the AG3931 mutants (R1-29 and -35) chimeras between the parental paralogs were identified. The breakpoint between the native paralogs in all these mutants co-localized with the target site. In three of these ten mutants, R1-6, -18 and -19, the chimeric paralogs did not have indels at the target site, i.e., the junctions between the parental paralogs were scarless (Table 1; Table S7). R1-18 and -19 were sister seeds from the same R0 family, so the new paralogs probably originated from a single recombination event in the R0 generation. In six other chimeric mutants, R1-1, R1-15, R1-16, R1-17, R1-29 and R1-35 the lengths of the indels were either three or multiples of three nucleotides and thus the original open reading frames were preserved (Table 1). Like R1-18 and -19, R1-15, R1-16 and R1-17 were also sister plants from the same R0 progenitor, which suggests that the chimeric paralog they shared was formed in the R0 generation too. These altogether nine mutants carrying in-frame chimeric paralogs demonstrate CRISPR/Cas9-mediated generation of novel combinations of parental NLR genes (Table 1). Alignments of the chimeric paralogs with their parental counterparts are shown in Fig. 4. To guard against misinterpretation of PCR artifacts in the scarless chimeric paralogs, we repeated the process of PCR, cloning and sequencing of eight randomly picked clones two additional times. The chimeras were detected in all three technical replicates for all three mutants. To further confirm the identities of these chimeras, inverse PCR (iPCR) was performed in all three mutants carrying scarless A/C paralogs (Fig. S4, Supplemental Data S3). In all three mutants, chimeric A/C paralogs were recovered, which was the ultimate proof for CRISPR/Cas9-mediated creation of novel paralogs with scarless chimeric junctions. The chimeras with indels at the Cas9 target sites (R1-1, R1-15, R1-16, R1-17, R1-29 and R1-35) cannot be generated by template switch between parental paralogs, and thus they did not need the same validation as the scarless chimeras.

**Table 1:** Novel, chimeric paralogs identified in R1 transformants that carried either intact, scarless junctions at the target site, or deletions that recovered the original open reading frames

Genotype	R1 mutant	R0 mutant	Target site	Copy number	Upstream region	Junction at TS	Downstream region
A3555	R1-1	R0-1	TS1	1.6	A	Del 3 bp	B
A3555	R1-6	R0-3	TS1	2	A	Intact	C
A3555	R1-15	R0-9	TS2	1.9	A	Del 12 bp	C
A3555	R1-16	R0-9	TS2	1.9	A	Del 12 bp	C
A3555	R1-17	R0-9	TS2	1.9	A	Del 12 bp	C
A3555	R1-18	R0-10	TS3	1.8	A	Intact	C
A3555	R1-19	R0-10	TS3	1.9	A	Intact	C
AG3931	R1-29	R0-18	TS1	2.1	C	Del 30 bp	B
AG3931	R1-35	R0-20	TS1	1.5	B	Del 6 bp	C

These novel paralogs encode for potentially altered disease resistance specificities. The regions upstream and downstream of the target sites were identified based on their homologies to the A, B, C or D paralogs of the W82 reference genome. The types of junctions at the target sites are shown as ‘Del’ for deletion or ‘Intact’, if the original target sites were recovered. Additional, out-of-frame chimeric and non-chimeric paralogs are listed in Table S7

## Discussion

Rearrangements in tandem duplicated NLR gene clusters is a major source of new disease resistance specificities against plant pathogens. The current study presents genomic evidence for induction of such rearrangements by targeting double-strand chromosome breaks in two NLR clusters of soy using CRISPR/Cas9.

Chromosomal rearrangements in NLRs, whether they occur through natural mutations or through genetic engineering have high potential agronomic value. Finding them in a systematic way, however has largely been hindered by the challenges these chromosomal regions present against developing trusted, high-throughput detection methods. The rearrangements can vary in size and position, which delimits their predictability, especially over long NLR clusters. Moreover, most PCR-based methods in duplicated gene families are prone to generating artifacts when incomplete amplicons switch templates from one amplification cycle to another. These artifacts can account for up to 30% of all amplicons and can significantly collapse or inflate the actual diversity (Haas et al. 2011; Wang and Wang 1996). Since many chromosomal rearrangements in NLRs are associated with copy number changes, technologies for CNV detection can be useful to monitor such mutations indirectly. Droplet digital PCR (ddPCR), is a novel CNV detection platform (Hindson et al. 2011) where the templates are separated into individual reaction compartments prior to amplification, which makes ddPCR essentially immune to typical template switch artifacts. Moreover, the broad dynamic range of detectible CNVs (Hindson et al. 2011) allows their monitoring in even large and complex gene families, like Rps1. This report is the first demonstration of ddPCR to detect variations in NLRs, which can have broad applications in plant breeding and biotechnology. Most HTP genotyping tools currently used in plant breeding rely on detecting simple heterologous features, such as single-nucleotide polymorphisms (SNPs). The ddPCR-based assay platform demonstrated in this paper screens for polymorphisms at a higher level of genome complexity, which has not been formerly attainable in HTP manner. This assay can be instrumental in monitoring repetitive gene families in natural or, as in the current study, in genome edited populations.

In the R0 population, we quantified CRISPR/Cas9 activities by counting targeted indels. Amplicons generated by regular PCR were first electrophoretically analyzed in a quantitative manner, which was followed by sequence-based, qualitative confirmation of Cas9 activities. Most of the nine targeting constructs yielded substantial, up to 95.2% indel rates in both genotypes. On the other hand, there were three sites that failed to produce scorable amplicons in either of the two genotypes. While capillary electrophoresis is a trusted method to quantify indels for single-copy loci, in complex, duplicated regions, like Rpp1L and Rps1, it may not be completely immune to PCR artifacts described above. This might have confounded the quantitative data at some extent in the R0 populations. Despite these potential caveats, capillary electrophoresis is a simple and fast screening tool that helped accelerate the more laborious amplicon sequencing afterwards. Sequence-based confirmation of targeted indels was the ultimate evidence for CRISPR/Cas9 activities in the R0 population, which thus helped advancing experimentation to the next phase.

The R1 population has gone through multiple mitotic and one meiotic division post transformation, which created ample opportunities for Cas9-mediated re-shuffling of parental paralogs. Therefore, we studied chromosomal rearrangements in the R1 populations. We detected 26 and 9 copies of the Rps1 gene complex in A3555 and AG3931, respectively; and 3 copies for Rpp1L in both genotypes. The CNV histograms of Rps1 had broader distributions than those of Rpp1L even in the negative control populations. This broader variation in Rps1 may have been caused by sequence variations in the assay templates. As judged by the W82 genome, only a fraction of all paralogs (6 out of 22) had perfect matches to the TM assay used for Rps1. However, many others carried polymorphisms in positions that may not have completely blocked, only suppressed amplifications at variable extent. This may have been manifested in inconsistent patterns of amplifications, which thus artificially expanded the CNV distributions in the Rps1 populations.

A significant fraction (up to 58.8%) of most R1 populations differed in copy numbers from their control populations suggesting CRISPR/Cas9-mediated chromosomal rearrangements. The number of paralogs typically decreased in the mutants, which suggests that the predominant mechanism of rearrangements was large intra-chromosomal deletion during DSB repair. Using target sites that are less conserved among paralogs would delimit the number of concurrent DSBs, which may lead to different copy number distributions. Using meiosis-specific promoters for driving expression of the CRISPR reagents could enhance the rate of unequal recombination versus deletions, which, would also lead to alternative distributions of copy number variants.

To confirm emergence of novel chimeric paralogs, amplicons spanning the CRISPR/Cas9 target sites were sequenced in 47 Rpp1L mutants from 2 inbred-derived R1 populations. Nine of them carried Rpp1L paralogs that represented novel combinations of the parental counterparts while preserving the original open reading frames. If Rpp1L is responsible for Asian Soy Rust resistance, these nine new mutants could confer new disease resistance specificities. Beyond developing novel resistance traits, mutants can also be used to identify causal genes for disease resistance. We did not aim to exhaustively catalog all paralogs that arose in these mutants, rather to identify the most abundant ones that would likely transmit to subsequent generations. As a result, some paralogs may have remained undetected by our sequencing assay.

Most sequenced Rpp1L paralogs carried targeted indels suggesting that DSBs were repaired predominantly by NHEJ. On the other hand, the chimeric paralogs with scarless junctions could have been repaired by either NHEJ or by an alternative mechanism, such as homology-dependent unequal recombination or single-strand annealing. Some novel paralogs have apparently undergone multiple cycles of DNA cleavage and repair. For example, R1-18 and -19, which were descendants of the same primary transformant R0-10, carried the same A/C paralog with intact target sites in the middle. However, R1-19, in addition to the scarless chimera included A/C paralogs with a 1 bp insertion and an 8 bp deletion too, which were absent in R-18. This suggests that the initial A/C chimerization occurred in the R0 progenitor, which was then further mutated by secondary cuts only in R-19. The near-identical copy numbers between R1-18 and -19 suggests that these two additional A/C paralogs in R-19 were not created by duplication, rather by mosaic DNA cleavage and repair among various tissue segments. We identified two C/B chimeric configurations in R-25, where the order of the participating paralogs was opposite to the original one. This kind of chimerism is best explained by inter-chromosomal as opposed to intrachromosomal recombination. Therefore, these chimera likely represent CRISPR/Cas9-induced unequal recombinations between parental chromosomes.

While paralog D of Rpp1L was represented among the sequenced amplicons in the R1 generation, it was not involved in any of the ten chimera identified. The sequencing method used was not able to detect head-to-head chimerisms, the ones that would most likely emerge from oppositely oriented genes.

Copy numbers detected by ddPCR did not always match the number of sequenced paralogs. As mentioned above, sequencing was shallow, which did not guarantee representation of all paralogs. Furthermore, only three of the four Rpp1L paralogs were detectable by ddPCR, while all four paralogs were detected by sequencing. Thirdly, as demonstrated by the R1-19 mutant above, mosaicism in the tissue samples sometimes led to detection of more polymorphic paralogs by sequencing than revealed by ddPCR.

The present study is the first published record of engineering NLR gene clusters to diversify disease resistance loci in crops. Novel high-throughput detection methods for chromosomal rearrangements combined with DNA sequencing identified new mutants of interest in large populations and helped to describe rearrangements in these complex genomic regions. The concept demonstrated in this study is readily applicable to any selected NLR cluster of interest in crop species amenable to genetic transformation.

## Supplementary Information

Below is the link to the electronic supplementary material.

Supplementary file1 (DOCX 1375 KB)

## Data Availability

All novel parental sequence data generated in this study have been submitted to NCBI GenBank. Accession numbers are MW178283-MW178306.
