# Supplementary material for: Novel disease resistance gene paralogs created by CRISPR/Cas9 in soy
Source: Plant Cell Rep. 2021 Mar 11;40(6):1047–58. doi: 10.1007/s00299-021-02678-5 (PMC8184530; doi:10.1007/s00299-021-02678-5)
Supplement: Supplementary file 1 — Supplementary file1 (DOCX 1375 KB) [file 299_2021_2678_MOESM1_ESM.docx]

**Supplementary Material**

**Table of contents**

Table S1................................................2

Table S2................................................3

Table S3................................................4

Table S4................................................5

Table S5................................................6

Table S6................................................7

Table S7................................................8

Figure S1..............................................12

Figure S2..............................................13

Figure S3..............................................14

Figure S4..............................................16

Supplementary Data S1..................................17

Supplementary Data S2..................................19

Supplementary Data S3..................................36

**Table S1** Nomenclature and coordinates of the paralogs in the Rpp1L (**a)** and Rps1 (**b)** gene clusters on chromosomes 18 and 3, respectively as shown in SoyBase (www.soybase.org) assembly Wm82.a2.v1.

**a**

| **Rpp1L Paralog** | **Name** | **Coordinates** | **Orientation** |
| --- | --- | --- | --- |
| A | Glyma.18g281700 | 56264735..56272703 | - |
| B | Glyma.18g281600 | 56245668..56252626 | - |
| C | Glyma.18g281500 | 56234929..56241066 | - |
| D | Glyma.18g280300 | 56110850..56121368 | + |

**b**

| **Rps1 Paralog** | **Name** | **Coordinates** | **Orientation** |
| --- | --- | --- | --- |
| A | Glyma.03g034400 | 4026212..4030630 | + |
| B | Glyma.03g034500 | 4039194..4057458 | - |
| C | Glyma.03g034800 | 4122756..4127065 | - |
| D | Glyma.03g037000 | 4515956..4520577 | + |
| E | Glyma.03g037100 | 4541759..4546288 | + |
| F | Glyma.03g037300 | 4566741..4571115 | + |
| G | Glyma.03g037400 | 4593854..4598176 | + |
| H | Glyma.03g038800 | 4790555..4795288 | + |
| I | Glyma.03g039200 | 4852112..4856672 | + |
| J | Glyma.03g039300 | 4890564..4894600 | + |
| K | Glyma.03g039500 | 4913380..4917059 | + |
| L | Glyma.03g043000 | 5442063..5445923 | + |
| M | Glyma.03g043200 | 5457034..5460630 | + |
| N | Glyma.03g043500 | 5503203..5511390 | + |
| O | Glyma.03g043600 | 5517406..5521091 | + |
| P | Glyma.03g043900 | 5534982..5539691 | + |
| Q | Glyma.03g044000 | 5569654..5573380 | + |
| R | Glyma.03g045000 | 5717526..5721133 | - |
| S | Glyma.03g045300 | 5736880..5746061 | - |
| T | Glyma.03g045700 | 5815273..5822250 | - |
| U | Glyma.03g046500 | 5937077..5940658 | + |
| V | Glyma.03g047000 | 5976506..5982926 | + |

**Table S2** Nine CRISPR/Cas9 targeting constructs and their associated target sites in the Rpp1L (**a**) and Rps1 (**b**) gene clusters. The number of paralogs in the reference, W82 genome carrying the target site is shown in the last column. While the Rpp1L constructs had only one sgRNA cassette each, the Rps1 constructs included two sgRNA cassettes targeting two homologous sites.

**a**

| **Construct** | **Target Site** | **Target Sequence** | **Domain** | **Matching paralogs (total = 4)** |
| --- | --- | --- | --- | --- |
| pRpp1L_1 | Rpp1L_TS1 | GTGGGATCTTCTGGAGGATG_AGG | NBS | 4 (A, B, C, D) |
| pRpp1L_2 | Rpp1L_TS2 | GGAATGGACAGCTGATCTGG_AGG | LRR | 4 (A, B, C, D) |
| pRpp1L_3 | Rpp1L_TS3 | GCTTGTAGATCTCCCAGTGG_AGG | LRR | 3 (A, B, C) |

**b**

| **Construct** | **Target Site** | **Target Sequence** | **Domain** | **Matching paralogs (total = 22)** |
| --- | --- | --- | --- | --- |
| pRps1_1 | Rps1_TS1a | GATCTAGCCACATCACTCGG_TGG | NBS | 7 (A, B, C, G, H, J, K) |
|  | Rps1_TS1b | GATCTAGCATTATACCTTGG_TGG |  | 7 (L, N, O, Q, S, U, V) |
| pRps1_2 | Rps1_TS2a | CCAAAGTGATGAAGCGTTGG_AGG | LRR | 8 (A, C, D, F, G, H, J, K) |
|  | Rps1_TS2b | CAGAAGCAATGAAGCATTGG_AGG |  | 7 (L, N, O, Q, S, U, V) |
| pRps1_3 | Rps1_TS3a | GGAATATCTTTTGGTTTCAG_GGG | LRR | 6 (A, D, E, H, J, K) |
|  | Rps1_TS3b | GGAATCTCTTTTGGTTTCAG_GGG |  | 6 (C, L, N, O, Q, U) |
| pRps1_4 | Rps1_TS4a | ATTGAGTCCTTTCCAAAACG_GGG | LRR | 4 (A, D, H, I) |
|  | Rps1_TS4b | ATTGAGTCGTTTCCAGAAGG_GGG |  | 9 (F, G, J, L, N, O, Q, U, V) |
| pRps1_5 | Rps1_TS5a | TGGAGATGTTGGACTGCACA_GGG | LRR | 1 (A) |
|  | Rps1_TS5b | CTGGAGATGTTGGACTGCACG_GGG |  | 11 (C, D, F, G, I, K, L, N, P, Q, V) |
| pRps1_6 | Rps1_TS6a | GCAAACTTCCCTCTAGTTTG_GGG | LRR | 10 (A, C, D, E, F, G, H, I, J, K) |
|  | Rps1_TS6b | GCAAATCTCCCCTTAGTTTG_GGG |  | 8 (L, M, N, O, Q, R, U, V) |

**Table S3** Primers to amplify regions flanking the target loci in Rpp1L (**a**) and Rps1 (**b**) gene clusters when analyzing indels in the R0 generations. Amplicons were separated in capillary electrophoresis to identify length-variants deriving from targeted point mutations. Some Rps1 primers included degenerate nucleotides to enable amplifications from multiple paralogs.

**a**

| **Target Locus** | **Primer1** | **Primer2** |
| --- | --- | --- |
| Rpp1L_TS1 | GGATAAACTAAAGGACCTGA | GAAACAGTGACCCAGAAGAC |
| Rpp1L_TS2 | TGACAGATTGGAAATGGGAG | AGAGACCTCAACTTAGACAG |
| Rpp1L_TS3 | TTTGGCGGGTAATGAGATAG | GTCATCCCTTTTACATCTTC |

**b**

| **Target Locus** | **Primer1** | **Primer2** |
| --- | --- | --- |
| Rps1_TS1 | ATGAGTATTTTGATGATTTGG | CATTATGATACATTGTGCCTCC |
| Rps1_TS2 | ATGGAATCAAAGAATTGGGAGC | GTGAGGCTGTAACTTGCAAAGC |
| Rps1_TS3 | ACWAACATCCAACCAACTTGTC | TTGGGAGAAGASTACTCATCTC |
| Rps1_TS4 | ACWAACATCCAACCAACTTGTC | TCCAGCAAAGGACATCCCCTC |
| Rps1_TS5 | ACWAACATCCAACCAACTTGTC | TCCAGCAAAGGACATCCCCTC |
| Rps1_TS6 | ATTCAGAAGCTTTTCCTGTG | TTGGGAGAGAAGAGMCAAGC |

**Table S4** TaqMan assays tested for copy number variations in the Rpp1L and Rps1 gene clusters and a reference assay designed for the aspartate aminotransferase (AAT1) gene. Assays ‘Test3’ and ‘Test4’ along with ‘Reference’ (written in bold) were selected after the validation process for high-throughput screening of R1 transformants.

| **TM assay** | **Name** | **Sequence** | **Template** |
| --- | --- | --- | --- |
| Test1 | TM176-primer | TGGCTCGAACCATGAAAGG | Rpp1L |
|  | TM32P-probe | FAM-TGAGATCCATTGGTGGAGACATGCA-IowaBlack | Rpp1L |
|  | TM177-primer | CCTCTTCTCCCATTTCCAATCT | Rpp1L |
| Test2 | TM178-primer | TAGAAGAAATAGCAGAGGGCAC | Rpp1L |
|  | TM33P-probe | FAM-ATTGTCCACGCTTGTCCACCTTCA-IowaBlack | Rpp1L |
|  | TM180-primer | CACTTGGGAATATGACTGATGGA | Rpp1L |
| **Test3** | **TM179-primer** | **AGAAATAGCAGAGGGCACATC** | **Rpp1L** |
|  | **TM34P-probe** | **FAM-CGCTTGTCCACCTTCATCTTATCTCGT-IowaBlack** | **Rpp1L** |
|  | **TM180-primer** | **CACTTGGGAATATGACTGATGGA** | **Rpp1L** |
| **Test4** | **TM181-primer** | **GCTTGATGATGCCGAGAAGA** | **Rps1** |
|  | **TM35P-probe** | **FAM-CACAAACACCAATGTCAAACACTGGCT-IowaBlack** | **Rps1** |
|  | **TM182-primer** | **CGAGTAAGTCATCGGCTTCATAG** | **Rps1** |
| Test5 | TM183-primer | AAAGTTGGAGACCACTCTCAG | Rps1 |
|  | TM36P-probe | FAM-AGTGGTTGGAGCTGTGCTTGATGA-IowaBlack | Rps1 |
|  | TM184-primer | GAGATCATTGAGCCAGTGTTTG | Rps1 |
| Test6 | TM185-primer | AACGGAATCAAAGAACTGGGGAC | Rps1 |
|  | TM37P-probe | FAM-AGCGAACCATGAAGATTTGAAAG-IowaBlack | Rps1 |
|  | TM186-primer | TCATTGCTTYTGGTTACATTCTCC | Rps1 |
| Test7 | TM187-primer | TCAAATCTTCATGGTTCGCTTTC | Rps1 |
|  | TM38P-probe | FAM-TGCTTCTGGTTACATTCTCCAAATTCC-IowaBlack | Rps1 |
|  | TM188-primer | CATCCTAGCCTCCAATGCTTCAT | Rps1 |
| **Ref.** | **SQ549-primer** | **CGGAGATGCTGTCGTGAGAA** | **AAT1** |
|  | **PB0004-probe** | **VIC-ACTCTCGCTAGACACTTCCTTCCGCAAA-MGB** | **AAT1** |
|  | **SQ546-primer** | **CCCTGTGATCGCAGACTCAA** | **AAT1** |

**Table S5** Primers used to amplify regions flanking the target loci in Rpp1L gene clusters to analyze junctions of novel paralogs at each of the three target sites. Each amplicon is approximately 1kb in length and include multiple diagnostic SNPs on each side of the target sites to discriminate paralogs.

| **Target Locus** | **Primer1** | **Primer2** |
| --- | --- | --- |
| Rpp1L_TS1 | AAGTTGTTAGTGCTTAACATTC | GTCATCTCATCTCCGTAAAG |
| Rpp1L_TS2 | GGAAAAATGAGATCCATTGG | GTCATCCCTTTTACATCTTC |
| Rpp1L_TS3 | ATGGGAGAAGAGGTCTTAAG | TGAGGTTTTGGATATTAGTG |

**Table S6** R0 transformants carrying one or two copies of the CRISPR/Cas9 constructs and their R1 progenies generated. R0 transformants with alternative transgene copy numbers were discarded.

**a**

| **Target Locus** | **A3555** | | **AG3931** | |
| --- | --- | --- | --- | --- |
|  | **R0** | **R1** | **R0** | **R1** |
| Rpp1L_TS1 | 9 | 894 | 14 | 1512 |
| Rpp1L_TS2 | 16 | 1259 | 10 | 864 |
| Rpp1L_TS3 | 17 | 1849 | 17 | 1406 |
| Total | 42 | 4002 | 41 | 3782 |

**b**

| **Target Locus** | **A3555** | | **AG3931** | |
| --- | --- | --- | --- | --- |
|  | **R0** | **R1** | **R0** | **R1** |
| Rps1_TS1 | 16 | 1684 | 17 | 1317 |
| Rps1_TS2 | 16 | 1976 | 10 | 862 |
| Rps1_TS3 | 17 | 2099 | 16 | 1385 |
| Rps1_TS4 | 17 | 1852 | 10 | 593 |
| Rps1_TS5 | 15 | 1683 | 17 | 1673 |
| Rps1_TS6 | 13 | 1432 | 17 | 1575 |
| Total | 94 | 10726 | 87 | 7405 |

**Table S7** Paralogs identified in R1 transformants derived from the A3555 (**a**) and AG3931 (**b**) germplasms. The regions upstream and downstream of the target sites were identified based on their homologies to the A, B, C or D paralogs of the W82 reference genome. CRISPR/Cas9 mediated point mutations in the target sites are shown as ‘Del’, ‘Ins’ or ‘Sub’ denoting deletions, insertions or substitutions, respectively. ‘Intact’ target sites were wild type sequences with no point mutations. All mutant paralogs that underwent chimerization of the parental paralogs, and, at the same time, preserved the original open reading frames are marked in the last column.

**a**

| **R1 mutant** | **R0 mutant** | **Target**  **site (TS)** | **Copy number** | **Upstream**  **region** | **Mutation at TS** | **Downstream**  **region** | **Novel chimeric paralog in frame** |
| --- | --- | --- | --- | --- | --- | --- | --- |
| R1-1 | R0-1 | TS1 | 1.6 | A | Ins 1bp | B |  |
|  |  |  |  | A | Del 2bp | B |  |
|  |  |  |  | A | Del 3bp | B | ✓ |
|  |  |  |  | B | Del 3bp | B |  |
|  |  |  |  | B | Del 6bp | B |  |
|  |  |  |  | D | Del 4bp | D |  |
| R1-2 | R0-1 | TS1 | 2.0 | B | Sub 3bp | B |  |
|  |  |  |  | B | Del 1bp | B |  |
|  |  |  |  | B | Del 2bp | B |  |
|  |  |  |  | B | Del 22bp | B |  |
|  |  |  |  | B | Del 89bp | B |  |
|  |  |  |  | D | Del 21bp | D |  |
| R1-3 | R0-2 | TS1 | 2.0 | C | Del 2bp | C |  |
| R1-4 | R0-2 | TS1 | 2.0 | A | Del 71bp | B |  |
|  |  |  |  | C | Del 2bp | C |  |
| R1-5 | R0-2 | TS1 | 2.1 | A | Del 3bp | A |  |
|  |  |  |  | C | Del 19bp | C |  |
| R1-6 | R0-3 | TS1 | 2.0 | A | Intact | A |  |
|  |  |  |  | A | Intact | C | ✓ |
|  |  |  |  | C | Del 6bp | C |  |
| R1-7 | R0-3 | TS1 | 0.9 | A | Intact | A |  |
|  |  |  |  | A | Del 1bp | A |  |
|  |  |  |  | B | Intact | B |  |
|  |  |  |  | C | Del 6bp | C |  |
| R1-8 | R0-4 | TS1 | 3.6 | A | Intact | A |  |
|  |  |  |  | A | Del 2bp | A |  |
| R1-9 | R0-5 | TS2 | 2.0 | B | Del 1bp | B |  |
|  |  |  |  | B | Del 3bp | B |  |
|  |  |  |  | B | Del 67bp | B |  |
|  |  |  |  | C | Del 1bp | C |  |
| R1-10 | R0-5 | TS2 | 1.8 | B | Del 1bp | B |  |
|  |  |  |  | B | Del 2bp | B |  |
|  |  |  |  | B | Del 7bp | B |  |
|  |  |  |  | B | Del 10bp | B |  |
|  |  |  |  | B | Del 34bp | B |  |
| R1-11 | R0-5 | TS2 | 2.0 | B | Intact | B |  |
|  |  |  |  | B | Del 8bp | B |  |
|  |  |  |  | C | Intact | C |  |
|  |  |  |  | C | Del 1bp | C |  |
| R1-12 | R0-6 | TS2 | 2.1 | A | Del 8bp | A |  |
|  |  |  |  | B | Del 1bp | B |  |
|  |  |  |  | B | Del 4bp | B |  |
|  |  |  |  | B | Del 8bp | B |  |
|  |  |  |  | C | Del 37bp | C |  |
| R1-13 | R0-7 | TS2 | 3.4 | A | Intact | A |  |
|  |  |  |  | A | Del 2bp | A |  |
| R1-14 | R0-8 | TS2 | 1.9 | A | Del 6bp | A |  |
| R1-15 | R0-9 | TS2 | 1.9 | A | Del 12bp | C | ✓ |
|  |  |  |  | A | Del 12bp Ins 52bp | C |  |
|  |  |  |  | D | Intact | D |  |
| R1-16 | R0-9 | TS2 | 1.9 | A | Del 11bp Ins 25bp | C |  |
|  |  |  |  | A | Del 12bp | C | ✓ |
|  |  |  |  | D | Del 2bp | D |  |
| R1-17 | R0-9 | TS2 | 1.9 | A | Del 12bp | C | ✓ |
| R1-18 | R0-10 | TS3 | 1.8 | A | Intact | C | ✓ |
|  |  |  |  | D | Intact | D |  |
| R1-19 | R0-10 | TS3 | 1.9 | A | Intact | C | ✓ |
|  |  |  |  | A | Ins 1bp | C |  |
|  |  |  |  | A | Del 8bp | C |  |
|  |  |  |  | B | Del 7bp | B |  |
| R1-20 | R0-11 | TS3 | 1.9 | C | Del 2bp | C |  |
|  |  |  |  | C | Del 5bp | C |  |
|  |  |  |  | D | Del 2bp | D |  |
|  |  |  |  | D | Del 3bp | D |  |
| R1-21 | R0-12 | TS3 | 3.6 | A | Ins 1bp | A |  |
|  |  |  |  | B | Del 3bp | B |  |
|  |  |  |  | B | Del 7bp | B |  |
|  |  |  |  | B | Del 8bp | B |  |
|  |  |  |  | B | Del 13bp | B |  |
|  |  |  |  | D | Ins 1bp | D |  |
| R1-22 | R0-12 | TS3 | 4.0 | B | Del 4bp | B |  |
|  |  |  |  | B | Del 51bp | B |  |
|  |  |  |  | C | Del 5bp | C |  |
|  |  |  |  | D | Ins 1bp | D |  |
|  |  |  |  | D | Del 5bp | D |  |
| R1-23 | R0-13 | TS3 | 1.9 | A | Ins 1bp | A |  |
|  |  |  |  | A | Del 1bp | A |  |
|  |  |  |  | A | Del 12bp | A |  |
|  |  |  |  | C | Ins 1bp | C |  |
| R1-24 | R0-14 | TS3 | 1.8 | A | Del 4bp | A |  |
|  |  |  |  | A | Del 8bp | A |  |
|  |  |  |  | B | Del 2bp | B |  |
|  |  |  |  | C | Ins 1bp | C |  |
|  |  |  |  | D | Ins 1bp | D |  |
|  |  |  |  | D | Del 3bp | D |  |
|  |  |  |  | D | Del 12bp | D |  |

**b**

| **R1 mutant** | **R0 mutant** | **Target**  **site (TS)** | **Copy number** | **Upstream**  **region** | **Mutation at TS** | **Downstream**  **region** | **Novel chimeric paralog in frame** |
| --- | --- | --- | --- | --- | --- | --- | --- |
| R1-25 | R0-15 | TS1 | 1.8 | B | Intact | B |  |
|  |  |  |  | C | Intact | C |  |
| R1-26 | R0-16 | TS1 | 4.0 | A | Del 1bp | A |  |
|  |  |  |  | C | Del 6bp | C |  |
| R1-27 | R0-17 | TS1 | 1.6 | A | Del 1bp | A |  |
|  |  |  |  | A | Del 3bp | A |  |
|  |  |  |  | B | Del 1bp | B |  |
|  |  |  |  | B | Del 3bp | B |  |
|  |  |  |  | C | Del 2bp | C |  |
|  |  |  |  | C | Del 3bp | C |  |
| R1-28 | R0-18 | TS1 | 2.0 | A | Del 1bp | A |  |
|  |  |  |  | A | Del 2bp | A |  |
| R1-29 | R0-18 | TS1 | 2.1 | A | Del 1bp | A |  |
|  |  |  |  | A | Del 3bp | A |  |
|  |  |  |  | A | Del 4bp | A |  |
|  |  |  |  | C | Ins 1bp | B |  |
|  |  |  |  | C | Del 30bp | B | ✓ |
| R1-30 | R0-19 | TS1 | 1.5 | B | Del 3bp | B |  |
|  |  |  |  | C | Del 4bp | C |  |
| R1-31 | R0-19 | TS1 | 1.4 | A | Del 1bp | A |  |
|  |  |  |  | C | Del 4bp | C |  |
| R1-32 | R0-19 | TS1 | 1.4 | A | Del 1bp | A |  |
|  |  |  |  | C | Del 4bp | C |  |
|  |  |  |  | D | Intact | D |  |
| R1-33 | R0-19 | TS1 | 1.5 | B | Intact | B |  |
|  |  |  |  | C | Intact | C |  |
|  |  |  |  | C | Del 4bp | C |  |
|  |  |  |  | D | Del 17bp | D |  |
| R1-34 | R0-20 | TS1 | 1.8 | A | Del 1bp | A |  |
|  |  |  |  | B | Ins 1bp | B |  |
|  |  |  |  | B | Ins 26bp | B |  |
|  |  |  |  | D | Del 1bp | D |  |
|  |  |  |  | D | Del 6bp | D |  |
| R1-35 | R0-20 | TS1 | 1.5 | A | Ins 1bp | A |  |
|  |  |  |  | B | Del 1bp | C |  |
|  |  |  |  | B | Del 6bp | C | ✓ |
|  |  |  |  | B | Ins 19bp | C |  |
| R1-36 | R0-20 | TS1 | 1.5 | A | Del 1bp | A |  |
| R1-37 | R0-21 | TS1 | 1.5 | A | Del 3bp | A |  |
| R1-38 | R0-21 | TS1 | 1.4 | A | Del 3bp | A |  |
| R1-39 | R0-21 | TS1 | 1.5 | A | Del 2bp | A |  |
| R1-40 | R0-22 | TS2 | 2.1 | A | Del 6bp | A |  |
|  |  |  |  | B | Intact | B |  |
|  |  |  |  | D | Intact | D |  |
| R1-41 | R0-22 | TS2 | 1.9 | A | Del 6bp | A |  |
|  |  |  |  | B | Del 2bp | B |  |
|  |  |  |  | D | Intact | D |  |
| R1-42 | R0-23 | TS2 | 3.7 | B | Del 7bp | B |  |
|  |  |  |  | C | Del 1bp | C |  |
|  |  |  |  | C | Del 2bp | C |  |
| R1-43 | R0-24 | TS3 | 1.8 | A | Ins 1bp | A |  |
|  |  |  |  | C | Del 1bp | C |  |
| R1-44 | R0-24 | TS3 | 1.6 | A | Ins 1bp | A |  |
|  |  |  |  | C | Del 7bp | C |  |
| R1-45 | R0-25 | TS3 | 1.2 | B | Intact | B |  |
|  |  |  |  | B | Del 2bp | B |  |
|  |  |  |  | B | Del 4bp | B |  |
| R1-46 | R0-25 | TS3 | 1.2 | B | Intact | B |  |
|  |  |  |  | B | Ins 1bp | B |  |
|  |  |  |  | B | Del 1bp | B |  |
|  |  |  |  | D | Del 8bp | D |  |
| R1-47 | R0-25 | TS3 | 2.1 | B | Intact | B |  |
|  |  |  |  | B | Del 8bp | B |  |

**Fig. S1** Polymorphism at the Rps1 CRISPR/Cas9 target site TS2 among the 22 paralogs of the W82 reference genome. The two most conserved variants shown in blue (Rps1_TS2a) and red (Rps1_TS2b) were targeted by two separate sgRNA cassettes.


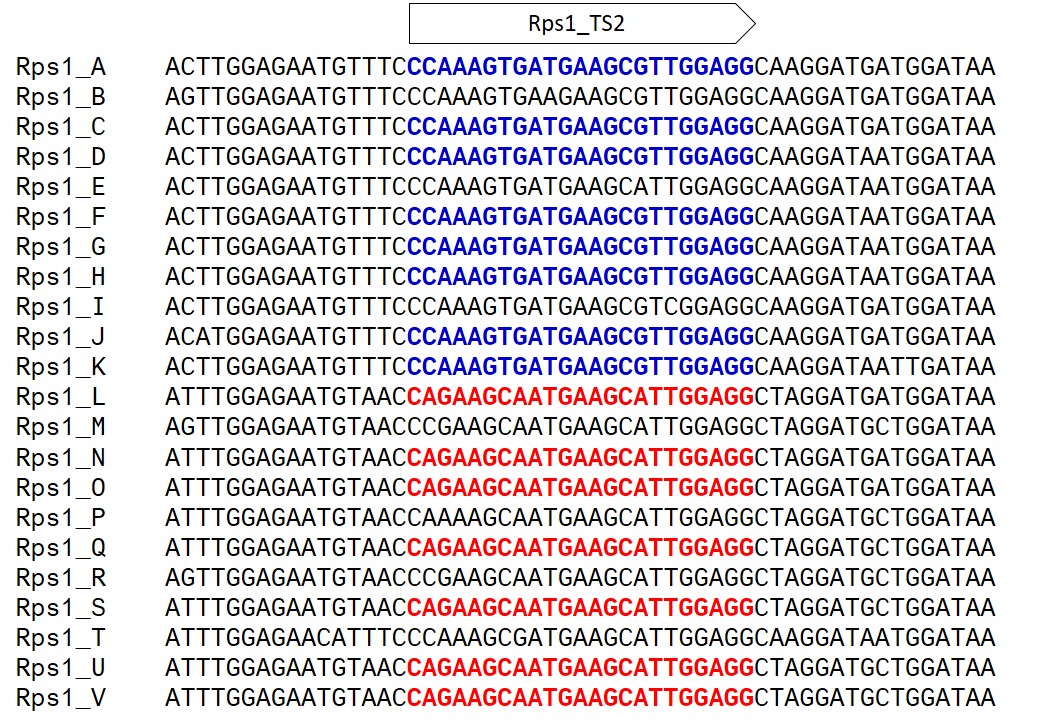


**Fig. S2** Quantification and validation of CRISPR/Cas9 activities in the R0 generation. High-resolution capillary electrophoresis was used to separate the amplicons spanning the target sites. All transformants, exemplified by mutants at the Rps1 target site TS4 (left panel) that included non-parental alleles were counted as mutants. Their total counts normalized by the population sizes gave rise to the mutation rates shown in the right panel. Three target regions failed to produce scorable assays in either of the two genotypes (no data, ND). Sequencing confirmed that targeted indels occurred at around the CRISPR/Cas9 target sites. Bottom panel shows examples of targeted indels at Rps1 TS4. Wild type target sites are underlined, mutant target sites are written in blue.


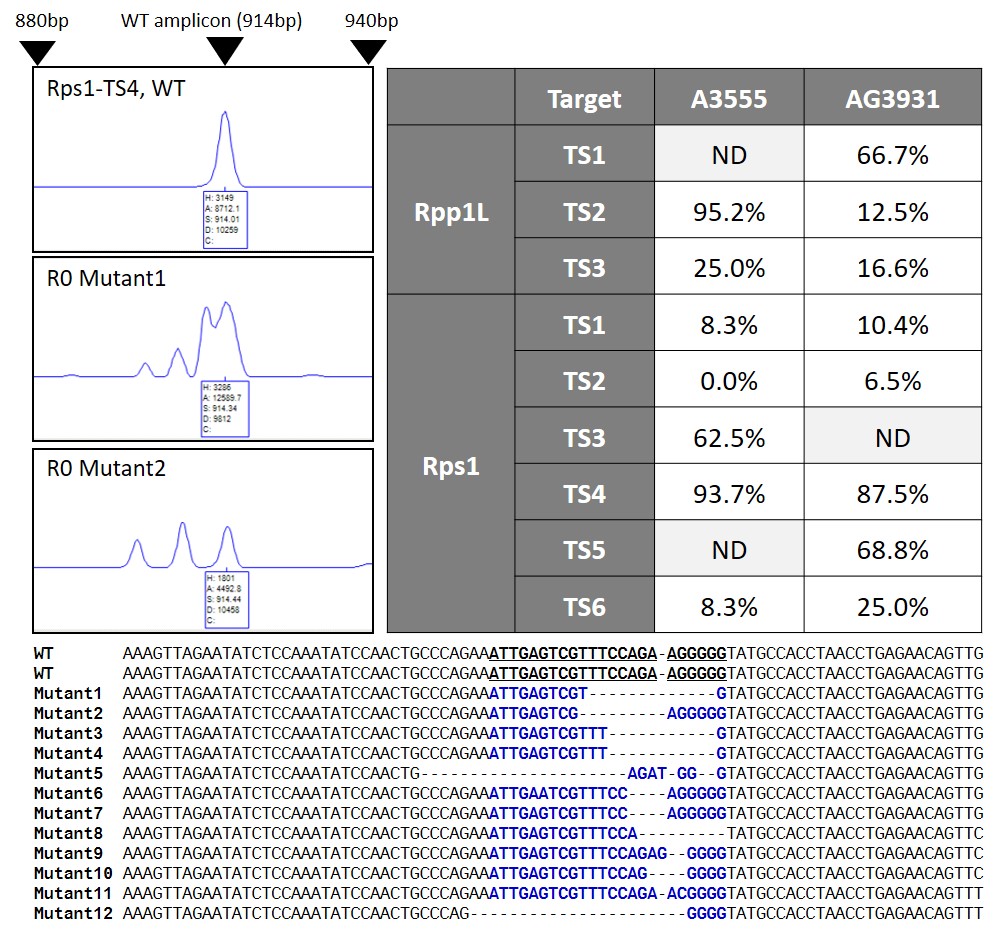


**Fig. S3** Validation of the Rpp1L- and Rps1-specific ddPCR assays. Three and four TaqMan assays were designed to detect copy number variations in the Rpp1L and Rps1 gene families, respectively. First, they were tested across a gDNA concentration gradient (0.04 to 5 ng/µl) in the A3555 (**a**) and AG3931 (**b**) transformation germplasms. Six of the seven assays showing consistency across various concentrations were further tested at optimal, 0.2 ng/µl gDNA concentration using four technical replicates (**c**). Based on their repeatability and on visual judgment of droplet clustering in their ddPCR profiles, two TaqMan assays, TM179/TM180/TM34P for Rpp1L and TM181/TM182/TM35P for Rps1 (highlighted in red) were selected for large-scale screening. Bars represent averages, error bars are standard deviations.

**a**


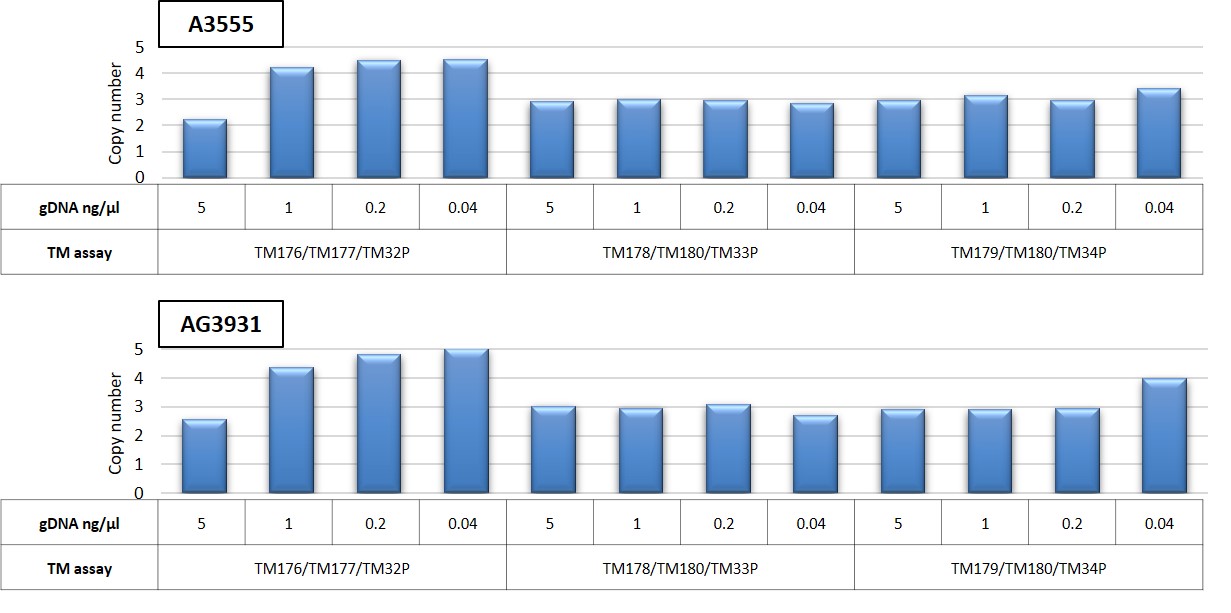


**b**


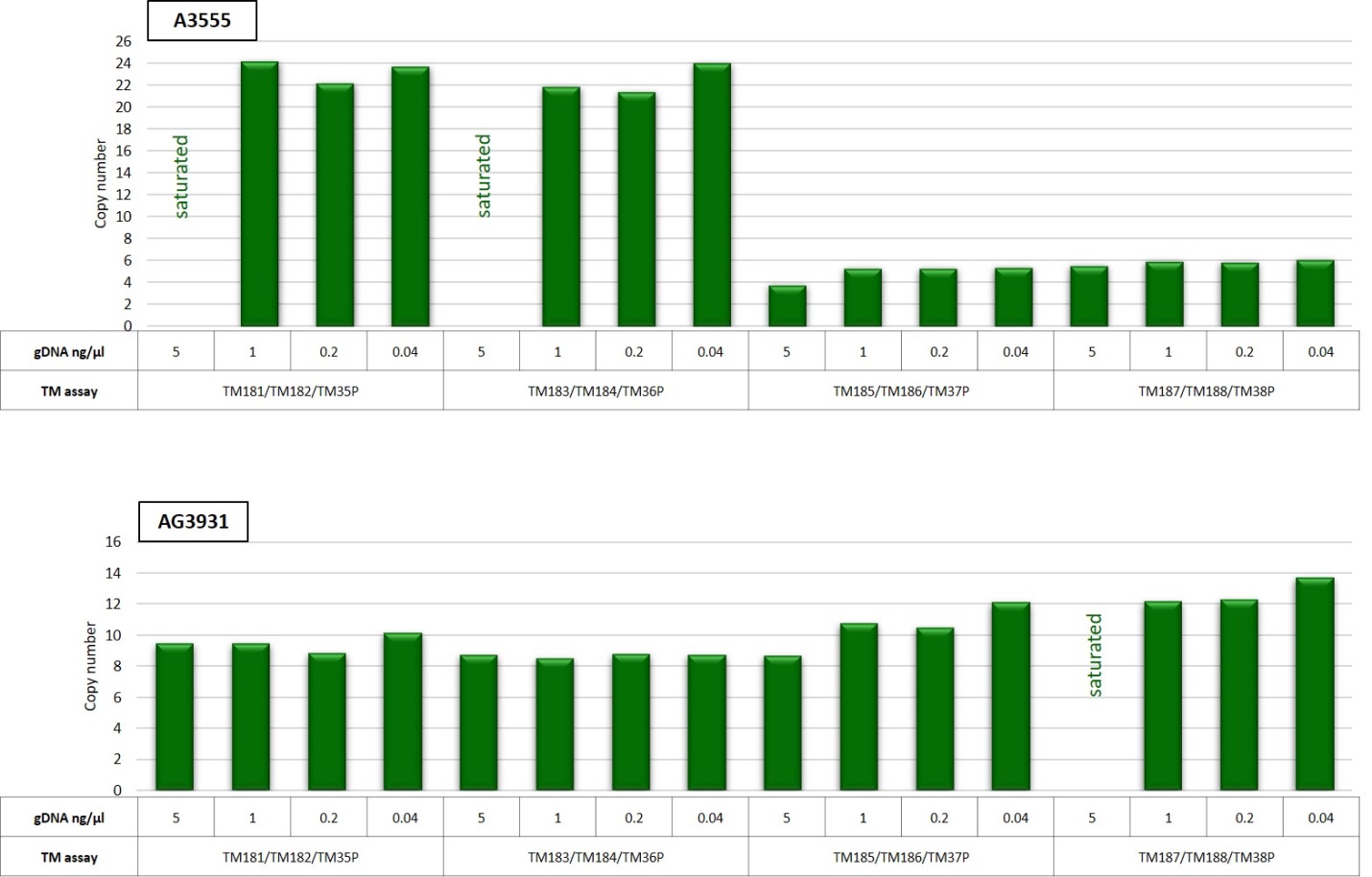


**c**

**
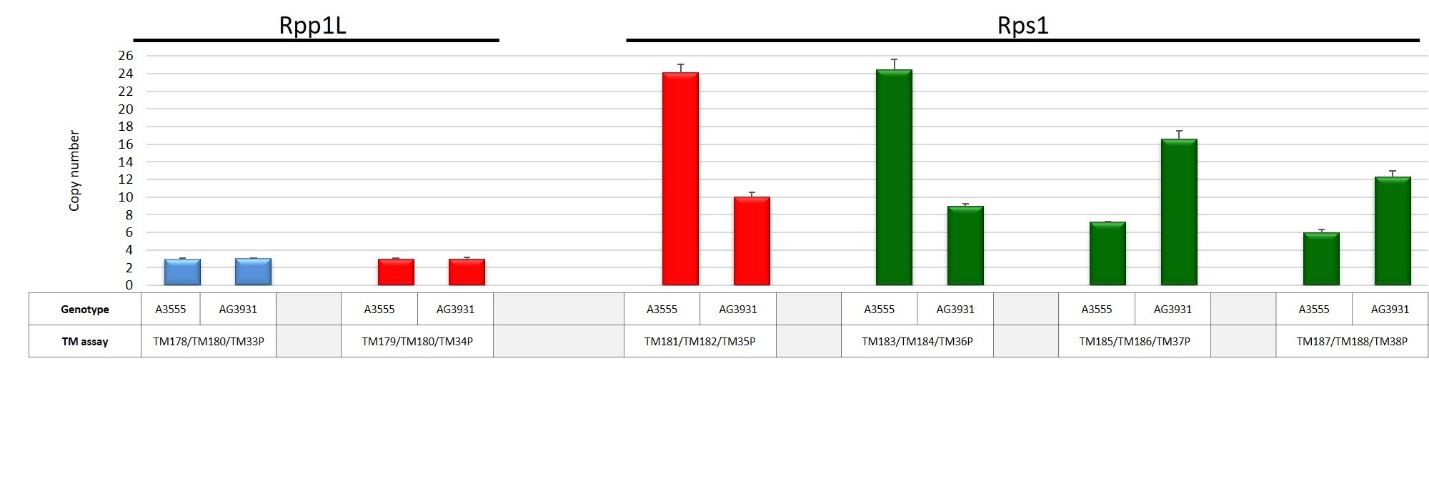
**

**Fig. S4** Inverse PCR to validate three novel chimeric Rpp1L paralogs carrying scarless A/C junctions at two target sites (TS1 and TS2). Genomic DNA was digested by AseI and NdeI restriction endonucleases, which produced a ~3.1kb fragment carrying all three target sites tested in this study. After self-circularization, amplicons spanning the AseI/NdeI junctions were generated by primers conserved between the A and C paralogs (solid black triangles). The A/C chromosomal junctions (empty triangles), confirming CRISPR/Cas9-mediated chimerizations were recovered for all three mutants.


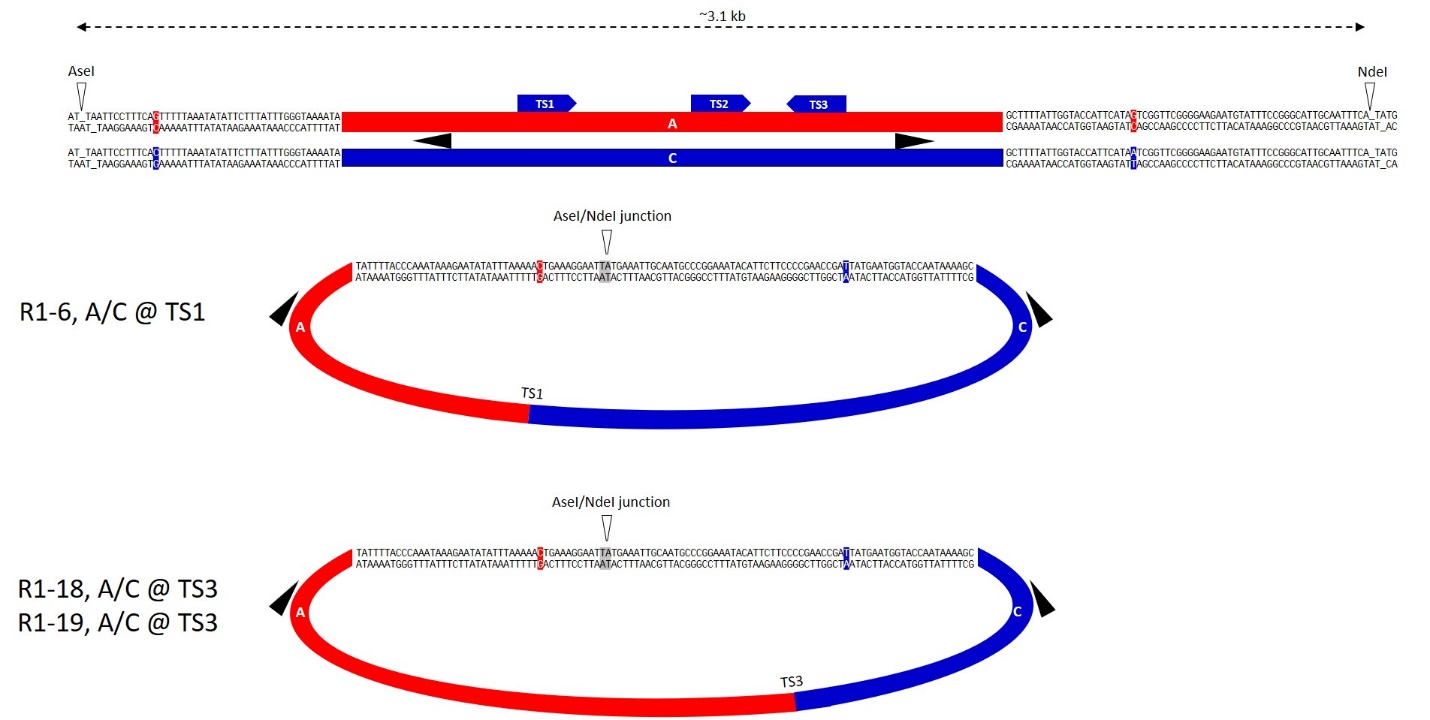


**Supplementary Data S1**

**Elements of CRISPR/Cas9 targeting constructs**

>Promoter, DaMV 35S

ATCAACGGAGAAACAAAGATAAAAATCAATTACTCACATGAAAGAGTATTGATCACGAGTCACTATGGAGCGACAATCTCCAGACAGGATGTCAGCATCTTATCTTCCTTTGAAGAAAGCATCATCAATAACGATGTAATGGTGGGGACATCCACTAAGTTATTGCTCTGCAAACAGCTCAAAAAGCTACTGGCCGACAATCATAATTGCTCGGCATGTGCAGGTGGGGCCTCCACTAGCAATAATACAAGCTTTACAGCTTGCAGTGACTCATCCTCCAATAATGGAGAAAAAGACGTCAGCAGTGACGAACAAGGGTCGAAAGACTTGCCTATATAAGGGCATTCTCCCCTCAGTTGAAGATC

>Leader, DaMV 35S

ATCGAAAGTTGGAGCAATAAACTCTCTCTTCAACAAATCTATCTTTTATCTTTTATC

>Nuclear localization Signal, HSFA1

GGATCTAAGAAGAGAAGAATTAAACAAGAT

>Open reading frame, Sp.Cas9 , the shaded sequence is the potato LS1 intron IV2

ATGGACAAGAAGTACAGCATTGGTCTCGATATCGGGACCAACTCCGTAGGCTGGGCCGTCATCACTGACGACTATAAGGTTCCCTCCAAGAAGTTCAAAGTGCTGGGCAACACGGATAGACACTCTATCAAGAAAAACCTGATTGGAGCCCTGTTGTTCGGTTCCGGCGAGACTGCGGAAGCGACTCGCTTGAAGCGCACTGCCCGCAGGAGGTATACGAGAAGGAAGAACAGGATTTGCTACCTGCAGGTAAGTTTCTGCTTCTACCTTTGATATATATATAATAATTATCATTAATTAGTAGTAATATAATATTTCAAATATTTTTTTCAAAATAAAAGAATGTAGTATATAGCAATTGCTTTTCTGTAGTTTATAAGTGTGTATATTTTAATTTATAACTTTTCTAATATATGACCAAAATTTGTTGATGTGCAGGAGATCTTCAGCAACGAGATGGCCAAGGTGGATGACAGCTTCTTCCACCGTTTGGAGGAGAGTTTCCTGGTCGAAGAAGATAAGAAGCACGAACGCCACCCGATTTTCGGCAATATAGTCGACGAGGTAGCCTATCACGAAAAGTATCCCACCATCTACCATCTCAGGAAAAAACTGGCGGACAGCACTGACAAGGCTGATCTAAGACTGATTTACCTCGCTCTTGCTCACATGATCAAGTTTCGCGGTCATTTCCTAATAGAAGGCGATCTGAATCCGGACAATAGCGACGTAGATAAACTCTTCATCCAGTTGGTCCAGATTTACAACCAGCTTTTTGAGGAGAACCCGATAAATGCCAGTCGCGTTGATGCAAAGGCCATACTCAGTGCACGACTTTCTAAGTCCAGAAGACTGGAGAATCTGATCGCACAGCTTCCCGGAGAAAAGCGAAATGGTCTGTTCGGCAACCTCATTGCGCTGTCTCTAGGCCTGACGCCTAACTTCAAGAGTAACTTCGATTTGGCAGAAGATGCTAAACTACAGCTTTCAAAGGACACCTACGATGACGACCTCGACAACTTGCTGGCTCAAATAGGGGACCAATACGCTGATCTGTTCTTAGCTGCGAAGAATCTGAGTGATGCAATTCTACTATCCGATATTCTTCGTGTTAACAGCGAAATTACAAAAGCGCCATTGTCTGCGAGTATGATCAAAAGATACGACGAACATCACCAGGATCTCACACTCCTCAAGGCCCTTGTTAGGCAGCAACTGCCGGAGAAGTACAAGGAAATCTTTTTCGACCAATCTAAAAATGGTTATGCCGGCTACATAGACGGCGGAGCATCACAGGAGGAGTTCTATAAATTCATCAAACCTATTTTGGAAAAGATGGATGGGACCGAGGAGCTTCTCGTGAAGCTAAACCGGGAGGATCTCCTGAGGAAACAAAGGACCTTTGACAACGGGTCTATACCCCACCAAATCCACTTGGGGGAACTTCACGCCATCCTTCGTCGGCAGGAAGACTTTTATCCCTTCTTGAAGGATAACAGGGAGAAGATCGAGAAGATCCTTACGTTCAGAATACCCTACTATGTGGGACCCTTAGCACGAGGAAACTCCCGTTTTGCATGGATGACCCGCAAATCAGAAGAGACAATCACGCCTTGGAACTTCGAGGAGGTGGTCGACAAGGGTGCCTCTGCTCAGAGTTTCATCGAGCGGATGACTAATTTTGACAAGAATCTCCCGAATGAGAAAGTACTTCCGAAGCACAGTCTGCTGTACGAATATTTTACTGTTTATAACGAATTGACTAAGGTCAAATACGTCACAGAAGGCATGAGAAAGCCTGCATTCCTGTCAGGGGAGCAAAAAAAGGCTATCGTTGATCTCCTTTTCAAAACGAACCGAAAGGTTACCGTTAAGCAGTTGAAAGAAGACTATTTCAAGAAAATCGAGTGCTTCGACTCTGTGGAGATTTCTGGTGTCGAGGATCGATTTAATGCCTCATTGGGTGCATACCATGATCTGCTTAAGATTATAAAAGATAAAGACTTCTTGGACAACGAAGAAAATGAGGATATTCTCGAAGATATTGTGCTGACCCTAACTTTGTTTGAAGATCGCGGAATGATAGAAGAGCGTCTAAAAACATACGCTCACTTATTCGATGACAAGGTCATGAAACAGCTTAAAAGAAGGAGATATACCGGCTGGGGACGCCTTTCTCGGAAACTCATCAATGGCATACGGGACAAGCAGTCTGGTAAAACTATATTGGATTTTTTGAAATCAGACGGCTTTGCAAATCGGAACTTTATGCAATTAATCCATGATGACTCTCTGACATTCAAGGAAGATATCCAGAAGGCCCAGGTTTCCGGACAAGGTCACTCTTTACACGAACAAATTGCAAATCTGGCCGGCTCCCCAGCTATTAAAAAAGGAATATTGCAAACCGTGAAGATCGTTGATGAGCTTGTCAAAGTTATGGGGCACAAACCGGAAAATATCGTCATAGAGATGGCTAGAGAGAATCAAACCACACAGAAGGGACAGAAAAACTCACGCGAGAGAATGAAGAGAATCGAAGAGGGTATTAAGGAGCTCGGTTCTCAAATCCTTAAAGAACACCCAGTAGAGAATACACAACTCCAGAATGAGAAGTTGTACCTATACTACCTGCAGAACGGAAGAGATATGTACGTAGACCAAGAACTTGATATTAACCGATTAAGTGATTATGACGTGGACCATATAGTTCCTCAATCCTTCATTAAGGATGACAGCATTGACAACAAGGTGCTTACCCGATCTGACAAGAACAGGGGGAAGAGTGATAATGTACCCTCCGAAGAGGTCGTCAAAAAAATGAAGAATTATTGGCGTCAACTGTTGAACGCTAAACTGATTACGCAGAGAAAGTTCGATAATCTTACAAAGGCAGAAAGAGGGGGTTTGTCTGAATTGGACAAAGCTGGGTTCATCAAACGACAGTTAGTTGAAACCCGACAGATCACAAAGCATGTGGCCCAAATTCTTGACTCTCGAATGAATACAAAGTATGACGAGAACGACAAACTAATCAGAGAAGTTAAGGTTATTACCCTCAAGTCTAAGCTCGTATCTGATTTTCGCAAAGATTTCCAGTTCTACAAGGTTCGCGAAATTAATAATTATCATCACGCTCACGATGCCTATCTTAACGCAGTCGTCGGAACGGCTCTCATCAAGAAATATCCGAAGCTCGAATCGGAGTTCGTGTATGGGGATTATAAAGTTTATGATGTGAGGAAAATGATTGCTAAGAGCGAACAGGAAATCGGCAAGGCAACAGCTAAATACTTTTTTTATTCAAACATCATGAACTTTTTTAAGACAGAGATTACACTGGCTAATGGAGAGATCCGCAAACGCCCGTTGATTGAGACCAATGGAGAAACCGGGGAGATCGTTTGGGACAAAGGAAGGGATTTTGCCACTGTTAGAAAAGTGCTGTCTATGCCTCAGGTTAATATCGTTAAGAAGACGGAGGTTCAGACAGGCGGCTTCTCTAAAGAGTCAATTCTCCCTAAAAGGAACAGTGACAAACTGATCGCGCGGAAAAAGGACTGGGATCCAAAGAAGTATGGGGGCTTTGACAGTCCGACCGTTGCTTACAGTGTTCTTGTGGTCGCAAAGGTCGAGAAGGGTAAGTCTAAAAAACTTAAAAGTGTTAAGGAGCTGCTCGGTATAACTATCATGGAGCGAAGCTCCTTTGAAAAGAACCCTATTGATTTCCTGGAGGCGAAGGGTTACAAAGAAGTGAAGAAGGATCTCATTATTAAGCTCCCAAAATACAGCCTTTTCGAGCTTGAAAACGGCAGAAAGCGGATGCTCGCCTCCGCTGGAGAGCTCCAGAAGGGAAACGAGCTTGCCCTCCCCAGTAAATATGTCAACTTCCTGTATCTGGCCAGCCACTACGAGAAGCTTAAAGGCTCTCCCGAGGACAACGAACAGAAACAACTGTTCGTGGAACAGCATAAGCATTACCTGGACGAGATCATCGAGCAAATATCAGAATTTTCTAAGCGGGTAATCCTGGCCGACGCAAACTTAGACAAAGTGTTAAGCGCATACAACAAGCATCGAGATAAGCCAATCAGAGAACAGGCGGAGAATATAATCCACTTGTTCACGCTCACCAATCTGGGCGCCCCTGCGGCGTTTAAATACTTCGACACTACAATTGACAGGAAGCGTTACACTTCTACTAAGGAGGTCTTGGACGCCACCCTTATTCATCAATCAATTACCGGCCTCTACGAAACCCGGATAGACCTGAGCCAGCTAGGTGGCGACTAA

>Terminator, Medicago truncatula AC140914v20

AAGGGCTCTCTGTCATGATTTCATACTTTCATTATTGAGCTCTGTAATTACAATTATGACCATGAGAACATCTCTTATTGTGTGGCCTTTTAATTGCTGATGTTAGTACTGAACCAAAGCTTATCGTGATGATGTAAAAGCAATAAGTACTTGTTTGTAGCTTCTTTGTGTCTCCCTTTGGGCTTAATACATCTGTTTAGTGTTGTGGCTTTGGCATAGACTTCTCTTGGTAATAATGCCTTGCAATGCAAAATTTCAATTATCAAATTCTATTATGTTCTCACCTTATGGTAACAGCTTACCCTGTGGAAGATGAGATTCTTGAGTTGAGTCATTGCCAATTTTTGGCATTAGCTTTTGAATTAGTGAATTTTGACAAAAATTACCGTGACACTGATTTTGTTGAAGCTCTTAAGTGTAGTTTTTACAAAATTTCAGTGGCTCGTTGTGATTATGTCAAACTCACGGCGAATGTAGTTCTTACAGAATTTCAGTGGCTC

>Promoter, U6i

AAACAAGCTAAAAGTTGCAAAAGAAATGGCAGGGCTATAAGGCTCACCTACTCCTGGATTTACCAAATTTTGGTTCGTCCCTATACTCGAAAAATAAAACAAAATAAATTTCAGTATCTTCGTTTTTGTATGCTTTGACTGTGAGGCGAGGCCAACTTTCTTCTTCTGTCTGAGATGAATTTTGTTTGCCTCCTGTGAAGGATGTATCATTCAAAGTGAATGTTTTGCAACTGCCAGTAGTCCCACATCGACCAAATATTCTTATTACAGTGTGTTTATATAGCACCTGGAGAAGGAATGGGTT

>Promoter, U6c

AAATGGTAAAATGTCAAATCAAAACTAGGCTGCAGTATGCAGAGCAGAGTCATGATGATACTACTTACTACACCGATTCTTGTGTGCAGAAAAATATGTTAAAATAATTGAATCTTTCTCTAGCCAAATTTGACAACAATGTACACCGTTCATATTGAGAGACGATGCTTCTTGTTTGCTTTCGGTGGAAGCTGCATATACTCAACATTACTCCTTCAGCGAGTTTTCCAACTGAGTCCCACATTGCCCAGACCTAACACGGTATTCTTGTTTATAATGAAATGTGCCACCACATGGATT

>sgRNA, conserved region including T(8) terminator (shaded)

GTTTTAGAGCTAGAAATAGCAAGTTAAAATAAGGCTAGTCCGTTATCAACTTGAAAAAGTGGCACCGAGTCGGTGCTTTTTTTT

**Supplementary Data S2**

**A3555 R1 mutants at TS1**

R1-1

>A3555_Paralog_A

TAGAGAAGCTTTGGGAGTTGCGAGATGAAAATGTGAAGAAGATGTGGGATCTTCTGGAGGATGAGGAAGTCTTCATTATTGGCATAGATGGAATGGGGGGAGTTGGAAAAACATTCATGGCAACTCATTTCAAGAATGAGATTAAAAGAAAGGGGACTTTC

>A_Ins_1bp_B

TAGAGAAGCTTTGGGAGTTGCGAGATGAAAATGTGAAGAAGATGTGGGATCTTCTGGAGGTATGAGGAAGTCTTCATTATTGGCATAGATGGAATGGGGGGAGTTGGAAAAACATTCATGGCAACTCATATCAAGAATGAGATTAAAAGAAAGGGGACTTTC

>A_Del_2bp_B

TAGAGAAGCTTTGGGAGTTGCGAGATGAAAATGTGAAGAAGATGTGGGATCTTCTGGAATGAGGAAGTCTTCATTATTGGCATAGATGGAATGGGGGGAGTTGGAAAAACATTCATGGCAACTCATATCAAGAATGAGATTAAAAGAAAGGGGACTTTC

>A_Del_3bp_B

TAGAGAAGCTTTGGGAGTTGCGAGATGAAAATGTGAAGAAGATGTGGGATCTTCTGGATGAGGAAGTCTTCATTATTGGCATAGATGGAATGGGGGGAGTTGGAAAAACATTCATGGCAACTCATATCAAGAATGAGATTAAAAGAAAGGGGACTTTC

>B_Del_28bp_B

TACAGAAGCCTTGGGGGTTGCGAGATAAAAAAAGAAGAAAGTCTTCATTATTGGCATAGATGGAATGGGGGGAGTTGGAAAAACATTCATGGCAACTCATATCAAGAATGAGATTAAAAGAAAGGGGACTTTC

>B_Del_6bp_B

TACAGAAGCCTTGGGGGTTGCGAGATAAAAATGTGAAGAAGATGTGGGATCTTCTGGAGGAAGTCTTCATTATTGGCATAGATGGAATGGGGGGAGTTGGAAAAACATTCATGGCAACTCATATCAAGAATGAGATTAAAAGAAAGGGGACTTTC

>B_Del3bp_B

TACAGAAGCCTTGGGGGTTGCGAGATAAAAATGTGAAGAAGATGTGGGATCTTCTGGATGAGGAAGTCTTCATTATTGGCATAGATGGAATGGGGGGAGTTGGAAAAACATTCATGGCAACTCATATCAAGAATGAGATTAAAAGAAAGGGGACTTTC

>A3555_Paralog_B

TACAGAAGCCTTGGGGGTTGCGAGATAAAAATGTGAAGAAGATGTGGGATCTTCTGGAGGATGAGGAAGTCTTCATTATTGGCATAGATGGAATGGGGGGAGTTGGAAAAACATTCATGGCAACTCATATCAAGAATGAGATTAAAAGAAAGGGGACTTTC

>A3555_Paralog_D

AAGAGAATCCTTGGGTGTCGCGAGATGAAAATGTGAAGGAGATGTGGGATCTTCTGGAGGATGAGGAAGTCTTCATTATTGGCATAGATGGAATGGGGGGAGTTGGAAAAACATTCATGGCAACTCATATCAAGAATGAGATTAAAAGAAAGGGGACTTTC

>D_Del4bp_D

AAGAGAATCCTTGGGTGTCGCGAGATGAAAATGTGAAGGAGATGTGGGATCTTCTGATGAGGAAGTCTTCATTATTGGCATAGATGGAATGGGGGGGAGTTGGAAAAACATTCATGGCAACTCATATCAAGAATGAGATTAAAAGAAAGGGGACTTTC

R1-2

>A3555_Paralog_B

TACAGAAGCCTTGGGGGTTGCGAGATAAAAATGTGAAGAAGATGTGGGATCTTCTGGAGGATGAGGAAGTCTTCATTATTGGCATAGATGGAATGGGGGGAGTTGGAAAAACATTCATGGCAACTCATATCAAGAATGAGATTAAAAGAAAGGGGACTTTC

>B_Sub3bp_B

TACAGAAGCCTTGGGGGGTTGCGAGATAAAAATGTGAAGAAGATGTGGAATCCTCTGGAGGAGGAGGAAGTCTTCATTATTGGCATAGATGGAATGGGGGGAGTTGGAAAAACATTCATGGCAACTCATATCAAGAATGAGATTAAAAGAAAGGGGACTTTC

>B_Del1bp_B

TACAGAAGCCTTGGGGGTTGCGAGATAAAAATGTGAAGAAGATGTGGGATCTTCTGGAGATGAGGAAGTCTTCATTATTGGCATAGATGGAATGGGGGGAGTTGGAAAAACATTCATGGCAACTCATATCAAGAATGAGATTAAAAGAAAGGGGACTTTC

>B_Del2bp_B

TACAGAAGCCTTGGGGGTTGCGAGATAAAAATGTGAAGAAGATGTGGGATCTTCTGGAATGAGGAAGTCTTCATTATTGGCATAGATGGAATGGGGGGAGTTGGAAAAACATTCATGGCAACTCATATCAAGAATGAGATTAAAAGAAAGGGGACTTTC

>B_Del22bp_B

TACAGAAGCCTTGGGGGTTGCGAGATAAAAATGTGAAGAAGATGTGGGATCTTCTGGAGGTATAGATGGAATGGGGGGAGTTGGAAAAACATTCATGGCAACTCATATCAAGAATGAGATTAAAAGAAAGGGGACTTTC

>B_Del89bp_B

TACAGAAGCCTTGGGGGTTGCGAGATAAAAATGTGAAGAAGATGTGGGATCTTCTGGAGAAAGGGGACTTTC

>A3555_Paralog_D

AAGAGAATCCTTGGGTGTCGCGAGATGAAAATGTGAAGGAGATGTGGGATCTTCTGGAGGATGAGGAAGTCTTCATTATTGGCATAGATGGAATGGGGGGAGTTGGAAAAACATTCATGGCAACTCATATCAAGAATGAGATTAAAAGAAAGGGGACTTTC

>D_Del21bp_D

AAGAGAATCCTTGGGTGTCGCGAGATGAAAATGTGAAGGAGATGTGGGATCTTCTGGAGGAATGGGGGGAGTTGGAAAAACATTCATGGCAACTCATATCAAGAATGAGATTAAAAGAAAGGGGACTTTC

R1-3

>A3555_Paralog_C

TAGAGAAGCGTTGGGCGTCGCGAGATAAAAAAGTGGAGAACATGTGGGATCTTCTGGAGGATGAGGAAGTCTTCATTATTGGCATAGATGGAATGGGGGGAGTTGGAAAAACATTCATGGCAACTCATATCAAGAATGAGATTAAAAGAAAGGGGACTTTC

>C_Del2bp_C

TAGAGAAGCGTTGGGCGTCGCGAGATAAAAAAGTGGAGAACATGTGGGATCTTCTGGAATGAGGAAGTCTTCATTATTGGCATAGATGGAATGGGGGGAGTTGGAAAAACATTCATGGCAACTCATATCAAGAATGAGATTAAAAGAAAGGGGACTTTC

R1-4

>A3555_Paralog_A

GCCCAAGCCTTCTGAATTGCATGCTAAGTTTATTTATTTATTGATAGAGAAGCTTTGGGAGTTGCGAGATGAAAATGTGAAGAAGATGTGGGATCTTCTGGAGGATGAGGAAGTCTTCATTATTGGCATAGATGGAATGGGGGGAGTTGGAAAAACATTCATGGCAACTCATTTCAAGAATGAGATTAAAAGAAAGGGGACTTTC

>A_Del71bp_B

GCCCAAGCCTTCTGAATTGCATGCTAAGTCTTCATTATTGGCATAGATGGAATGGGGGGAGTTGGAAAAACATTCATGGCAACTCATATCAAGAATGAGATTAAAAGAAAGGGGACTTTC

>A3555_Paralog_B

GGATGAGCCTTCTGAATTGGATATTGAGTTTGATGTATTATTGATACAGAAGCCTTGGGGGTTGCGAGATAAAAATGTGAAGAAGATGTGGGATCTTCTGGAGGATGAGGAAGTCTTCATTATTGGCATAGATGGAATGGGGGGAGTTGGAAAAACATTCATGGCAACTCATATCAAGAATGAGATTAAAAGAAAGGGGACTTTC

>A3555_Paralog_C

TAGAGAAGCGTTGGGCGTCGCGAGATAAAAAAGTGGAGAACATGTGGGATCTTCTGGAGGATGAGGAAGTCTTCATTATTGGCATAGATGGAATGGGGGGAGTTGGAAAAACATTCATGGCAACTCATATCAAGAATGAGATTAAAAGAAAGGGGACTTTC

>Del2bp_C

TAGAGAAGCGTTGGGCGTCGCGAGATAAAAAAGTGGAGAACATGTGGGATCTTCTGGAATGAGGAAGTCTTCATTATTGGCATAGATGGAATGGGGGGAGTTGGAAAAACATTCATGGCAACTCATATCAAGAATGAGATTAAAAGAAAGGGGACTTTC

R1-5

>A3555_Paralog_A

TAGAGAAGCTTTGGGAGTTGCGAGATGAAAATGTGAAGAAGATGTGGGATCTTCTGGAGGATGAGGAAGTCTTCATTATTGGCATAGATGGAATGGGGGGAGTTGGAAAAACATTCATGGCAACTCATTTCAAGAATGAGATTAAAAGAAAGGGGACTTTC

>A_Del3bp_A

TAGAGAAGCTTTGGGAGTTGCGAGATGAAAATGTGAAGAAGATGTGGGATCTTCTGGAGGAGGAAGTCTTCATTATTGGCATAGATGGAATGGGGGGAGTTGGAAAAACATTCATGGCAACTCATTTCAAGAATGAGATTAAAAGAAAGGGGACTTTC

>A3555_Paralog_C

TAGAGAAGCGTTGGGCGTCGCGAGATAAAAAAGTGGAGAACATGTGGGATCTTCTGGAGGATGAGGAAGTCTTCATTATTGGCATAGATGGAATGGGGGGAGTTGGAAAAACATTCATGGCAACTCATATCAAGAATGAGATTAAAAGAAAGGGGACTTTC

>C_Del19bp_C

TAGAGAAGCGTTGGGCGTCGCGAGATAAAAAAGTGGAGAACATGAGGAAGTCTTCATTATTGGCATAGATGGAATGGGGGGAGTTGGAAAAACATTCATGGCAACTCATATCAAGAATGAGATTAAAAGAAAGGGGACTTTC

R1-6

>A3555_Paralog_A

TAGAGAAGCTTTGGGAGTTGCGAGATGAAAATGTGAAGAAGATGTGGGATCTTCTGGAGGATGAGGAAGTCTTCATTATTGGCATAGATGGAATGGGGGGAGTTGGAAAAACATTCATGGCAACTCATTTCAAGAATGAGATTAAAAGAAAGGGGACTTTC

>A_Intact_A

TAGAGAAGCTTTGGGAGTTGCGAGATGAAAATGTGAAGAAGATGTGGGATCTTCTGGAGGATGAGGAAGTCTTCATTATTGGCATAGATGGAATGGGGGGAGTTGGAAAAACATTCATGGCAACTCATTTCAAGAATGAGATTAAAAGAAAGGGGACTTTC

>A_Intact_C

TAGAGAAGCTTTGGGAGTTGCGAGATGAAAATGTGAAGAAGATGTGGGATCTTCTGGAGGATGAGGAAGTCTTCATTATTGGCATAGATGGAATGGGGGGAGTTGGAAAAACATTCATGGCAACTCATATCAAGAATGAGATTAAAAGAAAGGGGACTTTC

>C_Del6bp_C

TAGAGAAGCGTTGGGCGTCGCGAGATAAAAAAGTGGAGAACATGTGGGATCTTCATGAGGAAGTCTTCATTATTGGCATAGATGGAATGGGGGGAGTTGGAAAAACATTCATGGCAACTCATATCAAGAATGAGATTAAAAGAAAGGGGACTTTC

>A3555_Paralog_C

TAGAGAAGCGTTGGGCGTCGCGAGATAAAAAAGTGGAGAACATGTGGGATCTTCTGGAGGATGAGGAAGTCTTCATTATTGGCATAGATGGAATGGGGGGAGTTGGAAAAACATTCATGGCAACTCATATCAAGAATGAGATTAAAAGAAAGGGGACTTTC

R1-7

>A3555_Paralog_A

TAGAGAAGCTTTGGGAGTTGCGAGATGAAAATGTGAAGAAGATGTGGGATCTTCTGGAGGATGAGGAAGTCTTCATTATTGGCATAGATGGAATGGGGGGAGTTGGAAAAACATTCATGGCAACTCATTTCAAGAATGAGATTAAAAGAAAGGGGACTTTC

>A_Intact_A

TAGAGAAGCTTTGGGAGTTGCGAGATGAAAATGTGAAGAAGATGTGGGATCTTCTGGAGGATGAGGAAGTCTTCATTATTGGCATAGATGGAATGGGGGGAGTTGGAAAAACATTCATGGCAACTCATTTCAAGAATGAGATTAAAAGAAAGGGGACTTTC

>A_Del1bp_A

TAGAGAAGCTTTGGGAGTTGCGAGATGAAAATGTGAAGAAGATGTGGGATCTTCTGGAGATGAGGAAGTCTTCATTATTGGCATAGATGGAATGGGGGGAGTTGGAAAAACATTCATGGCAACTCATTTCAAGAATGAGATTAAAAGAAAGGGGACTTTC

>A3555_Paralog_B

TACAGAAGCCTTGGGGGTTGCGAGATAAAAATGTGAAGAAGATGTGGGATCTTCTGGAGGATGAGGAAGTCTTCATTATTGGCATAGATGGAATGGGGGGAGTTGGAAAAACATTCATGGCAACTCATATCAAGAATGAGATTAAAAGAAAGGGGACTTTC

>B_Intact_B

TACAGAAGCCTTGGGGGTTGCGAGATAAAAATGTGAAGAAGATGTGGGATCTTCTGGAGGATGAGGAAGTCTTCATTATTGGCATAGATGGAATGGGGGGAGTTGGAAAAACATTCATGGCAACTCATATCAAGAATGAGATTAAAAGAAAGGGGACTTTC

>A3555_Paralog_C

TAGAGAAGCGTTGGGCGTCGCGAGATAAAAAAGTGGAGAACATGTGGGATCTTCTGGAGGATGAGGAAGTCTTCATTATTGGCATAGATGGAATGGGGGGAGTTGGAAAAACATTCATGGCAACTCATATCAAGAATGAGATTAAAAGAAAGGGGACTTTC

>C_Del6bp_C

TAGAGAAGCGTTGGGCGTCGCGAGATAAAAAAGTGGAGAACATGTGGGATCTTCTGGAGGAAGTCTTCATTATTGGCATAGATGGAATGGGGGGAGTTGGAAAAACATTCATGGCAACTCATATCAAGAATGAGATTAAAAGAAAGGGGACTTTC

R1-8

>A3555_Paralog_A

TAGAGAAGCTTTGGGAGTTGCGAGATGAAAATGTGAAGAAGATGTGGGATCTTCTGGAGGATGAGGAAGTCTTCATTATTGGCATAGATGGAATGGGGGGAGTTGGAAAAACATTCATGGCAACTCATTTCAAGAATGAGATTAAAAGAAAGGGGACTTTC

>A_Intact_A

TAGAGAAGCTTTGGGAGTTGCGAGATGAAAATGTGAAGAAGATGTGGGATCTTCTGGAGGATGAGGAAGTCTTCATTATTGGCATAGATGGAATGGGGGGAGTTGGAAAAACATTCATGGCAACTCATTTCAAGAATGAGATTAAAAGAAAGGGGACTTTC

>A_Del2bp_A

TAGAGAAGCTTTGGGAGTTGCGAGATGAAAATGTGAAGAAGATGTGGGATCTTCTGGAATGAGGAAGTCTTCATTATTGGCATAGATGGAATGGGGGGAGTTGGAAAAACATTCATGGCAACTCATTTCAAGAATGAGATTAAAAGAAAGGGGACTTTC

**A3555 R1 mutants at TS2**

R1-9

>A3555_Paralog_B

CCATATCTTGAACGAGAATCACACTTACATGATAAAATGTGATGAAAATTTGAGAAAGATACCTCAGATGCGGGAATGGACAGCTGATCTGGAGGCAGTTTCTTTGGCGGGTAATGAGATAGAAGAAATAGCAGAGGGCACATCACCTAATTGTCCAGGCTTGTCCACCTTGATTTTATCTCATAATTTGA

>B_Del1bp_B

CCATATCTTGAACGAGAATCACACTTACATGATAAAATGTGATGAAAATTTGAGAAAGATACCTCAGATGCGGGAATGGACAGCTGATTGGAGGCAGTTTCTTTGGCGGGTAATGAGATAGAAGAAATAGCAGAGGGCACATCACCTAATTGTCCAGGCTTGTCCACCTTGATTTTATCTCATAATTTGA

>B_Del3bp_B

CCATATCTTGAACGAGAATCACACTTACATGATAAAATGTGATGAAAATTTGAGAAAGATACCTCAGATGCGGGAATGGACAGCTGTGGAGGCAGTTTCTTTGGCGGGTAATGAGATAGAAGAAATAGCAGAGGGCACATCACCTAATTGTCCAGGCTTGTCCACCTTGATTTTATCTCATAATTTGA

>B_Del67bp_B

CCATATCTTGAACGAGAATCACACTTACATGATAAAATGTGATGAAAATTTGAGAAAGATACCTCAGATGCGGGAATGGACAGCTGTCCAGGCTTGTCCACCTTGATTTTATCTCATAATTTGA

>A3555_Paralog_C

CAATATCTTGAACGAGAATCACACTTACATGATAAAATGTCATGAAAATTTGAGAAAGATACCTCAGATGCGGGAATGGACAGCTGATCTGGAGGCAGTTTCTTTGGCGGGTAATGAGATAGAAGAAATAGCAGAGGGCACATCACCTAATTGTCCACGCTTGTCCACCTTCATCTTATCTCGTAATTCCA

>C_Del1bp_C

CAATATCTTGAACGAGAATCACACTTACATGATAAAATGTCATGAAAATTTGAGAAAGATACCTCAGATGCGGGAATGGACAGCTGATTGGAGGCAGTTTCTTTGGCGGGTAATGAGATAGAAGAAATAGCAGAGGGCACATCACCTAATTGTCCACGCTTGTCCACCTTCATCTTATCTCGTAATTCCA

R1-10

>A3555_Paralog_B

CCATATCTTGAACGAGAATCACACTTACATGATAAAATGTGATGAAAATTTGAGAAAGATACCTCAGATGCGGGAATGGACAGCTGATCTGGAGGCAGTTTCTTTGGCGGGTAATGAGATAGAAGAAATAGCAGAGGGCACATCACCTAATTGTCCAGGCTTGTCCACCTTGATTTTATCTCATAATTTGA

>B_Del1bp_B

CCATATCTTGAACGAGAATCACACTTACATGATAAAATGTGATGAAAATTTGAGAAAGATACCTCAGATGCGGGAATGGACAGCTGATTGGAGGCAGTTTCTTTGGCGGGTAATGAGATAGAAGAAATAGCAGAGGGCACATCACCTAATTGTCCAGGCTTGTCCACCTTGATTTTATCTCATAATTTGA

>B_Del2bp_B

CCATATCTTGAACGAGAATCACACTTACATGATAAAATGTGATGAAAATTTGAGAAAGATACCTCAGATGCGGGAATGGACAGCTGATGGAGGCAGTTTCTTTGGCGGGTAATGAGATAGAAGAAATAGCAGAGGGCACATCACCTAATTGTCCAGGCTTGTCCACCTTGATTTTATCTCATAATTTGA

>B_Del7bp_B

CCATATCTTGAACGAGAATCACACTTACATGATAAAATGTGATGAAAATTTGAGAAAGATACCTCAGATGCGGGAATGGACATGGAGGCAGTTTCTTTGGCGGGTAATGAGATAGAAGAAATAGCAGAGGGCACATCACCTAATTGTCCAGGCTTGTCCACCTTGATTTTATCTCATAATTTGA

>B_Del10bp_B

CCATATCTTGAACGAGAATCACACTTACATGATAAAATGTGATGAAAATTTGAGAAAGATACCTCAGATGCGGGAATGGTGGAGGCAGTTTCTTTGGCGGGTAATGAGATAGAAGAAATAGCAGAGGGCACATCACCTAATTGTCCAGGCTTGTCCACCTTGATTTTATCTCATAATTTGA

>B_Del34bp_B

CCATATCTTGAACGAGAATCACACTTACATGATAAAATGTGATGAAAATTTGAGAAAGATACCTCAGATGCGGGAATGAATGAGATAGAAGAAATAGCAGAGGGCACATCACCTAATTGTCCAGGCTTGTCCACCTTGATTTTATCTCATAATTTGA

R1-11

>A3555_Paralog_B

CCATATCTTGAACGAGAATCACACTTACATGATAAAATGTGATGAAAATTTGAGAAAGATACCTCAGATGCGGGAATGGACAGCTGATCTGGAGGCAGTTTCTTTGGCGGGTAATGAGATAGAAGAAATAGCAGAGGGCACATCACCTAATTGTCCAGGCTTGTCCACCTTGATTTTATCTCATAATTTGA

>B_Intact_B

CCATATCTTGAACGAGAATCACACTTACATGATAAAATGTGATGAAAATTTGAGAAAGATACCTCAGATGCGGGAATGGACAGCTGATCTGGAGGCAGTTTCTTTGGCGGGTAATGAGATAGAAGAAATAGCAGAGGGCACATCACCTAATTGTCCAGGCTTGTCCACCTTGATTTTATCTCATAATTTGA

>B_Del8bp_B

CCATATCTTGAACGAGAATCACACTTACATGATAAAATGTGATGAAAATTTGAGAAAGATACCTCAGATGCGGGAATGGACTGGAGGCAGTTTCTTTGGCGGGTAATGAGATAGAAGAAATAGCAGAGGGCACATCACCTAATTGTCCAGGCTTGTCCACCTTGATTTTATCTCATAATTTGA

>A3555_Paralog_C

CAATATCTTGAACGAGAATCACACTTACATGATAAAATGTCATGAAAATTTGAGAAAGATACCTCAGATGCGGGAATGGACAGCTGATCTGGAGGCAGTTTCTTTGGCGGGTAATGAGATAGAAGAAATAGCAGAGGGCACATCACCTAATTGTCCACGCTTGTCCACCTTCATCTTATCTCGTAATTCCA

>C_Intact_C

CAATATCTTGAACGAGAATCACACTTACATGATAAAATGTCATGAAAATTTGAGAAAGATACCTCAGATGCGGGAATGGACAGCTGATCTGGAGGCAGTTTCTTTGGCGGGTAATGAGATAGAAGAAATAGCAGAGGGCACATCACCTAATTGTCCACGCTTGTCCACCTTCATCTTATCTCGTAATTCCA

>C_Del1bp_C

CAATATCTTGAACGAGAATCACACTTACATGATAAAATGTCATGAAAATTTGAGAAAGATACCTCAGATGCGGGAATGGACAGCTGATTGGAGGCAGTTTCTTTGGCGGGTAATGAGATAGAAGAAATAGCAGAGGGCACATCACCTAATTGTCCACGCTTGTCCACCTTCATCTTATCTCGTAATTCCA

R1-12

>A3555_Paralog_A

CCATATCTTGAACGACAATCACACTTACTTGATAAAATGTAATGAAAAATTGAGAAAGATGCCTCAGATGCGGGAATGGACAGCTGATCTGGAGGCAGTTTCTTTGGCGGGTAATGAGATAGAAGAAATAGCAGAGGGCACATCACCTAATTGTCCACGCTTGTCCACCTTCATCTTATCTCGTAATTCCA

>A_Del8bp_A

CCATATCTTGAACGACAATCACACTTACTTGATAAAATGTAATGAAAAATTGAGAAAGATGCCTCAGATGCGGGAATGGACTGGAGGCAGTTTCTTTGGCGGGTAATGAGATAGAAGAAATAGCAGAGGGCACATCACCTAATTGTCCACGCTTGTCCACCTTCATCTTATCTCGTAATTCCA

>A3555_Paralog_B

CCATATCTTGAACGAGAATCACACTTACATGATAAAATGTGATGAAAATTTGAGAAAGATACCTCAGATGCGGGAATGGACAGCTGATCTGGAGGCAGTTTCTTTGGCGGGTAATGAGATAGAAGAAATAGCAGAGGGCACATCACCTAATTGTCCAGGCTTGTCCACCTTGATTTTATCTCATAATTTGA

>B_Del1bp_B

CCATATCTTGAACGAGAATCACACTTACATGATAAAATGTGATGAAAATTTGAGAAAGATACCTCAGATGCGGGAATGGACAGCTGATTGGAGGCAGTTTCTTTGGCGGGTAATGAGATAGAAGAAATAGCAGAGGGCACATCACCTAATTGTCCAGGCTTGTCCACCTTGATTTTATCTCATAATTTGA

>B_Del4bp_B

CCATATCTTGAACGAGAATCACACTTACATGATAAAATGTGATGAAAATTTGAGAAAGATACCTCAGATGCGGGAATGGACAGCTTGGAGGCAGTTTCTTTGGCGGGTAATGAGATAGAAGAAATAGCAGAGGGCACATCACCTAATTGTCCAGGCTTGTCCACCTTGATTTTATCTCATAATTTGA

>Del8bp_B

CCATATCTTGAACGAGAATCACACTTACATGATAAAATGTGATGAAAATTTGAGAAAGATACCTCAGATGCGGGAATGGACAGGAGGCAGTTTCTTTGGCGGGTAATGAGATAGAAGAAATAGCAGAGGGCACATCACCTAATTGTCCAGGCTTGTCCACCTTGATTTTATCTCATAATTTGA

>A3555_Paralog_C

CAATATCTTGAACGAGAATCACACTTACATGATAAAATGTCATGAAAATTTGAGAAAGATACCTCAGATGCGGGAATGGACAGCTGATCTGGAGGCAGTTTCTTTGGCGGGTAATGAGATAGAAGAAATAGCAGAGGGCACATCACCTAATTGTCCACGCTTGTCCACCTTCATCTTATCTCGTAATTCCA

>C_Del37bp_C

CAATATCTTGAACGAGAATCACACTTACATGATAAAATGTCATGAAAATTTGAGAAAGATACCTCAGATGCGGGTAATGAGATAGAAGAAATAGCAGAGGGCACATCACCTAATTGTCCACGCTTGTCCACCTTCATCTTATCTCGTAATTCCA

R1-13

>A3555_Paralog_A

CCATATCTTGAACGACAATCACACTTACTTGATAAAATGTAATGAAAAATTGAGAAAGATGCCTCAGATGCGGGAATGGACAGCTGATCTGGAGGCAGTTTCTTTGGCGGGTAATGAGATAGAAGAAATAGCAGAGGGCACATCACCTAATTGTCCACGCTTGTCCACCTTCATCTTATCTCGTAATTCCA

>A_Intact_A

CCATATCTTGAACGACAATCACACTTACTTGATAAAATGTAATGAAAAATTGAGAAAGATGCCTCAGATGCGGGAATGGACAGCTGATCTGGAGGCAGTTTCTTTGGCGGGTAATGAGATAGAAGAAATAGCAGAGGGCACATCACCTAATTGTCCACGCTTGTCCACCTTCATCTTATCTCGTAATTCCA

>A_Del2bp_A

CCATATCTTGAACGACAATCACACTTACTTGATAAAATGTAATGAAAAATTGAGAAAGATGCCTCAGATGCGGGAATGGACAGCTGATGGAGGCAGTTTCTTTGGCGGGTAATGAGATAGAAGAAATAGCAGAGGGCACATCACCTAATTGTCCACGCTTGTCCACCTTCATCTTATCTCGTAATTCCA

R1-14

>A3555_Paralog_A

CCATATCTTGAACGACAATCACACTTACTTGATAAAATGTAATGAAAAATTGAGAAAGATGCCTCAGATGCGGGAATGGACAGCTGATCTGGAGGCAGTTTCTTTGGCGGGTAATGAGATAGAAGAAATAGCAGAGGGCACATCACCTAATTGTCCACGCTTGTCCACCTTCATCTTATCTCGTAATTCCA

>A_Del6bp_A

CCATATCTTGAACGACAATCACACTTACTTGATAAAATGTAATGAAAAATTGAGAAAGATGCCTCAGATGCGGGAATGGACAGCTGAGGCAGTTTCTTTGGCGGGTAATGAGATAGAAGAAATAGCAGAGGGCACATCACCTAATTGTCCACGCTTGTCCACCTTCATCTTATCTCGTAATTCCA

R1-15

>A3555_Paralog_A

CATATCTTGAACGACAATCACACTTACTTGATAAAATGTAATGAAAAATTGAGAAAGATGCCTCAGATGCGGGAATGGACAGCTGATCTGGAGGCAGTTTCTTTGGCGGGTAATGAGATAGAAGAAATAGCAGAGGGCACATCACCTAATTGTCCACGCTTGTCCACCTTCATCTTATCTCGTAATTCCATCAGTCATATTCCCAAGTGTTTTTTCAGACGCATGAACGCTCTAACACAACTTGATTTATCATTTAATCTT

>A_Del12bp_C

CATATCTTGAACGACAATCACACTTACTTGATAAAATGTAATGAAAAATTGAGAAAGATGCCTCAGATGCGGGAATGGACAGCAGTTTCTTTGGCGGGTAATGAGATAGAAGAAATAGCAGAGGGCACATCACCTAATTGTCCACGCTTGTCCACCTTCATCTTATCTCGTAATTCCATCAGTCATATTCCCAAGTGTTTTTTCAGACACATGAACGCTCTAACACAACTTGATTTATCATATAATCGT

>A_Del12bpIns52bp_C

CATATCTTGAACGACAATCACACTTACTTGATAAAATGTAATGAAAAATTGAGAAAGATGCCTCAGATGCGGGAATGGACAGCATCTATCTCATTACCCGCCAGAGGACAGCATCTATCTCATCAGTGGATCTCAAGTTTCTTTGGCGGGTAATGAGATAGAAGAAATAGCAGAGGGCACATCACCTAATTGTCCACGCTTGTCCACCTTCATCTTATCTCGTAATTCCATCAGTCATATTCCCAAGTGTTTTTTCAGACACATGAACGCTCTAACACAACTTGATTTATCATATAATCGT

>A3555_Paralog_C

AATATCTTGAACGAGAATCACACTTACATGATAAAATGTCATGAAAATTTGAGAAAGATACCTCAGATGCGGGAATGGACAGCTGATCTGGAGGCAGTTTCTTTGGCGGGTAATGAGATAGAAGAAATAGCAGAGGGCACATCACCTAATTGTCCACGCTTGTCCACCTTCATCTTATCTCGTAATTCCATCAGTCATATTCCCAAGTGTTTTTTCAGACACATGAACGCTCTAACACAACTTGATTTATCATATAATCGT

>A3555_Paralog_D

CAATATCTTGAACGAGAATCACACTTACATGATAAAATGTCATGAAAATTTGACAAAGATACCTCAGATGCGGGAATGGACAGCTGATCTGGAGGCCGTTTCTTTGGCGGGTAATGAGATAGAAGAAATAGCAGAGGGCACATCACCTAATTGTCCTCGCTTGTCCACCTTCATCTTATCTCGTAATTCCA

>D_Intact_D

CAATATCTTGAACGAGAATCACACTTACATGATAAAATGTCATGAAAATTTGACAAAGATACCTCAGATGCGGGAATGGACAGCTGATCTGGAGGCCGTTTCTTTGGCGGGTAATGAGATAGAAGAAATAGCAGAGGGCACATCACCTAATTGTCCTCGCTTGTCCACCTTCATCTTATCTCGTAATTCCA

R1-16

>A3555_Paralog_A

CCATATCTTGAACGACAATCACACTTACTTGATAAAATGTAATGAAAAATTGAGAAAGATGCCTCAGATGCGGGAATGGACAGCTGATCTGGAGGCAGTTTCTTTGGCGGGTAATGAGATAGAAGAAATAGCAGAGGGCACATCACCTAATTGTCCACGCTTGTCCACCTTCATCTTATCTCGTAATTCCATCAGTCATATTCCCAAGTGTTTTTTCAGACGCATGAACGCTCTAACACAACTTGATTTATCATTTAATCTT

>A_Del11bp_Ins25bp_C

CCATATCTTGAACGACAATCACACTTACTTGATAAAATGTAATGAAAAATTGAGAAAGATGCCTCAGATGCGGGAATGGACAGCATCTATCTCATTACCCGCCAGAGGACAGTTTCTTTGGCGGGTAATGAGATAGAAGAAATAGCAGAGGGCACATCACCTAATTGTCCACGCTTGTCCACCTTCATCTTATCTCGTAATTCCATCAGTCATATTCCCAAGTGTTTTTTCAGACACATGAACGCTCTAACACAACTTGATTTATCATATAATCGT

>A_Del12bp_C

CCATATCTTGAACGACAATCACACTTACTTGATAAAATGTAATGAAAAATTGAGAAAGATGCCTCAGATGCGGGAATGGACAGCAGTTTCTTTGGCGGGTAATGAGATAGAAGAAATAGCAGAGGGCACATCACCTAATTGTCCACGCTTGTCCACCTTCATCTTATCTCGTAATTCCATCAGTCATATTCCCAAGTGTTTTTTCAGACACATGAACGCTCTAACACAACTTGATTTATCATATAATCGT

>A3555_Paralog_C

CAATATCTTGAACGAGAATCACACTTACATGATAAAATGTCATGAAAATTTGAGAAAGATACCTCAGATGCGGGAATGGACAGCTGATCTGGAGGCAGTTTCTTTGGCGGGTAATGAGATAGAAGAAATAGCAGAGGGCACATCACCTAATTGTCCACGCTTGTCCACCTTCATCTTATCTCGTAATTCCATCAGTCATATTCCCAAGTGTTTTTTCAGACACATGAACGCTCTAACACAACTTGATTTATCATATAATCGT

>A3555_Paralog_D

CAATATCTTGAACGAGAATCACACTTACATGATAAAATGTCATGAAAATTTGACAAAGATACCTCAGATGCGGGAATGGACAGCTGATCTGGAGGCCGTTTCTTTGGCGGGTAATGAGATAGAAGAAATAGCAGAGGGCACATCACCTAATTGTCCTCGCTTGTCCACCTTCATCTTATCTCGTAATTCCATCAGTCATATTCCCAAGTGTTTTTTCAGACACATGAATGCTCTAACACTACTTGATTTATCATATAATTAT

>D_Del2bp_D

CAATATCTTGAACGAGAATCACACTTACATGATAAAATGTCATGAAAATTTGACAAAGATACCTCAGATGCGGGAATGGACAGCTGATGGAGGCCGTTTCTTTGGCGGGTAATGAGATAGAAGAAATAGCAGAGGGCACATCACCTAATTGTCCTCGCTTGTCCACCTTCATCTTATCTCGTAATTCCATCAGTCATATTCCCAAGTGTTTTTTCAGACACATGAATGCTCTAACACTACTTGATTTATCATATAATTAT

R1-17

>A3555_Paralog_A

CCATATCTTGAACGACAATCACACTTACTTGATAAAATGTAATGAAAAATTGAGAAAGATGCCTCAGATGCGGGAATGGACAGCTGATCTGGAGGCAGTTTCTTTGGCGGGTAATGAGATAGAAGAAATAGCAGAGGGCACATCACCTAATTGTCCACGCTTGTCCACCTTCATCTTATCTCGTAATTCCATCAGTCATATTCCCAAGTGTTTTTTCAGACGCATGAACGCTCTAACACAACTTGATTTATCATTTAATCTT

>A_Del12bp_C

CCATATCTTGAACGACAATCACACTTACTTGATAAAATGTAATGAAAAATTGAGAAAGATGCCTCAGATGCGGGAATGGACAGCAGTTTCTTTGGCGGGTAATGAGATAGAAGAAATAGCAGAGGGCACATCACCTAATTGTCCACGCTTGTCCACCTTCATCTTATCTCGTAATTCCATCAGTCATATTCCCAAGTGTTTTTTCAGACACATGAACGCTCTAACACAACTTGATTTATCATATAATCGT

>A3555_Paralog_C

CAATATCTTGAACGAGAATCACACTTACATGATAAAATGTCATGAAAATTTGAGAAAGATACCTCAGATGCGGGAATGGACAGCTGATCTGGAGGCAGTTTCTTTGGCGGGTAATGAGATAGAAGAAATAGCAGAGGGCACATCACCTAATTGTCCACGCTTGTCCACCTTCATCTTATCTCGTAATTCCATCAGTCATATTCCCAAGTGTTTTTTCAGACACATGAACGCTCTAACACAACTTGATTTATCATATAATCGT

**A3555 R1 mutants at TS3**

R1-18

>A3555_Paralog_A

TTTAATCTTAGATTAACATCTTTGCCAAAGTCGCTGTCTAAGTTGAGGTCTCTTACTTCTTTAGTGCTCCGTCAATGTTCAAAATTGAAAGATATACCTCCACTGGGAGATCTACAAGCATTGTCAAGATTGGACATTTCAGGTTGTGATTCGCTCCTCAGGGTACCGGAAGGCTTGCAAAATCTAAAAAAGTTGCAATGCCTTAATCTTTCCCGCGATTTGTATTTATCATTGTTACCC

>A_Intact_C

TTTAATCTTAGATTAACATCTTTGCCAAAGTCGCTGTCTAAGTTGAGGTCTCTTACTTCTTTAGTGCTCCGTCAATGTTCAAAATTGAAAGATATACCTCCACTGGGAGATCTACAAGCATTGTCAAGATTGGACATTTCCGGTTGTAATTCGCTCCTCAGGGTACCGGAAGGCTTGCAAAATCTAAAAAAGTTGCAATGGCTTAGTCTTTCCCGCAAGCTGAATTTATCATTAGTACCG

>A3555_Paralog_C

TATAATCGTAGATTAACATCTTTGCCAAAGTCGCTGTCTAAGTTGAGGTCTCTTACTTCTTTAGTGCTCCGCCAATGTTCAAAATTGAAAGATATACCTCCACTGGGAGATCTACAAGCATTGTCAAGATTGGACATTTCCGGTTGTAATTCGCTCCTCAGGGTACCGGAAGGCTTGCAAAATCTAAAAAAGTTGCAATGGCTTAGTCTTTCCCGCAAGCTGAATTTATCATTAGTACCG

>A3555_Paralog_D

TATAATTATGAGTTAACATCTTTGCCAAAGTCTCTGTCTAAGTTGAGGTCTCTTACTTCTTTAGTGCTCCGTGAATGTCGTCAATTGGAATATATACCTCCACTGGGAGATCTACATGCATTGTCAAGATTGGACATTTCAGGTTGTGATTCGCTCCTCAGGGTACCGGAAGGCTTGCAAAATCTAAAAAAGTTGCAATGCCTTAATCTTTCCCGCGATTTGTATTTATCATTGTTACTC

>D_Intact_D

TATAATTATGAGTTAACATCTTTGCCAAAGTCTCTGTCTAAGTTGAGGTCTCTTACTTCTTTAGTGCTCCGTGAATGTCGTCAATTGGAATATATACCTCCACTGGGAGATCTACATGCATTGTCAAGATTGGACATTTCAGGTTGTGATTCGCTCCTCAGGGTACCGGAAGGCTTGCAAAATCTAAAAAAGTTGCAATGCCTTAATCTTTCCCGCGATTTGTATTTATCATTGTTACTC

R1-19

>A3555_Paralog_A

TTTAATCTTAGATTAACATCTTTGCCAAAGTCGCTGTCTAAGTTGAGGTCTCTTACTTCTTTAGTGCTCCGTCAATGTTCAAAATTGAAAGATATACCTCCACTGGGAGATCTACAAGCATTGTCAAGATTGGACATTTCAGGTTGTGATTCGCTCCTCAGGGTACCGGAAGGCTTGCAAAATCTAAAAAAGTTGCAATGCCTTAATCTTTCCCGCGATTTGTATTTATCATTGTTACCC

>A_Intact_C

TTTAATCTTAGATTAACATCTTTGCCAAAGTCGCTGTCTAAGTTGAGGTCTCTTACTTCTTTAGTGCTCCGTCAATGTTCAAAATTGAAAGATATACCTCCACTGGGAGATCTACAAGCATTGTCAAGATTGGACATTTCCGGTTGTAATTCGCTCCTCAGGGTACCGGAAGGCTTGCAAAATCTAAAAAAGTTGCAATGCCTTAATCTTTCCCGCGATTTGTATTTATCATTGTTACTC

>A_Del8bp_C

TTTAATCTTAGATTAACATCTTTGCCAAAGTCGCTGTCTAAGTTGAGGTCTCTTACTTCTTTAGTGCTCCGTCAATGTTCAAAATTGAAAGATATACCTCCATCTACAAGCATTGTCAAGATTGGACATTTCCGGTTGTAATTCGCTCCTCAGGGTACCGGAAGGCTTGCAAAATCTAAAAAAGTTGCAATGGCTTAGTCTTTCCCGCAAGCTGAATTTATCATTAGTACCG

>A_Ins1bp_C

TTTAATCTTAGATTAACATCTTTGCCAAAGTCGCTGTCTAAGTTGAGGTCTCTTACTTCTTTAGTGCTCCGTCAATGTTCAAAATTGAAAGATATACCTCCAACTGGGAGATCTACAAGCATTGTCAAGATTGGACATTTCCGGTTGTAATTCGCTCCTCAGGGTACCGGAAGGCTTGCAAAATCTAAAAAAGTTGCAATGGCTTAGTCTTTCCCGCAAGCTGAATTTATCATTAGTACCG

>A3555_Paralog_C

TATAATCGTAGATTAACATCTTTGCCAAAGTCGCTGTCTAAGTTGAGGTCTCTTACTTCTTTAGTGCTCCGCCAATGTTCAAAATTGAAAGATATACCTCCACTGGGAGATCTACAAGCATTGTCAAGATTGGACATTTCCGGTTGTAATTCGCTCCTCAGGGTACCGGAAGGCTTGCAAAATCTAAAAAAGTTGCAATGGCTTAGTCTTTCCCGCAAGCTGAATTTATCATTAGTACCG

>A3555_Paralog_B

TATAATTATGAGTTAACATCTTTGCCAAAGTCTCTGTCTAAGTTGAGGTCTCTTACTTCTTTAGTGCTCCGTCAATGTTCAAAATTGAAAGATATACCTCCACTGGGAGATCTACAAGCATTGTCAAGATTGGACATTTCAGGTTGTGATTCGCTCCTCAGGGTACCGGAAGGCTTGCAAAATCTAAAAAAGTTGCAATGCCTTAATCTTTCCCGCGATTTGTATTTATCATTGTTACCC

>B_Del7bp_B

TATAATTATGAGTTAACATCTTTGCCAAAGTCTCTGTCTAAGTTGAGGTCTCTTACTTCTTTAGTGCTCCGTCAATGTTCAAAATTGAAAGATATACCTCCAATCTACAAGCATTGTCAAGATTGGACATTTCAGGTTGTGATTCGCTCCTCAGGGTACCGGAAGGCTTGCAAAATCTAAAAAAGTTGCAATGCCTTAATCTTTCCCGCGATTTGTATTTATCATTGTTACCC

R1-20

>A3555_Paralog_C

TATAATCGTAGATTAACATCTTTGCCAAAGTCGCTGTCTAAGTTGAGGTCTCTTACTTCTTTAGTGCTCCGCCAATGTTCAAAATTGAAAGATATACCTCCACTGGGAGATCTACAAGCATTGTCAAGATTGGACATTTCCGGTTGTAATTCGCTCCTCAGGGTACCGGAAGGCTTGCAAAATCTAAAAAAGTTGCAATGGCTTAGTCTTTCCCGCAAGCTGAATTTATCATTAGTACCG

>C_Del2bp_C

TATAATCGTAGATTAACATCTTTGCCAAAGTCGCTGTCTAAGTTGAGGTCTCTTACTTCTTTAGTGCTCCGCCAATGTTCAAAATTGAAAGATATACCTCCTGGGAGATCTACAAGCATTGTCAAGATTGGACATTTCCGGTTGTAATTCGCTCCTCAGGGTACCGGAAGGCTTGCAAAATCTAAAAAAGTTGCAATGGCTTAGTCTTTCCCGCAAGCTGAATTTATCATTAGTACCG

>C_Del5bp_C

TATAATCGTAGATTAACATCTTTGCCAAAGTCGCTGTCTAAGTTGAGGTCTCTTACTTCTTTAGTGCTCCGCCAATGTTCAAAATTGAAAGATATACCTCCAAGATCTACAAGCATTGTCAAGATTGGACATTTCCGGTTGTAATTCGCTCCTCAGGGTACCGGAAGGCTTGCAAAATCTAAAAAAGTTGCAATGGCTTAGTCTTTCCCGCAAGCTGAATTTATCATTAGTACCG

>A3555_Paralog_D

TATAATTATGAGTTAACATCTTTGCCAAAGTCTCTGTCTAAGTTGAGGTCTCTTACTTCTTTAGTGCTCCGTGAATGTCGTCAATTGGAATATATACCTCCACTGGGAGATCTACATGCATTGTCAAGATTGGACATTTCAGGTTGTGATTCGCTCCTCAGGGTACCGGAAGGCTTGCAAAATCTAAAAAAGTTGCAATGCCTTAATCTTTCCCGCGATTTGTATTTATCATTGTTACTC

>D_Del2bp_D

TATAATTATGAGTTAACATCTTTGCCAAAGTCTCTGTCTAAGTTGAGGTCTCTTACTTCTTTAGTGCTCCGTGAATGTCGTCAATTGGAATATATACCTCCTGGGAGATCTACATGCATTGTCAAGATTGGACATTTCAGGTTGTGATTCGCTCCTCAGGGTACCGGAAGGCTTGCAAAATCTAAAAAAGTTGCAATGCCTTAATCTTTCCCGCGATTTGTATTTATCATTGTTACTC

>D_Del3bp_D

TATAATTATGAGTTAACATCTTTGCCAAAGTCTCTGTCTAAGTTGAGGTCTCTTACTTCTTTAGTGCTCCGTGAATGTCGTCAATTGGAATATATACCTCTGGGAGATCTACATGCATTGTCAAGATTGGACATTTCAGGTTGTGATTCGCTCCTCAGGGTACCGGAAGGCTTGCAAAATCTAAAAAAGTTGCAATGCCTTAATCTTTCCCGCGATTTGTATTTATCATTGTTACTC

R1-21

>A3555_Paralog_A

TTTAATCTTAGATTAACATCTTTGCCAAAGTCGCTGTCTAAGTTGAGGTCTCTTACTTCTTTAGTGCTCCGTCAATGTTCAAAATTGAAAGATATACCTCCACTGGGAGATCTACAAGCATTGTCAAGATTGGACATTTCAGGTTGTGATTCGCTCCTCAGGGTACCGGAAGGCTTGCAAAATCTAAAAAAGTTGCAATGCCTTAATCTTTCCCGCGATTTGTATTTATCATTGTTACCC

>A_Ins1bp_A

TTTAATCTTAGATTAACATCTTTGCCAAAGTCGCTGTCTAAGTTGAGGTCTCTTACTTCTTTAGTGCTCCGTCAATGTTCAAAATTGAAAGATATACCTCCAACTGGGAGATCTACAAGCATTGTCAAGATTGGACATTTCAGGTTGTGATTCGCTCCTCAGGGTACCGGAAGGCTTGCAAAATCTAAAAAAGTTGCAATGCCTTAATCTTTCCCGCGATTTGTATTTATCATTGTTACCC

>A3555_Paralog_B

TATAATTATGAGTTAACATCTTTGCCAAAGTCTCTGTCTAAGTTGAGGTCTCTTACTTCTTTAGTGCTCCGTCAATGTTCAAAATTGAAAGATATACCTCCACTGGGAGATCTACAAGCATTGTCAAGATTGGACATTTCAGGTTGTGATTCGCTCCTCAGGGTACCGGAAGGCTTGCAAAATCTAAAAAAGTTGCAATGCCTTAATCTTTCCCGCGATTTGTATTTATCATTGTTACCC

>B_Del3bp_B

TATAATTATGAGTTAACATCTTTGCCAAAGTCTCTGTCTAAGTTGAGGTCTCTTACTTCTTTAGTGCTCCGTCAATGTTCAAAATTGAAAGATATACCTCCAGGAGATCTACAAGCATTGTCAAGATTGGACATTTCAGGTTGTGATTCGCTCCTCAGGGTACCGGAAGGCTTGCAAAATCTAAAAAAGTTGCAATGCCTTAATCTTTCCCGCGATTTGTATTTATCATTGTTACCC

>B_Del7bp_B

TATAATTATGAGTTAACATCTTTGCCAAAGTCTCTGTCTAAGTTGAGGTCTCTTACTTCTTTAGTGCTCCGTCAATGTTCAAAATTGAAAGATATACCTCCAATCTACAAGCATTGTCAAGATTGGACATTTCAGGTTGTGATTCGCTCCTCAGGGTACCGGAAGGCTTGCAAAATCTAAAAAAGTTGCAATGCCTTAATCTTTCCCGCGATTTGTATTTATCATTGTTACCC

>B_Del8bp_B

TATAATTATGAGTTAACATCTTTGCCAAAGTCTCTGTCTAAGTTGAGGTCTCTTACTTCTTTAGTGCTCCGTCAATGTTCAAAATTGAAAAGATATACCTCCATCTACAAGCATTGTCAAGATTGGACATTTCAGGTTGTGATTCGCTCCTCAGGGTACCGGAAGGCTTGCAAAATCTAAAAAAGTTGCAATGCCTTAATCTTTCCCGCGATTTGTATTTATCATTGTTACCC

>B_Del13bp_B

TATAATTATGAGTTAACATCTTTGCCAAAGTCTCTGTCTAAGTTGAGGTCTCTTACTTCTTTAGTGCTCCGTCAATGTTCAAAATTGAAAGATAAGATCTACAAGCATTGTCAAGATTGGACATTTCAGGTTGTGATTCGCTCCTCAGGGTACCGGAAGGCTTGCAAAATCTAAAAAAGTTGCAATGCCTTAATCTTTCCCGCGATTTGTATTTATCATTGTTACCC

>A3555_Paralog_D

TATAATTATGAGTTAACATCTTTGCCAAAGTCTCTGTCTAAGTTGAGGTCTCTTACTTCTTTAGTGCTCCGTGAATGTCGTCAATTGGAATATATACCTCCACTGGGAGATCTACATGCATTGTCAAGATTGGACATTTCAGGTTGTGATTCGCTCCTCAGGGTACCGGAAGGCTTGCAAAATCTAAAAAAGTTGCAATGCCTTAATCTTTCCCGCGATTTGTATTTATCATTGTTACTC

>D_Ins1bp_D

TATAATTATGAGTTAACATCTTTGCCAAAGTCTCTGTCTAAGTTGAGGTCTCTTACTTCTTTAGTGCTCCGTGAATGTCGTCAATTGGAATATATACCTCCATCTGGGAGATCTACATGCATTGTCAAGATTGGACATTTCAGGTTGTGATTCGCTCCTCAGGGTACCGGAAGGCTTGCAAAATCTAAAAAAGTTGCAATGCCTTAATCTTTCCCGCGATTTGTATTTATCATTGTTACTC

R1-22

>A3555_Paralog_B

TATAATTATGAGTTAACATCTTTGCCAAAGTCTCTGTCTAAGTTGAGGTCTCTTACTTCTTTAGTGCTCCGTCAATGTTCAAAATTGAAAGATATACCTCCACTGGGAGATCTACAAGCATTGTCAAGATTGGACATTTCAGGTTGTGATTCGCTCCTCAGGGTACCGGAAGGCTTGCAAAATCTAAAAAAGTTGCAATGCCTTAATCTTTCCCGCGATTTGTATTTATCATTGTTACCC

>B_Del4bp_B

TATAATTATGAGTTAACATCTTTGCCAAAGTCTCTGTCTAAGTTGAGGTCTCTTACTTCTTTAGTGCTCCGTCAATGTTCAAAATTGAAAGATATACCTCCAGAGATCTACAAGCATTGTCAAGATTGGACATTTCAGGTTGTGATTCGCTCCTCAGGGTACCGGAAGGCTTGCAAAATCTAAAAAAGTTGCAATGCCTTAATCTTTCCCGCGATTTGTATTTATCATTGTTACCC

>B_Del51bp_B

TATAATTATGAGTTAACATCTTTGCCAAAGTCTCTGTCTAAGTTGAGGTCTCTTACTTCTTCTACATGCATTGTCAAGATTGGACATTTCAGGTTGTGATTCGCTCCTCAGGGTACCGGAAGGCTTGCAAAATCTAAAAAAGTTGCAATGCCTTAATCTTTCCCGCGATTTGTATTTATCATTGTTACTC

>A3555_Paralog_C

TATAATCGTAGATTAACATCTTTGCCAAAGTCGCTGTCTAAGTTGAGGTCTCTTACTTCTTTAGTGCTCCGCCAATGTTCAAAATTGAAAGATATACCTCCACTGGGAGATCTACAAGCATTGTCAAGATTGGACATTTCCGGTTGTAATTCGCTCCTCAGGGTACCGGAAGGCTTGCAAAATCTAAAAAAGTTGCAATGGCTTAGTCTTTCCCGCAAGCTGAATTTATCATTAGTACCG

>C_Del5bp_C

TATAATCGTAGATTAACATCTTTGCCAAAGTCGCTGTCTAAGTTGAGGTCTCTTACTTCTTTAGTGCTCCGCCAATGTTCAAAATTGAAAGATATACCTCCAAGATCTACAAGCATTGTCAAGATTGGACATTTCCGGTTGTAATTCGCTCCTCAGGGTACCGGAAGGCTTGCAAAATCTAAAAAAGTTGCAATGGCTTAGTCTTTCCCGCAAGCTGAATTTATCATTAGTACCG

>A3555_Paralog_D

TATAATTATGAGTTAACATCTTTGCCAAAGTCTCTGTCTAAGTTGAGGTCTCTTACTTCTTTAGTGCTCCGTGAATGTCGTCAATTGGAATATATACCTCCACTGGGAGATCTACATGCATTGTCAAGATTGGACATTTCAGGTTGTGATTCGCTCCTCAGGGTACCGGAAGGCTTGCAAAATCTAAAAAAGTTGCAATGCCTTAATCTTTCCCGCGATTTGTATTTATCATTGTTACTC

>D_Ins1bp_D

TATAATTATGAGTTAACATCTTTGCCAAAGTCTCTGTCTAAGTTGAGGTCTCTTACTTCTTTAGTGCTCCGTGAATGTCGTCAATTGGAATATATACCTCCAACTGGGAGATCTACATGCATTGTCAAGATTGGACATTTCAGGTTGTGATTCGCTCCTCAGGGTACCGGAAGGCTTGCAAAATCTAAAAAAGTTGCAATGCCTTAATCTTTCCCGCGATTTGTATTTATCATTGTTACTC

>D_Del5bp_D

TATAATTATGAGTTAACATCTTTGCCAAAGTCTCTGTCTAAGTTGAGGTCTCTTACTTCTTTAGTGCTCCGTGAATGTCGTCAATTGGAATATATACCTGGGAGATCTACATGCATTGTCAAGATTGGACATTTCAGGTTGTGATTCGCTCCTCAGGGTACCGGAAGGCTTGCAAAATCTAAAAAAGTTGCAATGCCTTAATCTTTCCCGCGATTTGTATTTATCATTGTTACTC

R1-23

>A3555_Paralog_A

TTTAATCTTAGATTAACATCTTTGCCAAAGTCGCTGTCTAAGTTGAGGTCTCTTACTTCTTTAGTGCTCCGTCAATGTTCAAAATTGAAAGATATACCTCCACTGGGAGATCTACAAGCATTGTCAAGATTGGACATTTCAGGTTGTGATTCGCTCCTCAGGGTACCGGAAGGCTTGCAAAATCTAAAAAAGTTGCAATGCCTTAATCTTTCCCGCGATTTGTATTTATCATTGTTACCC

>A_Ins1bp_A

TTTAATCTTAGATTAACATCTTTGCCAAAGTCGCTGTCTAAGTTGAGGTCTCTTACTTCTTTAGTGCTCCGTCAATGTTCAAAATTGAAAGATATACCTCCAGCTGGGAGATCTACAAGCATTGTCAAGATTGGACATTTCAGGTTGTGATTCGCTCCTCAGGGTACCGGAAGGCTTGCAAAATCTAAAAAAGTTGCAATGCCTTAATCTTTCCCGCGATTTGTATTTATCATTGTTACCC

>A_Del1bp_A

TTTAATCTTAGATTAACATCTTTGCCAAAGTCGCTGTCTAAGTTGAGGTCTCTTACTTCTTTAGTGCTCCGTCAATGTTCAAAATTGAAAGATATACCTCCATGGGAGATCTACAAGCATTGTCAAGATTGGACATTTCAGGTTGTGATTCGCTCCTCAGGGTACCGGAAGGCTTGCAAAATCTAAAAAAGTTGCAATGCCTTAATCTTTCCCGCGATTTGTATTTATCATTGTTACCC

>A_Del12bp_A

TTTAATCTTAGATTAACATCTTTGCCAAAGTCGCTGTCTAAGTTGAGGTCTCTTACTTCTTTAGTGCTCCGTCAATGTTCAAAATTGAAAGATATACCTCTACAAGCATTGTCAAGATTGGACATTTCAGGTTGTGATTCGCTCCTCAGGGTACCGGAAGGCTTGCAAAATCTAAAAAAGTTGCAATGCCTTAATCTTTCCCGCGATTTGTATTTATCATTGTTACCC

>A3555_Paralog_C

TATAATCGTAGATTAACATCTTTGCCAAAGTCGCTGTCTAAGTTGAGGTCTCTTACTTCTTTAGTGCTCCGCCAATGTTCAAAATTGAAAGATATACCTCCACTGGGAGATCTACAAGCATTGTCAAGATTGGACATTTCCGGTTGTAATTCGCTCCTCAGGGTACCGGAAGGCTTGCAAAATCTAAAAAAGTTGCAATGGCTTAGTCTTTCCCGCAAGCTGAATTTATCATTAGTACCG

C_Ins1bp_C

TATAATCGTAGATTAACATCTTTGCCAAAGTCGCTGTCTAAGTTGAGGTCTCTTACTTCTTTAGTGCTCCGCCAATGTTCAAAATTGAAAGATATACCTCCAACTGGGAGATCTACAAGCATTGTCAAGATTGGACATTTCCGGTTGTAATTCGCTCCTCAGGGTACCGGAAGGCTTGCAAAATCTAAAAAAGTTGCAATGGCTTAGTCTTTCCCGCAAGCTGAATTTATCATTAGTACCG

R1-24

A3555_Paralog_A

TTTAATCTTAGATTAACATCTTTGCCAAAGTCGCTGTCTAAGTTGAGGTCTCTTACTTCTTTAGTGCTCCGTCAATGTTCAAAATTGAAAGATATACCTCCACTGGGAGATCTACAAGCATTGTCAAGATTGGACATTTCAGGTTGTGATTCGCTCCTCAGGGTACCGGAAGGCTTGCAAAATCTAAAAAAGTTGCAATGCCTTAATCTTTCCCGCGATTTGTATTTATCATTGTTACCC

>A_Del4bp_A

TTTAATCTTAGATTAACATCTTTGCCAAAGTCGCTGTCTAAGTTGAGGTCTCTTACTTCTTTAGTGCTCCGTCAATGTTCAAAATTGAAAGATATACCTCTGGGAGATCTACAAGCATTGTCAAGATTGGACATTTCAGGTTGTGATTCGCTCCTCAGGGTACCGGAAGGCTTGCAAAATCTAAAAAAGTTGCAATGCCTTAATCTTTCCCGCGATTTGTATTTATCATTGTTACCC

>A_Del8bp_A

TTTAATCTTAGATTAACATCTTTGCCAAAGTCGCTGTCTAAGTTGAGGTCTCTTACTTCTTTAGTGCTCCGTCAATGTTCAAAATTGAAAGATATACCTCGATCTACAAGCATTGTCAAGATTGGACATTTCAGGTTGTGATTCGCTCCTCAGGGTACCGGAAGGCTTGCAAAATCTAAAAAAGTTGCAATGCCTTAATCTTTCCCGCGATTTGTATTTATCATTGTTACCC

>A3555_Paralog_B

TATAATTATGAGTTAACATCTTTGCCAAAGTCTCTGTCTAAGTTGAGGTCTCTTACTTCTTTAGTGCTCCGTCAATGTTCAAAATTGAAAGATATACCTCCACTGGGAGATCTACAAGCATTGTCAAGATTGGACATTTCAGGTTGTGATTCGCTCCTCAGGGTACCGGAAGGCTTGCAAAATCTAAAAAAGTTGCAATGCCTTAATCTTTCCCGCGATTTGTATTTATCATTGTTACCC

>B_Del2bp_B

TATAATTATGAGTTAACATCTTTGCCAAAGTCTCTGTCTAAGTTGAGGTCTCTTACTTCTTTAGTGCTCCGTCAATGTTCAAAATTGAAAGATATACCTCCTGGGAGATCTACAAGCATTGTCAAGATTGGACATTTCAGGTTGTGATTCGCTCCTCAGGGTACCGGAAGGCTTGCAAAATCTAAAAAAGTTGCAATGCCTTAATCTTTCCCGCGATTTGTATTTATCATTGTTACCC

>A3555_Paralog_C

TATAATCGTAGATTAACATCTTTGCCAAAGTCGCTGTCTAAGTTGAGGTCTCTTACTTCTTTAGTGCTCCGCCAATGTTCAAAATTGAAAGATATACCTCCACTGGGAGATCTACAAGCATTGTCAAGATTGGACATTTCCGGTTGTAATTCGCTCCTCAGGGTACCGGAAGGCTTGCAAAATCTAAAAAAGTTGCAATGGCTTAGTCTTTCCCGCAAGCTGAATTTATCATTAGTACCG

>C_Ins1bp_C

TATAATCGTAGATTAACATCTTTGCCAAAGTCGCTGTCTAAGTTGAGGTCTCTTACTTCTTTAGTGCTCCGCCAATGTTCAAAATTGAAAGATATACCTCCAACTGGGAGATCTACAAGCATTGTCAAGATTGGACATTTCCGGTTGTAATTCGCTCCTCAGGGTACCGGAAGGCTTGCAAAATCTAAAAAAGTTGCAATGGCTTAGTCTTTCCCGCAAGCTGAATTTATCATTAGTACCG

>A3555_Paralog_D

TATAATTATGAGTTAACATCTTTGCCAAAGTCTCTGTCTAAGTTGAGGTCTCTTACTTCTTTAGTGCTCCGTGAATGTCGTCAATTGGAATATATACCTCCACTGGGAGATCTACATGCATTGTCAAGATTGGACATTTCAGGTTGTGATTCGCTCCTCAGGGTACCGGAAGGCTTGCAAAATCTAAAAAAGTTGCAATGCCTTAATCTTTCCCGCGATTTGTATTTATCATTGTTACTC

>D_Ins1bp_D

TATAATTATGAGTTAACATCTTTGCCAAAGTCTCTGTCTAAGTTGAGGTCTCTTACTTCTTTAGTGCTCCGTGAATGTCGTCAATTGGAATATATACCTCCAACTGGGAGATCTACATGCATTGTCAAGATTGGACATTTCAGGTTGTGATTCGCTCCTCAGGGTACCGGAAGGCTTGCAAAATCTAAAAAAGTTGCAATGCCTTAATCTTTCCCGCGATTTGTATTTATCATTGTTACTC

>D_Del3bp_D

TATAATTATGAGTTAACATCTTTGCCAAAGTCTCTGTCTAAGTTGAGGTCTCTTACTTCTTTAGTGCTCCGTGAATGTCGTCAATTGGAATATATACCTCCAGGAGATCTACATGCATTGTCAAGATTGGACATTTCAGGTTGTGATTCGCTCCTCAGGGTACCGGAAGGCTTGCAAAATCTAAAAAAGTTGCAATGCCTTAATCTTTCCCGCGATTTGTATTTATCATTGTTACTC

>D_Del12bp_D

TATAATTATGAGTTAACATCTTTGCCAAAGTCTCTGTCTAAGTTGAGGTCTCTTACTTCTTTAGTGCTCCGTGAATGTCGTCAATTGGAACTGGGAGATCTACATGCATTGTCAAGATTGGACATTTCAGGTTGTGATTCGCTCCTCAGGGTACCGGAAGGCTTGCAAAATCTAAAAAAGTTGCAATGCCTTAATCTTTCCCGCGATTTGTATTTATCATTGTTACTC

**AG3931 R1 mutants at TS1**

R1-25

>AG3931_Paralog_B

GGGTCGACTAATGAATTGGATGAGCCTTCTGAATTGGATATTGAGTTTGATGTATTATTGATACAGAAGCCTTGGGGGTTGCGAGATAAAAATGTGAAGAAGATGTGGGATCTTCTGGAGGATGAGGAAGTCTTCATTATTGGCATAGATGGAATGGGGGGAGTTGGAAAAACATTCATGGCAACTCATATCAAGAATGAGATTAAAAGAAAGGGGACTTTCAAGGATGTCTTCTGGGTCACTGTTTCCGATGATTTCACCACTTTCAAA

>B_Intact_B

GGGTCGACTAATGAATTGGATGAGCCTTCTGAATTGGATATTGAGTTTGATGTATTATTGATACAGAAGCCTTGGGGGTTGCGAGATAAAAATGTGAAGAAGATGTGGGATCTTCTGGAGGATGAGGAAGTCTTCATTATTGGCATAGATGGAATGGGGGGAGTTGGAAAAACATTCATGGCAACTCATATCAAGAATGAGATTAAAAGAAAGGGGACTTTCAAGGATGTCTTCTGGGTCACTGTTTCCGATGATTTCACCACTTTCAAA

>AG3931_Paralog_C

GGGTCGACTAATGAATTCCCCAAGCCTTCTGAATTGGGTGATGAGTTTTTTTATTTATCGATAGAGAAGCGTTGGGCGTCGCGAGATAAAAAAGTGGAGAACATGTGGGATCTTCTGGAGGATGAGGAAGTCTTCATTATTGGCATAGATGGAATGGGGGGAGTTGGAAAAACATTCATGGCAACTCATATCAAGAATGAGATTAAAAGAAAGGGGACTTTCAAGGATGTCTTCTGGGTCACTGTTTCCCATGATTTCACCAATTTCAAA

>C_Intact_C

GGGTCGACTAATGAATTCCCCAAGCCTTCTGAATTGGGTGATGAGTTTTTTTATTTATCGATAGAGAAGCGTTGGGCGTCGCGAGATAAAAAAGTGGAGAACATGTGGGATCTTCTGGAGGATGAGGAAGTCTTCATTATTGGCATAGATGGAATGGGGGGAGTTGGAAAAACATTCATGGCAACTCATATCAAGAATGAGATTAAAAGAAAGGGGACTTTCAAGGATGTCTTCTGGGTCACTGTTTCCCATGATTTCACCAATTTCAAA

R1-26

>AG3931_Paralog_A

GGGTCGACTAATGAATTGCCCAAGCCTTCTGAATTGCATGCTAAGTTTATTTATTTATTGATAGAGAAGCTTTGGGAGTTGCGAGATGAAAATGTGAAGAAGATGTGGGATCTTCTGGAGGATGAGGAAGTCTTCATTATTGGCATAGATGGAATGGGGGGAGTTGGAAAAACATTCATGGCAACTCATTTCAAGAATGAGATTAAAAGAAAGGGGACTTTCAAGGATGTCTTCTGGGTCACTGTTTCCCATGATTTCACCATTTTCAAA

>A_Del1bp_A

GGGTCGACTAATGAATTGCCCAAGCCTTCTGAATTGCATGCTAAGTTTATTTATTTATTGATAGAGAAGCTTTGGGAGTTGTGAGATGAAAATGTGAAGAAGATGTGGGATCTTCTGGAGATGAGGAAGTCTTCATTATTGGCATAGATGGAATGGGGGGAGTTGGAAAAACATTCATGGCAACTCATTTCAAGAATGAGATTAAAAGAAAGGGGACTTTCAAGGATGTCTTCTGGGTCACTGTTTCCCATGATTTCACCATTTTCAAA

>AG3931_Paralog_C

GGGTCGACTAATGAATTCCCCAAGCCTTCTGAATTGGGTGATGAGTTTTTTTATTTATCGATAGAGAAGCGTTGGGCGTCGCGAGATAAAAAAGTGGAGAACATGTGGGATCTTCTGGAGGATGAGGAAGTCTTCATTATTGGCATAGATGGAATGGGGGGAGTTGGAAAAACATTCATGGCAACTCATATCAAGAATGAGATTAAAAGAAAGGGGACTTTCAAGGATGTCTTCTGGGTCACTGTTTCCCATGATTTCACCAATTTCAAA

>C_Del6bp_C

GGGTCGACTAATGAATTCCCCAAGCCTTCTGAATTGGGTGATGAGTTTTTTTATTTATCGATAGAGAAGCGTTGGGCGTCGCGAGATAAAAAAGTGGAGAACATGTGGGATCTTCTGGAGGAAGTCTTCATTATTGGCATAGATGGAATGGGGGGAGTTGGAAAAACATTCATGGCAACTCATATCAAGAATGAGATTAAAAGAAAGGGGACTTTCAAGGATGTCTTCTGGGTCACTGTTTCCCATGATTTCACCAATTTCAAA

R1-27

>AG3931_Paralog_A

GGGTCGACTAATGAATTGCCCAAGCCTTCTGAATTGCATGCTAAGTTTATTTATTTATTGATAGAGAAGCTTTGGGAGTTGCGAGATGAAAATGTGAAGAAGATGTGGGATCTTCTGGAGGATGAGGAAGTCTTCATTATTGGCATAGATGGAATGGGGGGAGTTGGAAAAACATTCATGGCAACTCATTTCAAGAATGAGATTAAAAGAAAGGGGACTTTCAAGGATGTCTTCTGGGTCACTGTTTCCCATGATTTCACCATTTTCAAA

>A_Del1bp_A

GGGTCGACTAATGAATTGCCCAAGCCTTCTGAATTGCATGCTAAGTTTATTTATTTATTGATAGAGAAGCTTTGGGAGTTGTGAGATGAAAATGTGAAGAAGATGTGGGATCTTCTGGAGATGAGGAAGTCTTCATTATTGGCATAGATGGAATGGGGGGAGTTGGAAAAACATTCATGGCAACTCATTTCAAGAATGAGATTAAAAGAAAGGGGACTTTCAAGGATGTCTTCTGGGTCACTGTTTCCCATGATTTCACCATTTTCAAA

>A_Del3bp_A

GGGTCGACTAATGAATTGCCCAAGCCTTCTGAATTGCATGCTAAGTTTATTTATTTATTGATAGAGAAGCTTTGGGAGTTGCGAGATGAAAATGTGAAGAAGATGTGGGATCTTCTGGATGAGGAAGTCTTCATTATTGGCATAGATGGAATGGGGGGAGTTGGAAAAACATTCATGGCAACTCATTTCAAGAATGAGATTAAAAGAAAGGGGACTTTCAAGGATGTCTTCTGGGTCACTGTTTCCCATGATTTCACCATTTTCAAA

>AG3931_Paralog_B

GGGTCGACTAATGAATTGGATGAGCCTTCTGAATTGGATATTGAGTTTGATGTATTATTGATACAGAAGCCTTGGGGGTTGCGAGATAAAAATGTGAAGAAGATGTGGGATCTTCTGGAGGATGAGGAAGTCTTCATTATTGGCATAGATGGAATGGGGGGAGTTGGAAAAACATTCATGGCAACTCATATCAAGAATGAGATTAAAAGAAAGGGGACTTTCAAGGATGTCTTCTGGGTCACTGTTTCCGATGATTTCACCACTTTCAAA

>B_Del1bp_B

GGGTCGACTAATGAATTGGATGAGCCTTCTGAATTGGATATTGAGTTTGATGTATTATTGATACAGAAGCCTTGGGGGTTGCGAGATAAAAATGTGAAGAAGATGTGGGATCTTCTGGAGATGAGGAAGTCTTCATTATTGGCATAGATGGAATGGGGGGAGTTGGAAAAACATTCATGGCAACTCATATCAAGAATGAGATTAAAAGAAAGGGGACTTTCAAGGATGTCTTCTGGGTCACTGTTTCCGATGATTTCACCACTTTCAAA

>B_Del3bp_B

GGGTCGACTAATGAATTGCCCAAGCCTTCTGAATTACATGCTAAGTTTATTTATTTATTGATAGAGAAGCTTTGGGAGTTGCGAGATGAAAATGTGAAGAAGATGTGGGATCTTCTGGAGGGAAGTCTTCATTATTGGCATAGATGGAATGGGGGGAGTTGGAAAAACATTCATGGCAACTCATATCAAGAATGAGATTAAAAGAAAGGGGACTTTCAAGGATGTCTTCTGGGTCACTGTTTCCGATGATTTCACCACTTTCAAA

>AG3931_Paralog_C

GGGTCGACTAATGAATTCCCCAAGCCTTCTGAATTGGGTGATGAGTTTTTTTATTTATCGATAGAGAAGCGTTGGGCGTCGCGAGATAAAAAAGTGGAGAACATGTGGGATCTTCTGGAGGATGAGGAAGTCTTCATTATTGGCATAGATGGAATGGGGGGAGTTGGAAAAACATTCATGGCAACTCATATCAAGAATGAGATTAAAAGAAAGGGGACTTTCAAGGATGTCTTCTGGGTCACTGTTTCCCATGATTTCACCAATTTCAAA

>C_Del2bp_C

GGGTCGACTAATGAATTCCCCAAGCCTTCTGAATTGGGTGATGAGTTTTTTTATTTATCGATAGAGAAGCGTTGGGCGTCGCGAGATAAAAAAGTGGAGAACATGTGGGATCTTCTGGAGGATGGAAGTCTTCATTATTGGCATAGATGGAATGGGGGGAGTTGGAAAAACATTCATGGCAACTCATATCAAGAATGAGATTAAAAGAAAGGGGACTTTCAAGGATGTCTTCTGGGTCACTGTTTCCCATGATTTCACCAATTTCAAA

>C_Del3bp_C

GGGTCGACTAATGAATTCCCCAAGCCTTCTGAATTGGGTGATGAGTTTTTTTATTTATCGATAGAGAAGCGTTGGGCGTCGCGAGATAAAAAAGTGGAGAACATGTGGGATCTTCTGGATGAGGAAGTCTTCATTATTGGCATAGATGGAATGGGGGGAGTTGGAAAAACATTCATGGCAACTCATATCAAGAATGAGATTAAAAGAAAGGGGACTTTCAAGGATGTCTTCTGGGTCACTGTTTCCCATGATTTCACCAATTTCAAA

R1-28

>AG3931_Paralog_A

GGGTCGACTAATGAATTGCCCAAGCCTTCTGAATTGCATGCTAAGTTTATTTATTTATTGATAGAGAAGCTTTGGGAGTTGCGAGATGAAAATGTGAAGAAGATGTGGGATCTTCTGGAGGATGAGGAAGTCTTCATTATTGGCATAGATGGAATGGGGGGAGTTGGAAAAACATTCATGGCAACTCATTTCAAGAATGAGATTAAAAGAAAGGGGACTTTCAAGGATGTCTTCTGGGTCACTGTTTCCCATGATTTCACCATTTTCAAA

>A_Del1bp_A

GGGTCGACTAATGAATTGCCCAAGCCTTCTGAATTGCATGCTAAGTTTATTTATTTATTGATAGAGAAGCTTTGGGAGTTGCGAGATGAAAATGTGAAGAAGATGTGGGATCTTCTGGAGATGAGGAAGTCTTCATTATTGGCATAGATGGAATGGGGGGAGTTGGAAAAACATTCATGCCAACTCATTTCAAGAATGAGATTAAAAGAAAGGGGACTTTCAAGGATGTCTTCTGGGTCACTGTTTCCCATGATTTCACCATTTTCAAA

>A_Del2bp_A

GGGTCGACTAATGAATTGCCCAAGCCTTCTGAATTGCATGCTAAGTTTATTTATTTATTGATAGAGAAGCTTTGGGAGTTGCGAGATGAAAATGTGAAGAAGATGTGGGATCTTCTGGAATGAGGAAGTCTTCATTATTGGCATAGATGGAATGGGGGGAGTTGGAAAAACATTCATGGCAACTCATTTCAAGAATGAGATTAAAAGAAAGGGGACTTTCAAGGATGTCTTCTGGGTCACTGTTTCCCATGATTTCACCATTTTCAAA

R1-29

>AG3931_Paralog_A

GGGTCGACTAATGAATTGCCCAAGCCTTCTGAATTGCATGCTAAGTTTATTTATTTATTGATAGAGAAGCTTTGGGAGTTGCGAGATGAAAATGTGAAGAAGATGTGGGATCTTCTGGAGGATGAGGAAGTCTTCATTATTGGCATAGATGGAATGGGGGGAGTTGGAAAAACATTCATGGCAACTCATTTCAAGAATGAGATTAAAAGAAAGGGGACTTTCAAGGATGTCTTCTGGGTCACTGTTTCCCATGATTTCACCATTTTCAAA

>A_Del1bp_A

GGGTCGACTAATGAATTGCCCAAGCCTTCTGAATTGCATGCTAAGTTTATTTATTTATTGATAGAGAAGCTTTGGGAGTTGCGAGATGAAAATGTGAAGAAGATGTGGGATCTTCTGGAGGTGAGGAAGTCTTCATTATTGGCATAGATGGAATGGGGGGAGTTGGAAAAACATTCATGGCAACTCATTTCAAGAATGAGATTAAAAGAAAGGGGACTTTCAAGGATGTCTTCTGGGTCACTGTTTCCCATGATTTCACCATTTTCAAA

>A_Del3bp_A

GGGTCGACTAATGAATTGCCCAAGCCTTCTGAATTGCATGCTAAGTTTATTTATTTATTGATAGAGAAGCTTTGGGAGTTGCGAGATGAAAATGTGAAGAAGATGTGGGATCTTCTGGATGAGGAAGTCTTCATTATTGGCATAGATGGAATGGGGGGAGTTGGAAAAACATTCATGGCAACTCATTTCAAGAATGAGATTAAAAGAAAGGGGACTTTCAAGGATGTCTTCTGGGTCACTGTTTCCCATGATTTCACCATTTTCAAA

>A_Del4bp_A

GGGTCGACTAATGAATTGCCCAAGCCTTCTGAATTGCATGCTAAGTTTATTTATTTATTGATAGAGAAGCTTTGGGAGTTGCGAGATGAAAATGTGAAGAAGATGTGGGATCTTCTGGAGAGGAAGTCTTCATTATTGGCATAGATGGAATGGGGGGAGTTGGAAAAACATTCATGGCAACTCATTTCAAGAATGAGATTAAAAGAAAGGGGACTTTCAAGGATGTCTTCTGGGTCACTGTTTCCCATGATTTCACCATTTTCAAA

>AG3931_Paralog_C

GGGTCGACTAATGAATTCCCCAAGCCTTCTGAATTGGGTGATGAGTTTTTTTATTTATCGATAGAGAAGCGTTGGGCGTCGCGAGATAAAAAAGTGGAGAACATGTGGGATCTTCTGGAGGATGAGGAAGTCTTCATTATTGGCATAGATGGAATGGGGGGAGTTGGAAAAACATTCATGGCAACTCATATCAAGAATGAGATTAAAAGAAAGGGGACTTTCAAGGATGTCTTCTGGGTCACTGTTTCCCATGATTTCACCAATTTCAAA

>C_Ins1bp_B

GGGTCGACTAATGAATTCCCCAAGCCTTCTGAATTGGGTGATGAGTTTTTTTATTTATCGATAGAGAAGCGTTGGGCGTCGCGAGATAAAAAAGTGGAGAACATGTGGGATCTTCTGGAGGGATGAGGAAGTCTTCATTATTGGCATAGATGGAATGGGGGGAGTTGGAAAAACATTCATGGCAACTCATATCAAGAATGAGATTAAAAGAAAGGGGACTTTCAAGGATGTCTTCTGGGTCACTGTTTCCGATGATTTCACCACTTTCAAA

>C_Del30bp_B

GGGTCGACTAATGAATTCCCCAAGCCTTCTGAATTGGGTGATGAGTTTTTTTATTTATCGATAGAGAAGCGTTGGGCGTCGCGAGATAAAAAAGTGGAGAACATGTGGGATCTTCTGGAGGGAATGGGGGGAGTTGGAAAAACATTCATGGCAACTCATATCAAGAATGAGATTAAAAGAAAGGGGACTTTCAAGGATGTCTTCTGGGTCACTGTTTCCGATGATTTCACCACTTTCAAA

>AG3931_Paralog_B

GGGTCGACTAATGAATTGGATGAGCCTTCTGAATTGGATATTGAGTTTGATGTATTATTGATACAGAAGCCTTGGGGGTTGCGAGATAAAAATGTGAAGAAGATGTGGGATCTTCTGGAGGATGAGGAAGTCTTCATTATTGGCATAGATGGAATGGGGGGAGTTGGAAAAACATTCATGGCAACTCATATCAAGAATGAGATTAAAAGAAAGGGGACTTTCAAGGATGTCTTCTGGGTCACTGTTTCCGATGATTTCACCACTTTCAAA

R1-30

>AG3931_Paralog_B

GGGTCGACTAATGAATTGGATGAGCCTTCTGAATTGGATATTGAGTTTGATGTATTATTGATACAGAAGCCTTGGGGGTTGCGAGATAAAAATGTGAAGAAGATGTGGGATCTTCTGGAGGATGAGGAAGTCTTCATTATTGGCATAGATGGAATGGGGGGAGTTGGAAAAACATTCATGGCAACTCATATCAAGAATGAGATTAAAAGAAAGGGGACTTTCAAGGATGTCTTCTGGGTCACTGTTTCCGATGATTTCACCACTTTCAAA

>B_Del3bp_B

GGGTCGACTAATGAATTGGATGAGCCTTCTGAATTGGATATTGAGTTTGATGTATTATTGATACAGAAGCCTTGGGGGTTGCGAGATAAAAATGTGAAGAAGATGTGGGATCTTCTGGATGAGGAAGTCTTCATTATTGGCATAGATGGAATGGGGGGAGTTGGAAAAACATTCATGGCAACTCATATCAAGAATGAGATTAAAAGAAAGGGGACTTTCAAGGATGTCTTCTGGGTCACTGTTTCCGATGATTTCACCACTTTCAAA

>AG3931_Paralog_C

GGGTCGACTAATGAATTCCCCAAGCCTTCTGAATTGGGTGATGAGTTTTTTTATTTATCGATAGAGAAGCGTTGGGCGTCGCGAGATAAAAAAGTGGAGAACATGTGGGATCTTCTGGAGGATGAGGAAGTCTTCATTATTGGCATAGATGGAATGGGGGGAGTTGGAAAAACATTCATGGCAACTCATATCAAGAATGAGATTAAAAGAAAGGGGACTTTCAAGGATGTCTTCTGGGTCACTGTTTCCCATGATTTCACCAATTTCAAA

>C_Del4bp_C

GGGTCGACTAATGAATTCCCCAAGCCTTCTGAATTGGGTGATGAGTTTTTTTATTTATCGATAGAGAAGCGTTGGGCGTCGCGAGATAAAAAAGTGGAGAACATGTGGGATCTTCTGATGAGGAAGTCTTCATTATTGGCATAGATGGAATGGGGGGAGTTGGAAAAACATTCATGGCAACTCATATCAAGAATGAGATTAAAAGAAAGGGGACTTTCAAGGATGTCTTCTGGGTCACTGTTTCCCATGATTTCACCAATTTCAAA

R1-31

>AG3931_Paralog_A

GGGTCGACTAATGAATTGCCCAAGCCTTCTGAATTGCATGCTAAGTTTATTTATTTATTGATAGAGAAGCTTTGGGAGTTGCGAGATGAAAATGTGAAGAAGATGTGGGATCTTCTGGAGGATGAGGAAGTCTTCATTATTGGCATAGATGGAATGGGGGGAGTTGGAAAAACATTCATGGCAACTCATTTCAAGAATGAGATTAAAAGAAAGGGGACTTTCAAGGATGTCTTCTGGGTCACTGTTTCCCATGATTTCACCATTTTCAAA

>A_Del1bp_A

GGGTCGACTAATGAATTGCCCAAGCCTTCTGAATTGCATGCTAAGTTTATTTATTTATTGATAGAGAAGCTTTGGGAGTTGCGAGATGAAAATGTGAAGAAGATGTGGGATCTTCTGGAGATGAGGAAGTCTTCATTATTGGCATAGATGGAATGGGGGGAGTTGGAAAAACATTCATGGCAACTCATTTCAAGAATGAGATTAAAAGAAAGGGGACTTTCAAGGATGTCTTCTGGGTCACTGTTTCCCATGATTTCACCATTTTCAAA

>AG3931_Paralog_C

GGGTCGACTAATGAATTCCCCAAGCCTTCTGAATTGGGTGATGAGTTTTTTTATTTATCGATAGAGAAGCGTTGGGCGTCGCGAGATAAAAAAGTGGAGAACATGTGGGATCTTCTGGAGGATGAGGAAGTCTTCATTATTGGCATAGATGGAATGGGGGGAGTTGGAAAAACATTCATGGCAACTCATATCAAGAATGAGATTAAAAGAAAGGGGACTTTCAAGGATGTCTTCTGGGTCACTGTTTCCCATGATTTCACCAATTTCAAA

>C_Del4bp_C

GGGTCGACTAATGAATTCCCCAAGCCTTCTGAATTGGGTGATGAGTTTTTTTATTTATCGATAGAGAAGCGTTGGGCGTCGCGAGATAAAAAAGTGGAGAACATGTGGGATCTTCTGATGAGGAAGTCTTCATTATTGGCATAGATGGAATGGGGGGAGTTGGAAAAACATTCATGGCAACTCATATCAAGAATGAGATTAAAAGAAAGGGGACTTTCAAGGATGTCTTCTGGGTCACTGTTTCCCATGATTTCACCAATTTCAAA

R1-32

>AG3931_Paralog_A

GGGTCGACTAATGAATTGCCCAAGCCTTCTGAATTGCATGCTAAGTTTATTTATTTATTGATAGAGAAGCTTTGGGAGTTGCGAGATGAAAATGTGAAGAAGATGTGGGATCTTCTGGAGGATGAGGAAGTCTTCATTATTGGCATAGATGGAATGGGGGGAGTTGGAAAAACATTCATGGCAACTCATTTCAAGAATGAGATTAAAAGAAAGGGGACTTTCAAGGATGTCTTCTGGGTCACTGTTTCCCATGATTTCACCATTTTCAAA

>A_Del1bp_A

GGGTCGACTAATGAATTGCCCAAGCCTTCTGAATTGCATGCTAAGTTTATTTATTTATTGATAGAGAAGCTTTGGGAGTTGCGAGATGAAAATGTGAAGAAGATGTGGGATCTTCTGGAGATGAGGAAGTCTTCATTATTGGCATAGATGGAATGGGGGGAGTTGGAAAAACATTCATGGCAACTCATTTCAAGAATGAGATTAAAAGAAAGGGGACTTTCAAGGATGTCTTCTGGGTCACTGTTTCCCATGATTTCACCATTTTCAAA

>AG3931_Paralog_C

GGGTCGACTAATGAATTCCCCAAGCCTTCTGAATTGGGTGATGAGTTTTTTTATTTATCGATAGAGAAGCGTTGGGCGTCGCGAGATAAAAAAGTGGAGAACATGTGGGATCTTCTGGAGGATGAGGAAGTCTTCATTATTGGCATAGATGGAATGGGGGGAGTTGGAAAAACATTCATGGCAACTCATATCAAGAATGAGATTAAAAGAAAGGGGACTTTCAAGGATGTCTTCTGGGTCACTGTTTCCCATGATTTCACCAATTTCAAA

>C_Del4bp_C

GGGTCGACTAATGAATTCCCCAAGCCTTCTGAATTGGGTGATGAGTTTTTTTATTTATCGATAGAGAAGCGTTGGGCGTCGCGAGATAAAAAAGTGGAGAACATGTGGGATCTTCTGATGAGGAAGTCTTCATTATTGGCATAGATGGAATGGGGGGAGTTGGAAAAACATTCATGGCAACTCATATCAAGAATGAGATTAAAAGAAAGGGGACTTTCAAGGATGTCTTCTGGGTCACTGTTTCCCATGATTTCACCAATTTCAAA

>AG3931_Paralog_D

GGGTCGACTAATGAATTCCCCAAGCCTTCTGAATTGGATGATGAGTATGATGATTTATTGGAAGAGAATCCTTGGGTGTCGCGAGATGAAAATGTGAAGGAGATGTGGGATCTTCTGGAGGATGAGGAAGTCTTCATTATTGGCATAGATGGAATGGGGGGAGTTGGAAAAACATTCATGGCAACTCATATCAAGAATGAGATTAAAAGAAAGGGGACTTTCAAGGATGTCTTCTGGGTCACTGTTTCCGATGATTTCACCACTTTCAAA

>D_Intact_D

GGGTCGACTAATGAATTCCCCAAGCCTTCTGAATTGGATGATGAGTATGATGATTTATTGGAAGAGAATCCTTGGGTGTCGCGAGATGAAAATGTGAAGGAGATGTGGGATCTTCTGGAGGATGAGGAAGTCTTCATTATTGGCATAGATGGAATGGGGGGAGTTGGAAAAACATTCATGGCAACTCATATCAAGAATGAGATTAAAAGAAAGGGGACTTTCAAGGATGTCTTCTGGGTCACTGTTTCCGATGATTTCACCACTTTCAAA

R1-33

>AG3931_Paralog_B

GGGTCGACTAATGAATTGGATGAGCCTTCTGAATTGGATATTGAGTTTGATGTATTATTGATACAGAAGCCTTGGGGGTTGCGAGATAAAAATGTGAAGAAGATGTGGGATCTTCTGGAGGATGAGGAAGTCTTCATTATTGGCATAGATGGAATGGGGGGAGTTGGAAAAACATTCATGGCAACTCATATCAAGAATGAGATTAAAAGAAAGGGGACTTTCAAGGATGTCTTCTGGGTCACTGTTTCCGATGATTTCACCACTTTCAAA

>B_Intact_B

GGGTCGACTAATGAATTGGATGAGCCTTCTGAATTGGATATTGAGTTTGATGTATTATTGATACAGAAGCCTTGGGGGTTGCGAGATAAAAATGTGAAGAAGATGTGGGATCTTCTGGAGGATGAGGAAGTCTTCATTATTGGCATAGATGGAATGGGGGGAGTTGGAAAAACATTCATGGCAACTCATATCAAGAATGAGATTAAAAGAAAGGGGACTTTCAAGGATGTCTTCTGGGTCACTGTTTCCGATGATTTCACCACTTTCAAA

>AG3931_Paralog_C

GGGTCGACTAATGAATTCCCCAAGCCTTCTGAATTGGGTGATGAGTTTTTTTATTTATCGATAGAGAAGCGTTGGGCGTCGCGAGATAAAAAAGTGGAGAACATGTGGGATCTTCTGGAGGATGAGGAAGTCTTCATTATTGGCATAGATGGAATGGGGGGAGTTGGAAAAACATTCATGGCAACTCATATCAAGAATGAGATTAAAAGAAAGGGGACTTTCAAGGATGTCTTCTGGGTCACTGTTTCCCATGATTTCACCAATTTCAAA

>C_Intact_C

GGGTCGACTAATGAATTCCCCAAGCCTTCTGAATTGGGTGATGAGTTTTTTTATTTATCGATAGAGAAGCGTTGGGCGTCGCGAGATAAAAAAGTGGAGAACATGTGGGATCTTCTGGAGGATGAGGAAGTCTTCATTATTGGCATAGATGGAATGGGGGGAGTTGGAAAAACATTCATGGCAACTCATATCAAGAATGAGATTAAAAGAAAGGGGACTTTCAAGGATGTCTTCTGGGTCACTGTTTCCCATGATTTCACCAATTTCAAA

>C_Del4bp_C

GGGTCGACTAATGAATTCCCCAAGCCTTCTGAATTGGGTGATGAGTTTTTTTATTTATCGATAGAGAAGCGTTGGGCGTCGCGAGATAAAAAAGTGGAGAACATGTGGGATCTTCTGATGAGGAAGTCTTCATTATTGGCATAGATGGAATGGGGGGAGTTGGAAAAACATTCATGGCAACTCATATCAAGAATGAGATTAAAAGAAAGGGGACTTTCAAGGATGTCTTCTGGGTCACTGTTTCCCATGATTTCACCAATTTCAAA

>AG3931_Paralog_D

GGGTCGACTAATGAATTCCCCAAGCCTTCTGAATTGGATGATGAGTATGATGATTTATTGGAAGAGAATCCTTGGGTGTCGCGAGATGAAAATGTGAAGGAGATGTGGGATCTTCTGGAGGATGAGGAAGTCTTCATTATTGGCATAGATGGAATGGGGGGAGTTGGAAAAACATTCATGGCAACTCATATCAAGAATGAGATTAAAAGAAAGGGGACTTTCAAGGATGTCTTCTGGGTCACTGTTTCCGATGATTTCACCACTTTCAAA

>D_Del17bp_D

GGGTCGACTAATGAATTCCCCAAGCCTTCTGAATTGGATGATGAGTATGATGATTTATTGGAAGAGAATCCTTGGGTGTCGCGAGATGAAAATGTGAAGGAGATATGAGGAAGTCTTCATTATTGGCATAGATGGAATGGGGGGAGTTGGAAAAACATTCATGGCAACTCATATCAAGAATGAGATTAAAAGAAAGGGGACTTTCAAGGATGTCTTCTGGGTCACTGTTTCCGATGATTTCACCACTTTCAAA

R1-34

>AG3931_Paralog_A

GGGTCGACTAATGAATTGCCCAAGCCTTCTGAATTGCATGCTAAGTTTATTTATTTATTGATAGAGAAGCTTTGGGAGTTGCGAGATGAAAATGTGAAGAAGATGTGGGATCTTCTGGAGGATGAGGAAGTCTTCATTATTGGCATAGATGGAATGGGGGGAGTTGGAAAAACATTCATGGCAACTCATTTCAAGAATGAGATTAAAAGAAAGGGGACTTTCAAGGATGTCTTCTGGGTCACTGTTTCCCATGATTTCACCATTTTCAAATTGCAACATCACATTGCAGAAACAATGCAGGTTAAGCTTT

>A_Del1bp_A

GGGTCGACTAATGAATTGCCCAAGCCTTCTGAATTGCATGCTAAGTTTATTTATTTATTGATAGAGAAGCTTTGGGAGTTGCGAGATGAAAATGTGAAGAAGATGTGGGATCTTCTGGAGATGAGGAAGTCTTCATTATTGGCATAGATGGAATGGGGGGAGTTGGAAAAACATTCATGGCAACTCATTTCAAGAATGAGATTAAAAGAAAGGGGACTTTCAAGGATGTCTTCTGGGTCACTGTTTCCCATGATTTCACCATTTTCAAATTGCAACATCACATTGCAGAAACAATGCAGGTTAAGCTTT

>AG3931_Paralog_B

GGGTCGACTAATGAATTGGATGAGCCTTCTGAATTGGATATTGAGTTTGATGTATTATTGATACAGAAGCCTTGGGGGTTGCGAGATAAAAATGTGAAGAAGATGTGGGATCTTCTGGAGGATGAGGAAGTCTTCATTATTGGCATAGATGGAATGGGGGGAGTTGGAAAAACATTCATGGCAACTCATATCAAGAATGAGATTAAAAGAAAGGGGACTTTCAAGGATGTCTTCTGGGTCACTGTTTCCGATGATTTCACCACTTTCAAATTGCAACATGACATTGCAGAAACAATACAGGTTAAGCTTT

>B_Ins1bp_B

GGGTCGACTAATGAATTGGATGAGCCTTCTGAATTGGATATTGAGTTTGATGTATTATTGATACAGAAGCCTTGGGGGTTGCGAGATAAAAATGTGAAGAAGATGTGGGATCTTCTGGAGGTATGAGGAAGTCTTCATTATTGGCATAGATGGAATGGGGGGAGTTGGAAAAACATTCATGGCAACTCATATCAAGAATGAGATTAAAAGAAAGGGGACTTTCAAGGATGTCTTCTGGGTCACTGTTTCCGATGATTTCACCACTTTCAAATTGCAACATGACATTGCAGAAACAATACAGGTTAAGCTTT

>B_Ins26bp_B

GGGTCGACTAATGAATTGGATGAGCCTTCTGAATTGGATATTGAGTTTGATGTATTATTGATACAGAAGCCTTGGGGGTTGCGAGATAAAAATGTGAAGAAGATGTGGGATCTCATATCAAGAGGAAGTCTTCATTATTATGAGGAAGTCTTCATTATTGGCATAGATGGAATGGGGGGAGTTGGAAAAACATTCATGGCAACTCATATCAAGAATGAGATTAAAAGAAAGGGGACTTTCAAGGATGTCTTCTGGGTCACTGTTTCCGATGATTTCACCACTTTCAAATTGCAACATGACATTGCAGAAACAATACAGGTTAAGCTTT

>AG3931_Paralog_D

GGGTCGACTAATGAATTCCCCAAGCCTTCTGAATTGGATGATGAGTATGATGATTTATTGGAAGAGAATCCTTGGGTGTCGCGAGATGAAAATGTGAAGGAGATGTGGGATCTTCTGGAGGATGAGGAAGTCTTCATTATTGGCATAGATGGAATGGGGGGAGTTGGAAAAACATTCATGGCAACTCATATCAAGAATGAGATTAAAAGAAAGGGGACTTTCAAGGATGTCTTCTGGGTCACTGTTTCCGATGATTTCACCACTTTCAAATTGCAACATGACATTGCAGAAACAATACAGGTTAAGCTTT

>D_Del1bp_D

GGGTCGACTAATGAATTCCCCAAGCCTTCTGAATTGGATGATGAGTATGATGATTTATTGGAAGAGAATCCTTGGGTGTCGCGAGATGAAAATGTGAAGGAGATGTGGGATCTTCTGGAGATGAGGAAGTCTTCATTATTGGCATAGATGGAATGGGGGGAGTTGGAAAAACATTCATGGCAACTCATATCAAGAATGAGATTAAAAGAAAGGGGACTTTCAAGGATGTCTTCTGGGTCACTGTTTCCGATGATTTCACCACTTTCAAATTGCAACATGACATTGCAGAAACAATACAGGTTAAGCTTT

>D_Del6bp_D

GGGTCGACTAATGAATTCCCCAAGCCTTCTGAATTGGATGATGAGTATGATGATTTATTGGAAGAGAATCCTTGGGTGTCGCGAGATGAAAATGTGAAGGAGATGTGGGATCTTCTGGAGGAAGTCTTCATTATTGGCATAGATGGAATGGGGGGAGTTGGAAAAACATTCATGGCAACTCATATCAAGAATGAGATTAAAAGAAAGGGGACTTTCAAGGATGTCTTCTGGGTCACTGTTTCCGATGATTTCACCACTTTCAAATTGCAACATGACATTGCAGAAACAATACAGGTTAAGCTTT

R1-35

>AG3931_Paralog_A

GGGTCGACTAATGAATTGCCCAAGCCTTCTGAATTGCATGCTAAGTTTATTTATTTATTGATAGAGAAGCTTTGGGAGTTGCGAGATGAAAATGTGAAGAAGATGTGGGATCTTCTGGAGGATGAGGAAGTCTTCATTATTGGCATAGATGGAATGGGGGGAGTTGGAAAAACATTCATGGCAACTCATTTCAAGAATGAGATTAAAAGAAAGGGGACTTTCAAGGATGTCTTCTGGGTCACTGTTTCCCATGATTTCACCATTTTCAAA

>A_Ins1bp_A

GGGTCGACTAATGAATTGCCCAAGCCTTCTGAATTGCATGCTAAGTTTATTTATTTATTGATAGAGAAGCTTTGGGAGTTGCGAGATGAAAATGTGAAGAAGATGTGGGATCTTCTGGAGGTATGAGGAAGTCTTCATTATTGGCATAGATGGAATGGGGGGAGTTGGAAAAACATTCATGGCAACTCATTTCAAGAATGAGATTAAAAGAAAGGGGACTTTCAAGGATGTCTTCTGGGTCACTGTTTCCCATGATTTCACCATTTTCAAA

>AG3931_Paralog_B

GGGTCGACTAATGAATTGGATGAGCCTTCTGAATTGGATATTGAGTTTGATGTATTATTGATACAGAAGCCTTGGGGGTTGCGAGATAAAAATGTGAAGAAGATGTGGGATCTTCTGGAGGATGAGGAAGTCTTCATTATTGGCATAGATGGAATGGGGGGAGTTGGAAAAACATTCATGGCAACTCATATCAAGAATGAGATTAAAAGAAAGGGGACTTTCAAGGATGTCTTCTGGGTCACTGTTTCCGATGATTTCACCACTTTCAAA

>B_Del1bp_C

GGGTCGACTAATGAATTGGATGAGCCTTCTGAATTGGATATTGAGTTTGATGTATTATTGATACAGAAGCCTTGGGGGTTGCGAGATAAAAATGTGAAGAAGATGTGGGATCTTCTGGAGATGAGGAAGTCTTCATTATTGGCATAGATGGAATGGGGGGAGTTGGAAAAACATTCATGGCAACTCATATCAAGAATGAGATTAAAAGAAAGGGGACTTTCAAGGATGTCTTCTGGGTCACTGTTTCCCATGATTTCACCAATTTCAAA

>B_Del6bp_C

GGGTCGACTAATGAATTGGATGAGCCTTCTGAATTGGATATTGAGTTTGATGTATTATTGATACAGAAGCCTTGGGGGTTGCGAGATAAAAATGTGAAGAAGATGTGGGATCTTCATGAGGAAGTCTTCATTATTGGCATAGATGGAATGGGGGGAGTTGGAAAAACATTCATGGCAACTCATATCAAGAATGAGATTAAAAGAAAGGGGACTTTCAAGGATGTCTTCTGGGTCACTGTTTCCCATGATTTCACCAATTTCAAA

>B_Del19bp_C

GGGTCGACTAATGAATTGGATGAGCCTTCTGAATTGGATATTGAGTTTGATGTATTATTGATACAGAAGCCTTGGGGGTTGCGAGATAAAAATGTGAAGAAGATGAGGAAGTCTTCATTATTGGCATAGATGGAATGGGGGGAGTTGGAAAAACATTCATGGCAACTCATATCAAGAATGAGATTAAAAGAAAGGGGACTTTCAAGGATGTCTTCTGGGTCACTGTTTCCCATGATTTCACCAATTTCAAA

>AG3931_Paralog_C

GGGTCGACTAATGAATTCCCCAAGCCTTCTGAATTGGGTGATGAGTTTTTTTATTTATCGATAGAGAAGCGTTGGGCGTCGCGAGATAAAAAAGTGGAGAACATGTGGGATCTTCTGGAGGATGAGGAAGTCTTCATTATTGGCATAGATGGAATGGGGGGAGTTGGAAAAACATTCATGGCAACTCATATCAAGAATGAGATTAAAAGAAAGGGGACTTTCAAGGATGTCTTCTGGGTCACTGTTTCCCATGATTTCACCAATTTCAAA

R1-36

>AG3931_Paralog_A

GGGTCGACTAATGAATTGCCCAAGCCTTCTGAATTGCATGCTAAGTTTATTTATTTATTGATAGAGAAGCTTTGGGAGTTGCGAGATGAAAATGTGAAGAAGATGTGGGATCTTCTGGAGGATGAGGAAGTCTTCATTATTGGCATAGATGGAATGGGGGGAGTTGGAAAAACATTCATGGCAACTCATTTCAAGAATGAGATTAAAAGAAAGGGGACTTTCAAGGATGTCTTCTGGGTCACTGTTTCCCATGATTTCACCATTTTCAAA

>A_Del1bp_A

GGGTCGACTAATGAATTGCCCAAGCCTTCTGAATTGCATGCTAAGTTTATTTATTTATTGATAGAGAAGCTTTGGGAGTTGCGAGATGAAAATGTGAAGAAGATGTGGGATCTTCTGGAGATGAGGAAGTCTTCATTATTGGCATAGATGGAATGGGGGGAGTTGGAAAAACATTCATGGCAACTCATTTCAAGAATGAGATTAAAAGAAAGGGGACTTTCAAGGATGTCTTCTGGGTCACTGTTTCCCATGATTTCACCATTTTCAAA

R1-37

>AG3931_Paralog_A

GGGTCGACTAATGAATTGCCCAAGCCTTCTGAATTGCATGCTAAGTTTATTTATTTATTGATAGAGAAGCTTTGGGAGTTGCGAGATGAAAATGTGAAGAAGATGTGGGATCTTCTGGAGGATGAGGAAGTCTTCATTATTGGCATAGATGGAATGGGGGGAGTTGGAAAAACATTCATGGCAACTCATTTCAAGAATGAGATTAAAAGAAAGGGGACTTTCAAGGATGTCTTCTGGGTCACTGTTTCCCATGATTTCACCATTTTCAAA

>A_Del3bp_A

GGGTCGACTAATGAATTGCCCAAGCCTTCTGAATTGCATGCTAAGTTTATTTATTTATTGATAGAGAAGCTTTGGGAGTTGCGAGATGAAAATGTGAAGAAGATGTGGGATCTTCTGGATGAGGAAGTCTTCATTATTGGCATAGATGGAATGGGGGGAGTTGGAAAAACATTCATGGCAACTCATTTCAAGAATGAGATTAAAAGAAAGGGGACTTTCAAGGATGTCTTCTGGGTCACTGTTTCCCATGATTTCACCATTTTCAAA

R1-38

>AG3931_Paralog_A

GGGTCGACTAATGAATTGCCCAAGCCTTCTGAATTGCATGCTAAGTTTATTTATTTATTGATAGAGAAGCTTTGGGAGTTGCGAGATGAAAATGTGAAGAAGATGTGGGATCTTCTGGAGGATGAGGAAGTCTTCATTATTGGCATAGATGGAATGGGGGGAGTTGGAAAAACATTCATGGCAACTCATTTCAAGAATGAGATTAAAAGAAAGGGGACTTTCAAGGATGTCTTCTGGGTCACTGTTTCCCATGATTTCACCATTTTCAAA

>A_Del3bp_A

GGGTCGACTAATGAATTGCCCAAGCCTTCTGAATTGCATGCTAAGTTTATTTATTTATTGATAGAGAAGCTTTGGGAGTTGCGAGATGAAAATGTGAAGAAGATGTGGGATCTTCTGGATGAGGAAGTCTTCATTATTGGCATAGATGGAATGGGGGGAGTTGGAAAAACATTCATGGCAACTCATTTCAAGAATGAGATTAAAAGAAAGGGGACTTTCAAGGATGTCTTCTGGGTCACTGTTTCCCATGATTTCACCATTTTCAAA

R1-39

>AG3931_Paralog_A

GGGTCGACTAATGAATTGCCCAAGCCTTCTGAATTGCATGCTAAGTTTATTTATTTATTGATAGAGAAGCTTTGGGAGTTGCGAGATGAAAATGTGAAGAAGATGTGGGATCTTCTGGAGGATGAGGAAGTCTTCATTATTGGCATAGATGGAATGGGGGGAGTTGGAAAAACATTCATGGCAACTCATTTCAAGAATGAGATTAAAAGAAAGGGGACTTTCAAGGATGTCTTCTGGGTCACTGTTTCCCATGATTTCACCATTTTCAAA

>A_Del3bp_A

GGGTCGACTAATGAATTGCCCAAGCCTTCTGAATTGCATGCTAAGTTTATTTATTTATTGATAGAGAAGCTTTGGGAGTTGCGAGATGAAAATGTGAAGAAGATGTGGGATCTTCTGGATGAGGAAGTCTTCATTATTGGCATAGATGGAATGGGGGGAGTTGGAAAAACATTCATGGCAACTCATTTCAAGAATGAGATTAAAAGAAAGGGGACTTTCAAGGATGTCTTCTGGGTCACTGTTTCCCATGATTTCACCATTTTCAAA

**AG3931 R1 mutants at TS2**

R1-40

>AG3931_Paralog_A

TTTGTTGTTAGGTTGTTTGATGTTACGAATGAATGGTCTGGTGAGGAAGATGGCGTGCCATATCTTGAACGACAATCACACTTACTTGATAAAATGTAATGAAAAATTGAGAAAGATGCCTCAGATGCGGGAATGGACAGCTGATCTGGAGGCAGTTTCTTTGGCGGGTAATGAGATAGAAGAAATAGCAGAGGGCACATCACCTAATTGTCCACGCTTGTCCACCTTCATCTTATCTCGTAATTCCATCAGTCATATTCCCAAGTGTTTTTTCAGACGCATGAACGCTCTAACACAACTTGATTT

>A_Del6bp_A

TTTGTTGTTAGGTTGTTTGATGTTACGAATGAATGGTCTGGTGAGGAAGATGGCGTGCCATATCTTGAACGACAATCACACTTACTTGATAAAATGTAATGAAAAATTGAGAAAGATGCCTCAGATGCGGGAATGGACAGCGGAGGCAGTTTCTTTGGCGGGTAATGAGATAGAAGAAATAGCAGAGGGCACATCACCTAATTGTCCACGCTTGTCCACCTTCATCTTATCTCGTAATTCCATCAGTCATATTCCCAAGTGTTTTTTCAGACGCATGAACGCTCTAACACAACTTGATTT

>AG3931_Paralog_B

TTTGTTGTTAGATCGTGGGAGTTTACGAATGCATGGTCTGGTGAGGAAGATGGCGTGCCATATCTTGAACGAGAATCACACTTACATGATAAAATGTGATGAAAATTTGAGAAAGATACCTCAGATGCGGGAATGGACAGCTGATCTGGAGGCAGTTTCTTTGGCGGGTAATGAGATAGAAGAAATAGCAGAGGGCACATCACCTAATTGTCCAGGCTTGTCCACCTTGATTTTATCTCATAATTTGATCAGTCATATTCCCAAGTGTTTTTTCAGACACATGAATGCTCTAACACTACTTGATTT

>B_Intact_B

TTTGTTGTTAGATCGTGGGAGTTTACGAATGCATGGTCTGGTGAGGAAGATGGCGTGCCATATCTTGAACGAGAATCACACTTACATGATAAAATGTGATGAAAATTTGAGAAAGATACCTCAGATGCGGGAATGGACAGCTGATCTGGAGGCAGTTTCTTTGGCGGGTAATGAGATAGAAGAAATAGCAGAGGGCACATCACCTAATTGTCCAGGCTTGTCCACCTTGATTTTATCTCATAATTTGATCAGTCATATTCCCAAGTGTTTTTTCAGACACATGAATGCTCTAACACTACTTGATTT

>AG3931_Paralog_D

TTTGTTGTTAGGTGGTTGGAGGTTACGAATGAATGGTCTGGTGAGGAAGATGGCGTGCAATATCTTGAACGAGAATCACACTTACATGATAAAATGTCATGAAAATTTGACAAAGATACCTCAGATGCGGGAATGGACAGCTGATCTGGAGGCCGTTTCTTTGGCGGGTAATGAGATAGAAGAAATAGCAGAGGGCACATCACCTAATTGTCCTCGCTTGTCCACCTTCATCTTATCTCGTAATTCCATCAGTCATATTCCCAAGTGTTTTTTCAGACACATGAATGCTCTAACACTACTTGATTT

>D_Intact_D

TTTGTTGTTAGGTGGTTGGAGGTTACGAATGAATGGTCTGGTGAGGAAGATGGCGTGCAATATCTTGAACGAGAATCACACTTACATGATAAAATGTCATGAAAATTTGACAAAGATACCTCAGATGCGGGAATGGACAGCTGATCTGGAGGCCGTTTCTTTGGCGGGTAATGAGATAGAAGAAATAGCAGAGGGCACATCACCTAATTGTCCTCGCTTGTCCACCTTCATCTTATCTCGTAATTCCATCAGTCATATTCCCAAGTGTTTTTTCAGACACATGAATGCTCTAACACTACTTGATTT

R1-41

>AG3931_Paralog_A

TTTGTTGTTAGGTTGTTTGATGTTACGAATGAATGGTCTGGTGAGGAAGATGGCGTGCCATATCTTGAACGACAATCACACTTACTTGATAAAATGTAATGAAAAATTGAGAAAGATGCCTCAGATGCGGGAATGGACAGCTGATCTGGAGGCAGTTTCTTTGGCGGGTAATGAGATAGAAGAAATAGCAGAGGGCACATCACCTAATTGTCCACGCTTGTCCACCTTCATCTTATCTCGTAATTCCATCAGTCATATTCCCAAGTGTTTTTTCAGACGCATGAACGCTCTAACACAACTTGATTTA

>A_Del6bp_A

TTTGTTGTTAGGTTGTTTGATGTTACGAATGAATGGTCTGGTGAGGAAGATGGCGTGCCATATCTTGAACGACAATCACACTTACTTGATAAAATGTAATGAAAAATTGAGAAAGATGCCTCAGATGCGGGAATGGACAGCGGAGGCAGTTTCTTTGGCGGGTAATGAGATAGAAGAAATAGCAGAGGGCACATCACCTAATTGTCCACGCTTGTCCACCTTCATCTTATCTCGTAATTCCATCAGTCATATTCCCAAGTGTTTTTTCAGACGCATGAACGCTCTAACACAACTTGATTTA

>AG3931_Paralog_B

TTTGTTGTTAGATCGTGGGAGTTTACGAATGCATGGTCTGGTGAGGAAGATGGCGTGCCATATCTTGAACGAGAATCACACTTACATGATAAAATGTGATGAAAATTTGAGAAAGATACCTCAGATGCGGGAATGGACAGCTGATCTGGAGGCAGTTTCTTTGGCGGGTAATGAGATAGAAGAAATAGCAGAGGGCACATCACCTAATTGTCCAGGCTTGTCCACCTTGATTTTATCTCATAATTTGATCAGTCATATTCCCAAGTGTTTTTTCAGACACATGAATGCTCTAACACTACTTGATTTA

>B_Del2bp_B

TTTGTTGTTAGATCGTGGGAGTTTACGAATGCATGGTCTGGTGAGGAAGATGGCGTGCCATATCTTGAACGAGAATCACACTTACATGATAAAATGTGATGAAAATTTGAGAAAGATACCTCAGATGCGGGAATGGACAGCTGATGGAGGCAGTTTCTTTGGCGGGTAATGAGATAGAAGAAATAGCAGAGGGCACATCACCTAATTGTCCAGGCTTGTCCACCTTGATTTTATCTCATAATTTGATCAGTCATATTCCCAAGTGTTTTTTCAGACACATGAATGCTCTAACACTACTTGATTTA

>AG3931_Paralog_D

TTTGTTGTTAGGTGGTTGGAGGTTACGAATGAATGGTCTGGTGAGGAAGATGGCGTGCAATATCTTGAACGAGAATCACACTTACATGATAAAATGTCATGAAAATTTGACAAAGATACCTCAGATGCGGGAATGGACAGCTGATCTGGAGGCCGTTTCTTTGGCGGGTAATGAGATAGAAGAAATAGCAGAGGGCACATCACCTAATTGTCCTCGCTTGTCCACCTTCATCTTATCTCGTAATTCCATCAGTCATATTCCCAAGTGTTTTTTCAGACACATGAATGCTCTAACACTACTTGATTTA

>D_Intact_D

TTTGTTGTTAGGTGGTTGGAGGTTACGAATGAATGGTCTGGTGAGGAAGATGGCGTGCAATATCTTGAACGAGAATCACACTTACATGATAAAATGTCATGAAAATTTGACAAAGATACCTCAGATGCGGGAATGGACAGCTGATCTGGAGGCCGTTTCTTTGGCGGGTAATGAGATAGAAGAAATAGCAGAGGGCACATCACCTAATTGTCCTCGCTTGTCCACCTTCATCTTATCTCGTAATTCCATCAGTCATATTCCCAAGTGTTTTTTCAGACACATGAATGCTCTAACACTACTTGATTTA

R1-42

>AG3931_Paralog_B

TTTGTTGTTAGATCGTGGGAGTTTACGAATGCATGGTCTGGTGAGGAAGATGGCGTGCCATATCTTGAACGAGAATCACACTTACATGATAAAATGTGATGAAAATTTGAGAAAGATACCTCAGATGCGGGAATGGACAGCTGATCTGGAGGCAGTTTCTTTGGCGGGTAATGAGATAGAAGAAATAGCAGAGGGCACATCACCTAATTGTCCAGGCTTGTCCACCTTGATTTTATCTCATAATTTGATCAGTCATATTCCCAAGTGTTTTTTCAGACACATGAATGCTCTAACACTACTTGATTT

>B_Del7bp_B

TTTGTTGTTAGATCGTGGGAGTTTACGAATGCATGGTCTGGTGAGGAAGATGGCGTGCCATATCTTGAACGAGAATCACACTTACATGATAAAATGTGATGAAAATTTGAGAAAGATACCTCAGATGCGGGAATGGACATGGAGGCAGTTTCTTTGGCGGGTAATGAGATAGAAGAAATAGCAGAGGGCACATCACCTAATTGTCCAGGCTTGTCCACCTTGATTTTATCTCATAATTTGATCAGTCATATTCCCAAGTGTTTTTTCAGACACATGAATGCTCTAACACTACTTGATTT

>AG3931_Paralog_C

TTTGTTGTTAGGTTATTGGAGTTTACGAATGAATGGTCTGTTGAGGAAGATGGCGTGCAATATCTTGAACGAGAATCACACTTACATGATAAAATGTCATGAAAATTTGAGAAAGATACCTCAGATGCGGGAATGGACAGCTGATCTGGAGGCAGTTTCTTTGGCGGGTAATGAGATAGAAGAAATAGCAGAGGGCACATCACCTAATTGTCCACGCTTGTCCACCTTCATCTTATCTCGTAATTCCATCAGTCATATTCCCAAGTGTTTTTTCAGACACATGAACGCTCTAACACAACTTGATTT

>C_Del1bp_C

TTTGTTGTTAGGTTATTGGAGTTTACGAATGAATGGTCTGTTGAGGAAGATGGCGTGCAATATCTTGAACGAGAATCACACTTACATGATAAAATGTCATGAAAATTTGAGAAAGATACCTCAGATGCGGGAATGGACAGCTGATTGGAGGCAGTTTCTTTGGCGGGTAATGAGATAGAAGAAATAGCAGAGGGCACATCACCTAATTGTCCACGCTTGTCCACCTTCATCTTATCTCGTAATTCCATCAGTCATATTCCCAAGTGTTTTTTCAGACACATGAACGCTCTAACACAACTTGATTT

>C_Del2bp_C

TTTGTTGTTAGGTTATTGGAGTTTACGAATGAATGGTCTGTTGAGGAAGATGGCGTGCAATATCTTGAACGAGAATCACACTTACATGATAAAATGTCATGAAAATTTGAGAAAGATACCTCAGATGCGGGAATGGACAGCTGATGGAGGCAGTTTCTTTGGCGGGTAATGAGATAGAAGAAATAGCAGAGGGCACATCACCTAATTGTCCACGCTTGTCCACCTTCATCTTATCTCGTAATTCCATCAGTCATATTCCCAAGTGTTTTTTCAGACACATGAACGCTCTAACACAACTTGATTT

**AG3931 R1 mutants at TS3**

R1-43

>AG3931_Paralog_A

TTTATCATTTAATCTTAGATTAACATCTTTGCCAAAGTCGCTGTCTAAGTTGAGGTCTCTTACTTCTTTAGTGCTCCGTCAATGTTCAAAATTGAAAGATATACCTCCACTGGGAGATCTACAAGCATTGTCAAGATTGGACATTTCAGGTTGTGATTCGCTCCTCAGGGTACCGGAAGGCTTGCAAAATCTAAAAAAGTTGCAATGCCTTAATCTTTCCCGCGATTTGTATTT

>A_Ins1bp_A

TTTATCATTTAATCTTAGATTAACATCTTTGCCAAAGTCGCTGTCTAAGTTGAGGTCTCTTACTTCTTTAGTGCTCCGTCAATGTTCAAAATTGAAAGATATACCTCCAACTGGGAGATCTACAAGCATTGTCAAGATTGGACATTTCAGGTTGTGATTCGCTCCTCAGGGTACCGGAAGGCTTGCAAAATCTAAAAAAGTTGCAATGCCTTAATCTTTCCCGCGATTTGTATTT

>AG3931_Paralog_C

TTTATCATATAATCGTAGATTAACATCTTTGCCAAAGTCGCTGTCTAAGTTGAGGTCTCTTACTTCTTTAGTGCTCCGCCAATGTTCAAAATTGAAAGATATACCTCCACTGGGAGATCTACAAGCATTGTCAAGATTGGACATTTCCGGTTGTAATTCGCTCCTCAGGGTACCGGAAGGCTTGCAAAATCTAAAAAAGTTGCAATGGCTTAGTCTTTCCCGCAAGCTGAATTT

>C_Del1bp_C

TTTATCATATAATCGTAGATTAACATCTTTGCCAAAGTCGCTGTCTAAGTTGAGGTCTCTTACTTCTTTAGTGCTCCGCCAATGTTCAAAATTGAAAGATATACCTCCCTGGGAGATCTACAAGCATTGTCAAGATTGGACATTTCCGGTTGTAATTCGCTCCTCAGGGTACCGGAAGGCTTGCAAAATCTAAAAAAGTTGCAATGGCTTAGTCTTTCCCGCAAGCTGAATTT

R1-44

>AG3931_Paralog_A

TTTATCATTTAATCTTAGATTAACATCTTTGCCAAAGTCGCTGTCTAAGTTGAGGTCTCTTACTTCTTTAGTGCTCCGTCAATGTTCAAAATTGAAAGATATACCTCCACTGGGAGATCTACAAGCATTGTCAAGATTGGACATTTCAGGTTGTGATTCGCTCCTCAGGGTACCGGAAGGCTTGCAAAATCTAAAAAAGTTGCAATGCCTTAATCTTTCCCGCGATTTGTATTTATCATTGTTACCCGGATGCGCACTGCCCGGTTTG

>A_Ins1bp_A

TTTATCATTTAATCTTAGATTAACATCTTTGCCAAAGTCGCTGTCTAAGTTGAGGTCTCTTACTTCTTTAGTGCTCCGTCAATGTTCAAAATTGAAAGATATACCTCCAACTGGGAGATCTACAAGCATTGTCAAGATTGGACATTTCAGGTTGTGATTCGCTCCTCAGGGTACCGGAAGGCTTGCAAAATCTAAAAAAGTTGCAATGCCTTAATCTTTCCCGCGATTTGTATTTATCATTGTTACCCGGATGCGCACTGCCCGGTTTG

>AG3931_Paralog_C

TTTATCATATAATCGTAGATTAACATCTTTGCCAAAGTCGCTGTCTAAGTTGAGGTCTCTTACTTCTTTAGTGCTCCGCCAATGTTCAAAATTGAAAGATATACCTCCACTGGGAGATCTACAAGCATTGTCAAGATTGGACATTTCCGGTTGTAATTCGCTCCTCAGGGTACCGGAAGGCTTGCAAAATCTAAAAAAGTTGCAATGGCTTAGTCTTTCCCGCAAGCTGAATTTATCATTAGTACCGTTATGCGTACTGCCCGGTTTG

>C_Del7bp_C

TTTATCATATAATCGTAGATTAACATCTTTGCCAAAGTCGCTGTCTAAGTTGAGGTCTCTTACTTCTTTAGTGCTCCGCCAATGTTCAAAATTGAAAGATATACCTCAGATCTACAAGCATTGTCAAGATTGGACATTTCCGGTTGTAATTCGCTCCTCAGGGTACCGGAAGGCTTGCAAAATCTAAAAAAGTTGCAATGGCTTAGTCTTTCCCGCAAGCTGAATTTATCATTAGTACCGTTATGCGTACTGCCCGGTTTG

R1-45

>AG3931_Paralog_B

TTTATCATATAATTATGAGTTAACATCTTTGCCAAAGTCTCTGTCTAAGTTGAGGTCTCTTACTTCTTTAGTGCTCCGTCAATGTTCAAAATTGAAAGATATACCTCCACTGGGAGATCTACAAGCATTGTCAAGATTGGACATTTCAGGTTGTGATTCGCTCCTCAGGGTACCGGAAGGCTTGCAAAATCTAAAAAAGTTGCAATGCCTTAATCTTTCCCGCGATTTGTATTT

>B_Intact_B

TTTATCATATAATTATGAGTTAACATCTTTGCCAAAGTCTCTGTCTAAGTTGAGGTCTCTTACTTCTTTAGTGCTCCGTCAATGTTCAAAATTGAAAGATATACCTCCACTGGGAGATCTACAAGCATTGTCAAGATTGGACATTTCAGGTTGTGATTCGCTCCTCAGGGTACCGGAAGGCTTGCAAAATCTAAAAAAGTTGCAATGCCTTAATCTTTCCCGCGATTTGTATTT

>B_Del2bp_B

TTTATCATATAATTATGAGTTAACATCTTTGCCAAAGTCTCTGTCTAAGTTGAGGTCTCTTACTTCTTTAGTGCTCCGTCAATGTTCAAAATTGAAAGATATACCTCCTGGGAGATCTACAAGCATTGTCAAGATTGGACATTTCAGGTTGTGATTCGCTCCTCAGGGTACCGGAAGGCTTGCAAAATCTAAAAAAGTTGCAATGCCTTAATCTTTCCCGCGATTTGTATTT

>B_Del4bp_B

TTTATCATATAATTATGAGTTAACATCTTTGCCAAAGTCTCTGTCTAAGTTGAGGTCTCTTACTTCTTTAGTGCTCCGTCAATGTTCAAAATTGAAAGATATACCTCCAGAGATCTACAAGCATTGTCAAGATTGGACATTTCAGGTTGTGATTCGCTCCTCAGGGTACCGGAAGGCTTGCAAAATCTAAAAAAGTTGCAATGCCTTAATCTTTCCCGCGATTTGTATTT

R1-46

>AG3931_Paralog_B

TTTATCATATAATTATGAGTTAACATCTTTGCCAAAGTCTCTGTCTAAGTTGAGGTCTCTTACTTCTTTAGTGCTCCGTCAATGTTCAAAATTGAAAGATATACCTCCACTGGGAGATCTACAAGCATTGTCAAGATTGGACATTTCAGGTTGTGATTCGCTCCTCAGGGTACCGGAAGGCTTGCAAAATCTAAAAAAGTTGCAATGCCTTAATCTTTCCCGCGATTTGTATTT

>B_Intact_B

TTTATCATATAATTATGAGTTAACATCTTTGTCAAAGTCTCTGTCTAAGTTGAGGTCTCTTACTTCTTTAGTGCTCCGTCAATGTTCAAAATTGAAAGATATACCTCCACTGGGAGATCTACAAGCATTGTCAAGATTGGACATTTCAGGTTGTGATTCGCTCCTCAGGGTACCGGAAGGCTTGCAAAATCTAAAAAAGTTGCAATGCCTTAATCTTTCCCGCGATTTGTATTT

>B_Ins1bp_B

TTTATCATATAATTATGAGTTAACATCTTTGCCAAAGTCTCTGTCTAAGTTGAGGTCTCTTACTTCTTTAGTGCTCCGTCAATGTTCAAAATTGAAAGATATACCTCCAACTGGGAGATCTACAAGCATTGTCAAGATTGGACATTTCAGGTTGTGATTCGCTCCTCAGGGTACCGGAAGGCTTGCAAAATCTAAAAAAGTTGCAATGCCTTAATCTTTCCCGCGATTTGTATTT

>B_Del1bp_B

TTTATCATATAATTATGAGTTAACATCTTTGCCAAAGTCTCTGTCTAAGTTGAGGTCTCTTACTTCTTTAGTGCTCCGTCAATGTTCAAAATTGAAAGATATACCTCCATGGGAGATCTACAAGCATTGTCAAGATTGGACATTTCAGGTTGTGATTCGCTCCTCAGGGTACCGGAAGGCTTGCAAAATCTAAAAAAGTTGCAATGCCTTAATCTTTCCCGCGATTTGTATTT

>AG3931_Paralog_D

TTTATCATATAATTATGAGTTAACATCTTTGCCAAAGTCTCTGTCTAAGTTGAGGTCTCTTACTTCTTTAGTGCTCCGTGAATGTCGTCAATTGGAATATATACCTCCACTGGGAGATCTACATGCATTGTCAAGATTGGACATTTCAGGTTGTGATTCGCTCCTCAGGGTACCGGAAGGCTTGCAAAATCTAAAAAAGTTGCAATGCCTTAATCTTTCCCGCGATTTGTATTT

>D_Del8bp_D

TTTATCATATAATTATGAGTTAACATCTTTGCCAAAGTCTCTGTCTAAGTTGAGGTCTCTTACTTCTTTAGTGCTCCGTGAATGTCGTCAATTGGAATATATACCTAGATCTACATGCATTGTCAAGATTGGACATTTCAGGTTGTGATTCGCTCCTCAGGGTACCGGAAGGCTTGCAAAATCTAAAAAAGTTGCAATGCCTTAATCTTTCCCGCGATTTGTATTT

R1-47

>AG3931_Paralog_B

TTTATCATATAATTATGAGTTAACATCTTTGCCAAAGTCTCTGTCTAAGTTGAGGTCTCTTACTTCTTTAGTGCTCCGTCAATGTTCAAAATTGAAAGATATACCTCCACTGGGAGATCTACAAGCATTGTCAAGATTGGACATTTCAGGTTGTGATTCGCTCCTCAGGGTACCGGAAGGCTTGCAAAATCTAAAAAAGTTGCAATGCCTTAATCTTTCCCGCGATTTGTATTT

>B_Intact_B

TTTATCATATAATTATGAGTTAACATCTTTGCCAAAGTCTCTGTCTAAGTTGAGGTCTCTTACTTCTTTAGTGCTCCGTCAATGTTCAAAATTGAAAGATATACCTCCATCTGGGAGATCTACAAGCATTGTCAAGATGGGACATTTCAGGTTGTGATTCGCTCCTCAGGGTACCGGAAGGCTTGCAAAATCTAAAAAAGTTGCAATGCCTTAATCTTTCCCGCGATTTGTATTT

>B_Del8bp_B

TTTATCATATAATTATGAGTTAACATCTTTGCCAAAGTCTCTGTCTAAGTTGAGGTCTCTTACTTCTTTAGTGCTCCGTCAATGTTCAAAATTGAAAGATATACCTCCATCTACAAGCATTGTCAAGATTGGACATTTCAGGTTGTGATTCGCTCCTCAGGGTACCGGAAGGCTTGCAAAATCTAAAAAAGTTGCAATGCCTTAATCTTTCCCGCGATTTGTATTT

**Supplementary Data S3**

**Inverse PCR (iPCR) amplicon sequences**

>Virtual iPCR amplicon spanning the AseI-NdeI junction (shaded) of paralog A.

TCTTCTACATCACCTGTTTCATTGTTTTGGTTATGCTACATTCTTAAATTTTTTCTGTTGCTCCTTCACTGTAGAATAACACAACTCTCTCGTGTGTTTTAATAATGAGTCTTTAGACAGAAAATTCTATTTTTAAATTGATTTACAATATATTATATGTCAAAATAATTTATTTAGAAATATATTAAATTAAAAGAATTTCAAACTCACACATAACAAAAAGAATTATTTTACCCAAATAAAGAATATATTTAAAAACTGAAAGGAATTATGAAATTGCAATGCCCGGAAATACATTCTTCCCCGAACCGACTATGAATGGTACCAATAAAAGCGAGTATACTATTTCAAAAAGCAAATTATGATTTTGTTTTTTCTTTACAAGAATTACGAATACAAAGAGTAAGCCACATAAAATAAGTACCATAATCTGAATCAACAGAATCAATCATACACGACCACCAATTCTGGGTGTTTCATAATTGGGACAATCCTTGATGTAGAATATATACTCAGAGTTACAGAGTAAAATTCCTTTGCACACT

>Virtual iPCR amplicon spanning the AseI-NdeI junction (shaded) of paralog C.

TCTTCTACATCACCTGTTTCATTGTTTTGGTTATGCTACATTCTTAACCTTTCTGTTGCTCCGTCACTATAGAATAACACAACTCTCTCGTTTGGTTGAATAATGAGTCTTTAGACAGTAAATTCTATTTTTGAATTGATTTACAATATATTAGGTCAAAATAATTTATTTACAAATATATTAAATTAAAAGAATTTCAAACTCACACATAACAAAAAGGAATTATTTTACCCAAATAAAGAATATATTTAAAAAGTGAAAGGAATTATGAAATTGCAATGCCCGGAAATACATTCTTCCCCGAACCGATTATGAATGGTACCAATAAAAGCCAGTATACTATTTCAAAAAGCAAATTATGATTTTGTTTTTTCTTTGCAAGAATTACGAATACAAAGACTAAGCCACATAAAATAAGTACCATAATCTGAATCAACAGAATCAATCATACACGACTAATTCTGGGTTTTTCATAATTGGGACAATTGTTGATGTGCAATATATCCTCAGAGTTAATGAGTAAAATTCCTTTGCACACT

> Detected iPCR amplicon spanning the AseI-NdeI junction (shaded) of chimeric paralogs A/C from mutants R1-6 R1-18 and R1-19.

TCTTCTACATCACCTGTTTCATTGTTTTGGTTATGCTACATTCTTAAATTTTTTCTGTTGCTCCTTCACTGTAGAATAACACAACTCTCTCGTGTGTTTTAATAATGAGTCTTTAGACAGAAAATTCTATTTTTAAATTGATTTACAATATATTATATGTCAAAATAATTTATTTAGAAATATATTAAATTAAAAGAATTTCAAACTCACACATAACAAAAAGAATTATTTTACCCAAATAAAGAATATATTTAAAAACTGAAAGGAATTATGAAATTGCAATGCCCGGAAATACATTCTTCCCCGAACCGATTATGAATGGTACCAATAAAAGCCAGTATACTATTTCAAAAAGCAAATTATGATTTTGTTTTTTCTTTGCAAGAATTACGAATACAAAGACTAAGCCACATAAAATAAGTACCATAATCTGAATCAACAGAATCAATCATACACGACTAATTCTGGGTTTTTCATAATTGGGACAATTGTTGATGTGCAATATATCCTCAGAGTTAATGAGTAAAATTCCTTTGCACACT
